# Ultra-high Magnification Endocytoscopy and Molecular Markers for Defining Endoscopic and Histologic Remission in Ulcerative Colitis—An Exploratory Study to Define Deep Remission

**DOI:** 10.1093/ibd/izab059

**Published:** 2021-05-21

**Authors:** Marietta Iacucci, Louisa Jeffery, Animesh Acharjee, Olga Maria Nardone, Davide Zardo, Samuel C L Smith, Alina Bazarova, Rosanna Cannatelli, Uday N Shivaji, John Williams, Georgios Gkoutos, Subrata Ghosh

**Affiliations:** 1 Institute of Immunology & Immunotherapy University of Birmingham, Birmingham, UK; 2 Institute of Cancer and Genomic Sciences, Centre for Computational Biology, University of Birmingham, Birmingham, UK; 3 Institute of Translational Medicine, University of Birmingham, Birmingham, UK; 4 NIHR Surgical Reconstruction and Microbiology Research Centre, University Hospital Birmingham, Birmingham; 5 University Hospitals Birmingham NHS Foundation Trust, Birmingham, UK; 6 Institute for Biological Physics, University of Cologne, Cologne, Germany; 7 MRC Health Data Research UK (HDR), Birmingham, UK; 8 NIHR Wellcome Trust Clinical Research Facilities University Hospitals Birmingham NHS Trust, University of Birmingham, Birmingham, UK; 9 NIHR Biomedical Research Centre, University of Birmingham and University Hospitals Birmingham NHS Foundation Trust, Birmingham, UK; 10 APC Microbiome Ireland, College of Medicine and Health, University College Cork, Cork, Ireland

**Keywords:** mucosal healing, histological healing, endocytoscope, noninvasive markers, RNA-sequencing

## Abstract

**Background:**

Endoscopic and histological remission are both important treatment goals in patients with ulcerative colitis (UC). We aimed to define cellular architecture, expression of molecular markers, and their correlation with endoscopic scores assessed by ultra-high magnification endocytoscopy (ECS) and histological scores.

**Methods:**

Patients with UC (n = 29) were prospectively recruited. The correlation among ECS score (ECSS), Mayo endoscopic score (MES), and histological scores were determined. Area under curve were plotted to determine the best thresholds for ECSS that predicted histological remission by Robarts (RHI) and Nancy Histological Index (NHI).

Soluble analytes relevant to inflammation were measured in serum and mucosal culture supernatants using ProcartaPlex Luminex assays and studied by partial least square discriminant analysis and logistic model. Mucosal RNA sequencing and bioinformatics analysis were performed to define differentially expressed genes/pathways.

**Results:**

Endocytoscope scoring system correlated strongly with RHI (r = 0.89; 95% CI, 0.51–0.98) and NHI (r = 0.86; 95% CI, 0.42–0.98) but correlated poorly with MES (r = 0.28; 95% CI, 0.27–0.70). We identified soluble brain-derived neurotrophic factors (BDNF), macrophage inflammatory proteins (MIP-1 α) and soluble vascular cell adhesion molecule 1 (sVCAM-1) predicted histological remission. Mucosal biopsy cultures also identified sVCAM-1 associated with healed mucosa. RNA-seq analysis identified gene expressions shared between ECSS, RHI, or NHI defined healing. A number of gene expressions and pathways were identified including inflammation and metabolic and tumor suppressors that discriminated healed from nonhealed mucosa.

**Conclusions:**

Endocytoscopy represents an interesting tool that may sit between endoscopy and histology—but closer to the latter—identifying gene expression markers and pathways that are also identified by histology.

## INTRODUCTION

Achieving mucosal healing (MH) is considered to be a primary target in the treatment of inflammatory bowel disease (IBD).^1–3^ Mucosal healing increasingly incorporates both endoscopic remission (ER) and histologic remission (HR). Nevertheless, ER does not necessarily correspond to HR.^4–6^ Indeed, there is an increasing evidence that patients with ER have histological inflammation, accounting for relapse and adverse outcomes at follow-up.^7, 8^ This discrepancy between HR and ER might be explained by the use of conventional endoscopes that cannot accurately detect subtle inflammation.^9, 10^ Several studies have shown that HR is associated with reduced steroid use, lower risk of complication, hospitalization, and colorectal cancer development, suggesting it is an important endpoint to achieve—especially in ulcerative colitis (UC).^11–14^

The advanced endoscopy technologies are getting closer to histology by introducing the new concepts of mucosal and vascular healing patterns.^15, 16^ We have demonstrated that with widely available advanced technologies such as virtual electronic chromoendoscopy (VEC) and high definition (HD) imaging, endoscopic and histologic scores correlate more strongly and reduced discrepancy between the two, unlike previous publications.^17–19^ This has also been shown using artificial intelligence approach utilizing deep neural network approach.^20, 21^ Among these new endoscopic armamentarium, endocytoscopy (ECS; CF- Y-0058-1 prototype, Olympus Japan) is a new technique that provides in vivo microscopic imaging during endoscopy, with ultra-high magnification ranging from 450-fold to 1400-fold, looking at cells and nuclei of mucosal surfaces. Several studies have reported that ECS is a reliable technique to assess precisely ER and HR, potentially reducing the need for biopsy specimens. Notably, biopsies can assess only a limited area, whereas ECS is an optical diagnosis tool, which can sample a wider area in vivo of the colonic mucosa.^22^ Studies with ECS have shown high reproducibility between endoscopists and demonstrated that it predicts accurately histological inflammation and HR.^22–25^ Therefore, ECS is a bridge between endoscopy and histology, but further studies are required.

The identification of noninvasive molecular markers to monitor IBD patients by predicting MH and the risk of flare-up is a growing area of interest. Frequent endoscopic examinations are costly and uncomfortable for the patient; therefore, a need exists for blood-based biomarkers that accurately assess MH, which correlates well with endoscopic findings.^26^ Recently, a commercial panel based on blood levels of 13 proteins, called the endoscopic healing index (EHI), was developed and preliminary evidence supports that it can identify endoscopic healing, favoring the noninvasive monitoring and management of Crohn’s disease (CD) patients.^27^ Despite the encouraging results of EHI, it did not include histological assessment to confirm MH and has not been reproduced in UC.

Though many studies have explored the gene expression profiles in the context of specific therapies,^28, 29^ few studies have investigated ultrastructural and molecular mechanisms of intestinal healing beyond absence of inflammation. Whether advanced endoscopic techniques such as ECS and histology share molecular footprints of intestinal restitution and repair requires further evidence. Importantly, MH is not just an absence of inflammation but an active process of restitution and repair at the cellular and molecular level.^30^

The main objectives of this exploratory study were to understand ER and HR in UC determined by ultra-high magnification in vivo microscopy ECS, determine the correlation between endocytoscopy and histology, and define cellular architecture, expression of molecular and genomic markers and their correlation with endoscopic score defined by ultra-high magnification with histology scores.

## MATERIALS AND METHODS

### Patients

We conducted a prospective study enrolling 29 consecutive UC patients (18 males, 62%; mean age 41 years ± SD 15) referred for assessing ER achieved after treatment or for surveillance between January 2018 and July 2019 at a tertiary academic center (Queen Elizabeth II Hospital, Birmingham, UK).

We collected demographic data including patient characteristics, clinical data detailing age at diagnosis, disease characteristics (extent of disease, Montreal classification), treatments (corticosteroids, immunosuppressive therapy, biologics), clinical disease activity scores (full and partial Mayo score^31^), and endoscopic activity scores (Mayo endoscopic score; MES),^32^ ulcerative colitis endoscopic index (UCEIS),^33^ and Paddington International virtual chromoendoscopy score (PICaSSO)^34–36^ (Table 1).

**TABLE 1.** Demographic and Clinical Data of IBD Patients Enrolled

UC	Demographic and Clinical Characteristics
Sex	11 F; 18 M (62%M and 38% F)
Mean age ± SD (range)	41± 15 (20–66)
Disease duration median years (range)	12 (1–38)
Localization	
Pancolitis (E3)	25 (86%)
Left colitis (E2)	4 (15%)
Proctosigmoiditis (E1)	0
Mayo endoscopy score (MES)	
Mayo 0	11 (38%)
Mayo 1	8 (27.5%)
Mayo 2	8 (27.5%)
Mayo 3	2 (7%)
Clinical Mayo	
Remission < 2 remission	15 (52%)
Mild 2–4 mild activity	7 (24%)
Moderate 5–7 moderate activity	3 (10%)
Severe > 7 severe activity	4 (14%)
Biological therapy	
Adalimumab	3 (10.3%)
Infliximab	1 (3.5%)
Vedolizumab	3 (10.3%)
Ustekinumab	1 (3.5%)
No biological therapy	21 (72.4%)
Medication	
Mesalazine	24 (83%)
Steroids	7 (24%)
Immunosuppressants	6 (20%)
CRP mean (range) mg/dL	9 (1–33)
FC mean (range) mcg/gram	656 (30–2363)

### Endoscopic Procedure

All the recruited patients underwent colonoscopies with endocytoscope (ECS; CF- Y-0058-1 prototype Olympus, Japan) with 520-fold magnification to obtain ultra-magnified images and define inflamed and healed areas. For consistency of technique, all of the procedures were performed by one colonoscopist (MI) experienced in ECS and optical diagnosis in IBD to harmonize and accurately assess the grade of inflammation. Additionally, the endoscopic findings were scored by 2 endoscopists during the procedure and also video recorded. The agreed score between the 2 endoscopists was recorded and analyzed.

**FIGURE 1.**
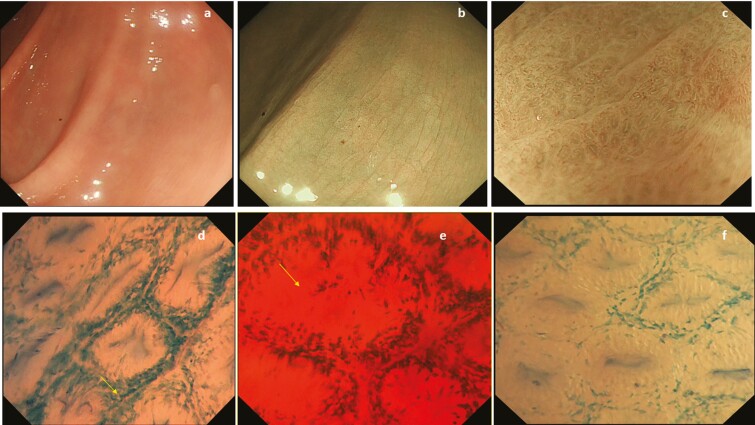
A–C, Endoscopic MH assessed by HD white light endoscopy and NBI with and without magnification showing elongated crypts as observed in endoscopic mucosal healing in UC. D–F, Ultra-high magnification endocytoscope after using dye chromoendoscopy with methylene blue 1% showed increase infiltration with cells between the crypts (arrow) and elongated distorted crypts (arrow) as per histological healing changes.

The colonic mucosa was initially assessed using HD white light endoscopy (HD WLE) and narrow banding imaging (NBI) with and without magnification. The inflammatory activity was scored using the MES, and ER was defined as MES 0.^2^ Biopsies were taken targeting either the worst affected area of inflammation or the most representative area of endoscopic healing. After washing of the mucosa with water plus simethicone and staining with 10 mL of 1% methylene blue solution, ECS was performed in the same area of the colon where endoscopic activity was scored. The following endoscopic parameters for ECSS were assessed to grade the inflammation (Table 2): (1) the shape of the crypts (normal round, elongated, irregular, necrosis); (2) infiltration of the cell between the crypts (≤50% vs ≥50%); (3) the distance between the crypts (normal, 3 or more crypts in a visual field [VF], ≤2 crypts in a VF, intermediate 2≤ or ≥ 3 crypts in a VF with infiltrating cells in lamina propria (LP), dropout/necrosis); and (4) the visibility of superficial microvessels (not visible vs visible).We set an overall score of 3 to 9, and we defined the following subscores: the shape of crypts (range, 1–3), infiltration between the crypts (range, 1–2), distance between the crypts (range, 1–3), and the visibility of superficial microvessels (range, 0–1). This score was adapted from Nakazato et al by including endoscopic findings representative of disease activity (infiltration of cells, Fig. 1).^22^

**TABLE 2.** Endocytoscopy Scoring System

Endocytoscopy Items	Score
Crypts architecture	
Normal, elongated	**1**
Irregular	**2**
Necrosis	**3**
Infiltration of the cell between the crypts	
≤50%	**1**
≥50%	**2**
Distance between the crypts	
Normal: 3 or more crypts in a VF	**1**
Elongated = <2 crypts in a VF	**1**
Intermediate = 2 ≤ crypts ≥ 3 in a VF with infiltrating cells in LP	**2**
Drop-out /necrosis	**3**
Visibility of superficial microvessels	
Not visible	**0**
Visible	**1**
ECS total score	**3–9**

Pictures and videos collected under HD WLE, NBI, and then ECS from each patient were all recorded and digitally stored for subsequent analyses by using the Olympus recording system IMH-20.

### Histological Scoring

Histological assessment of inflammatory activity was scored by an expert pathologist (DZ) blinded to clinical and endoscopic information. The following histological scores were used: NHI score (range, 0–4)^37^ and Robarts Histological Index (RHI,^38^ range, 0–33). Histologic remission was defined as NHI ≤1^37^, and RHI ≤3 without neutrophils in the epithelium and lamina propria.^39^

### Analysis of Soluble Markers

#### Serum collection

Serum samples were collected from all patients at the time of the colonoscopy in BD SST vacutainer tubes and processed within 4 hours. Samples were spun at 1300 *g* for 10 minutes and serum removed and stored at −80C before analysis.

#### Culture of mucosal biopsies

Endoscopic biopsies were cultured in 10% fetal bovine serum, (50 U/mL Penicillin/Streptomycin, 200 μM L-Glutamine, 50 μg/mL Gentamicin, 2.5 μg/mL Amphotericin B, 50 μM 2-mercaptoethanol in RPMI 1640) at mass to volume ratio of 20 ug/mL. At 24 hours, supernatants were harvested, centrifuged for 10 minutes at 10000 g and stored at −80C before analysis.

Serum and mucosal biopsy culture supernatants were thawed on ice and analyzed for 56 soluble markers (Table 3) using ProcartaPlex Luminex assays (ThermoFisher Scientific, Waltham, Massachusetts, US) according to manufacturer guidelines. Serum samples were diluted 2-fold for all analytes except for matrix metalloprotein-2 (MMP-2), matrix metalloprotein 9 (MMP-9), Intercellular Adhesion Molecule 1 (ICAM-1), and soluble vascular cell adhesion molecule 1 (sVCAM-1), which were diluted 100-fold. Culture supernatants were used neat.

**TABLE 3.** ProcartaPlex Luminex Arrays Used to Analyze Serum and Endoscopy Biopsy Culture Supernatants

ProcartaPlex Array	Analytes
4 Plex (customized)	MMP-2; MMP-9; sICAM-1; sVCAM-1
7 Plex (customized)	BLC; IL-12p40; MMP-1; OSM_ a,b_; TNFR1; TNFR2; TREM-1_a,b_
45 Plex (ProcartaPlex Hu) Cytokine/Chemokine/GF 1 45plex (Cat no. EPX450-12171–901)	BDNF_b_; Eotaxin/CCL11; EGF_b_; FGF-2_a,b_; GM-CSF_a_; GRO alpha/CXCL1_a_; HGF; NGF beta_ a,b_; LIF; IFN alpha_ a,b_; IFN gamma_a_; IL-1 beta_ a_; IL-1 alpha_ a_; IL-1RA_ a_; IL-2_ a_; IL-4_ a,b_; IL-5_ a,b_; IL-6_ a_; IL-7; IL-8/CXCL8_ a_; IL-9_ a,b_; IL-10_ a_; IL-12 p70_ a_; IL-13*; IL-15_ a_; IL-17A_ a_; IL-18_ a_; IL-21_ a,b_; IL-22_ a_; IL-23_ a,b_; IL-27_ a_; IL-31_ a,b_; IP-10/CXCL10; MCP-1/CCL2; MIP-1 alpha/CCL3; MIP-1 beta/CCL4; RANTES/CCL5; SDF-1 alpha/CXCL12; TNF alpha_ a_; TNF beta/LTA_ a,b_; PDGF-BB_b_; PLGF; SCF; VEGF-A; VEGF-D_ a,b_

^a^Not detected in serum

^b^Not detected in culture supernatant

### RNA Extraction, Library Preparation, and Sequencing

Endoscopic biopsies of the colon were stored in RNA later before RNeasy on-column RNA extraction and purification (Qiagen, Venio, Netherlands). RNA was quantified by Qubit (Life Technologies, Carlsbad, California, US) and 0.5 ng used to prepare uniquely indexed cDNA QIAseq UPX 3’ Transcriptome libraries according to manufacturer’s instructions. Libraries were quantified and quality controlled using the QIAseq Library Quant Assay Kit and tapestation analysis and sequenced on the Miseq and Nextseq Illumina platforms to a depth of 1 to 3milion reads/sample. Fastq files were obtained through BaseSpace and reads demultiplexed aligned, quantified, and normalized using the CLC Genomics Workbench (Qiagen, Venio, Netherlands). The RNA seq data was deposited, and the raw data is available at Array Express through accession number E-MATB-9731.

### Statistical Analysis and Informatics

#### Demographic, clinical and endoscopic data

Demographic, clinical, and endoscopic data were collected using the REDCap platform, and the results were transferred to a Microsoft Excel database.

Pearson correlations between MES, ECSS, and histological scores and between Picasso total score, UCEIS, and ECSS were calculated. Very strong correlation was considered as a value of 0.8 to 1.0, strong as 0.6 to 0.79, moderate as 0.40 to 0.59, and weak as 0.2 to 0.39

We determined diagnostic accuracy, sensitivity, and specificity of MES and ECSS assessed by HD-NBI to predict HR. Receiver operating characteristic (ROC) curves were plotted as sensitivity vs specificity to explore the ability of endoscopic scores to predict HR. The area under the receiver operating characteristic curve (AUROC) was determined to accurately identify the cutoff of MES and ECSS that reflect HR.

#### Cytokine panel analysis from blood samples

A total of 56 soluble analytes relevant to inflammation or shown to be altered in UC were measured in serum (Table 3). Measurements that did not fall within the standard range of the assay were assigned the maximum or lower limits of the assay as appropriate, and analytes that were not detected in at least 40% of patients in 1 group were excluded from analysis to reduce type 1 errors. Those not detected in serum or culture supernatants are marked in Table 3. Detected analytes were used to train a logistic regression model.

#### Luminex soluble marker analysis from mucosal culture supernatants

Luminex data were initially analyzed using *t* test across all the comparisons separately. The selected analytes were then included in a regression model utilising partial least square discriminant analysis (PLS-DA). A false discovery rate (FDR) corrected *P* value (q value) was considered for significance. Significant cytokines/soluble markers were then further analyzed using PLS-DA modeling.^40^ We used variable importance in projection (VIP) to prioritize the features. Variable importance in projection is a measure of a variable’s importance in the PLS-DA model. It summarizes the contribution a variable makes to the model. Markers with a VIP score of more than 1.5 were used.^41^ Area under the curve for each comparison was calculated to show the sensitivity and specificity of each combinations of the markers.

#### RNA sequencing data

Bioinformatics analysis of the RNA sequencing data was performed (AA, GG). The following differential expression procedure was performed for participants classified as healed vs nonhealed according to each of the scoring procedures. Transcripts per million (TPM) normalized values^42^ were log-transformed with a pseudocount of 1 added. Transcripts with <1 transformed count in any sample were excluded from further analysis, as were transcripts with low variance (defined as less than 10% unique counts across both conditions and greater than a 19:1 ratio of the most frequent count to the second most frequent across both conditions). Differential expression analysis between conditions was conducted with the limma package.^43^ Library size was estimated using reduced maximum likelihood estimator with 500 iterations. Initial fitting was performed using a robust M-estimation, and moderated test statistics were computed by empirical Bayes. An FDR-corrected *P* value <0.05 was considered and filtered for further downstream data analysis. Partial least squares discriminant analysis modeling was performed on these genes. A VIP score was used to further filter the genes and measured a variable’s importance in the PLS-DA model. Genes with a VIP score of more than 1 were used for gene expression analysis. To understand the biological significance and pathways, enrichment analysis using EnrichR was also performed.^44^ Pathway enrichment was done using gene ontology and kyoto encyclopaedia of genes and genomes (KEGG).^45^ Differentially expressed up and down regulated genes were visualized using volcano plots. We considered Fold change >2.0 and FDR-corrected *P* value < 0.05 across healed vs nonhealed samples.

The RNA sequencing data were loaded into ArrayExpress (ArrayExpress accession:E-MTAB-9731).

## ETHICAL CONSIDERATIONS

The study was approved by the research ethics committee (IRAS ID: 227882), and all patients provided written informed consent. Exclusion criteria included inability to provide consent, coagulation defect, or severe comorbidity.

## RESULTS

### Patient Characteristics

The demographic characteristics of the patients are summarized in Table 1. Eighty-six percent (25 of 29) of UC patients had pancolitis, and 52% (15 of 29) were in clinical remission according to partial Mayo clinical score. Although, 38% (11 of 29) showed an MES of 0, and 28% (8 of 29) showed an MES of 1; 45% (13 of 29) and 52% (15 of 29) were in histologic remission (HR) according to NHI ≤1^37^ and RHI ≤3,^39^ respectively.

### Correlations of Endocytoscopy and Histology (NHI and RHI) Scores in UC

The ECS total score had very strong correlation with HR as defined by NHI r = 0.86 (95% CI, 0.42–0.98) and RHI r = 0.89 (95% CI, 0.51–0.98). Whereas ECS total score correlated weakly with MES r = 0.28 (95% CI, 0.27–0.70). Mayo endoscopic score correlated strongly with NHI r = 0.73 (95%CI 95% 0.35–090) and RHI r = 0.63 (95% CI, 0.18–0.86), though less strongly than with ECSS. (Table 4)

**TABLE 4.** Correlations of ECS With NHI and RHI Score in UC Patients

Correlations of Endocytoscopy Scores and NHI Score of UC Patients	
Crypts architecture	76.4%; 95% CI, 12.9- 95.4
Infiltration of the cell between the crypts	66.1%; 95% CI, 8.11–93.2
Distance between the crypts	86.6%; 95% CI, 41.52–97.5
Visibility of vessels	75.0%; 95% CI, 9.61–95.2
Endocytoscopy total score	86.6%; 95% CI, 41.5–97.5
Correlations of Endocytoscopy Scores and RHI Score of UC Patients	
Crypts architecture	66.3%; 95% CI, 7.7–93.2
Infiltration of the cell between the crypts	82.7%; 95% CI, 29.4–96.7
Distance between the crypts	89.6%; 95% CI, 52.05–98.1
Visibility of vessels	86.7%; 95% CI, 41.9–97.6
Endocytoscopy total score	89.3 %; 95% CI, 50.8–98.0

### Correlations of Endocytoscopy and Endoscopic PICaSSO and UCEIS Scores in UC

The ECS total score showed a strong correlation with PICaSSO total score r = 0.67 (95% CI, 0.40–0.83) and with UCEIS r = 0.74 (95% CI, 0.51–0.87), better than MES but weaker than HR defined by NHI.

### Diagnostic Accuracy of Endocytoscopy Score System (ECSS) in UC Patients for RHI ≦**3 and NHI** ≦1

Using the defined threshold of HR, we analyzed the diagnostic accuracy of each ECSS endoscopic feature, the total score in the colonic area, and from where biopsies were taken. (Tables 4, 5). A ROC curve was used to calculate the best value of total ECSS to predict HR. The best value of ECSS total score was ≤3 for predicting HR, with RHI ≤3 with no neutrophilis in epithelium/LP, with an AUROC of 0.81 (95% CI, 0.66–0.97), specificity of 0.89 (95% CI, 0.52–1), sensitivity of 0.62 (95% CI, 0.31–0.86), and an accuracy of 0.80 (95% CI, 0.57–0.87). Similarly, an ECSS total score of ≤3 was the best cutoff to predict HR defined as NHI ≤1, with an AUROC of 0.77 (95% CI, 0.59–0.95), specificity of 0.86 (95% CI-43-1), sensitivity of 0.64 % (95% CI, 27–91), and an accuracy of 0.80% (95% CI, 49–90). (Tables 5, 6)

**TABLE 5.** Diagnostic Accuracy of the Best Threshold ECSS Total to Predict Histological Healing

UC	Sensitivity 95% CI	Specificity 95% CI	Accuracy 95% CI	AUROC 95% CI
ECS total score ≤3 and RHI ≤3	61.5% (31–85)	88.5 % (52–100)	79.5% (57–87)	81.2% (66–97)
ECS total score ≤3 and NHI ≤1	64% (27–91)	86% (43–100)	79.5% (49–90)	77% (59–95)

**TABLE 6.** Diagnostic Accuracy of the Best Threshold of Each ECS ≤3 Item to Predict Histological Healing in UC Defined by RHI and NHI Scores

ECS Item in UC	Sensitivity 95% CI	Specificity 95% CI	Accuracy 95% CI	AUROC 95% CI
Crypt architecture ≤0 and RHI ≤3	46% (11.5–71)	96% (52–100)	80% (50–82.5)	74% (57–91)
Infiltration of the cell ≤1 between the crypts and RHI ≤3	69% (33–92)	81.5% (42-–94)	77.5% (51–85.5)	75% (60–90)
Distance between the crypts ≤1 and RHI ≤3	92% (69–100)	56% (8–75)	67.5% (36–82)	82% (68–95.5)
Visibility of superficial microvessels ≤0 and RHI ≤3	92% (64–100)	48% (15–67)	62% (52.5–65)	70% (57–83)
Crypt architecture ≤0 and NHI ≤1	45.5% (12–74)	93% (52–100)	80% (50–85)	72% (54–91)
Infiltration of the cell ≤1 between the crypts and NHI ≤1	64% (29–91)	76% (39–91)	72.5% (46–83)	70% (53–87)
Distance between the crypts ≤1 and NHI ≤1	91% (65–100)	52% (8–72)	62.5% (31–78)	78% (62–94)
Visibility of superficial microvessels ≤0 and NHI ≤1	57% (49–59)	90% (61–100)	44% (15–63)	67% (54–81)

### Serum Soluble Markers

**FIGURE 2.**
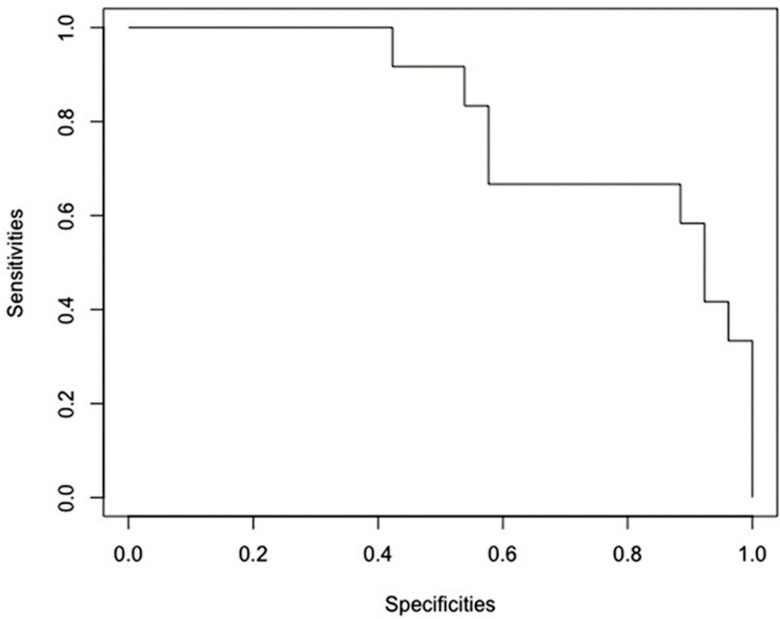
ROC curves of serum BDNF and MIP-1 that, when combined together, were best at predicting histological healing (RHI ≤3 and NHI ≤1) with an AUROC of 0.82.

By downstream modeling and logistic regression, we identified brain-derived neurotrophic factors (BDNF) and macrophage inflammatory proteins (MIP-1 α) that, when combined together, showed the highest predictive ability for RHI ≤3 (no neutrophils) and NHI ≤1 with an AUROC of 0.82 (95% CI, 0.69–0.97) (Fig. 2). When we investigated analytes separately for predicting HR, univariate logistic regression was significant for 2 other markers: leukemia inhibitory factor (LIF) and soluble vascular cell adhesion molecule 1 (sVCAM1), both showing higher values associated with active inflammation by histology. The AUROC was 0.74 for LIF (95% CI, 0.591–0.89) and 0.72 for sVCAM1 (95% CI, 0.53–0.90). Serum soluble markers did not correlate significantly at *P* < 0.05 with ECSS (LIF vs ECSS r = 0.31; sVCAM vs ECSS 0.37).

### Culture Supernatant of UC Mucosal Biopsies

The supernatants from the mucosal biopsy cultures were analyzed for a panel of soluble markers. The following markers were identified as healed or nonhealed mucosa. (Table 7). Both RHI ≤3 and NHI ≤1 identified the same set of molecules—MMP9, TNFR2, IL-1RA, and hepatocyte growth factor (HGF)—as a panel associated with HR. Area under the receiver operating characteristic curve was 0.82 (95% CI, 0.5–1) for a panel of 4 analytes to predict HR by RHI or NHI. The ECSS score ≤3 identified sVCAM-1, TNFR2, and IL-1Ra as a panel—the latter 2 being the same identified by HR—for predicting healing with an AUROC of 0.75 (95% CI, 0.25–1). For prediction of ER assessed by MES = 0, the AUROC was 0.91 (95% CI, 0.5–1) for 4 analytes as a panel of sICAM-1, IL-6, IL-1Ra, and IL-15.

**TABLE 7.** Biopsy Culture Derived Selected Makers From PLS-DA Modeling and Combined Marker AUC Values. Markers Were Selected Based on VIP >1.5

Score used	Markers Selected From PLS-DA Model (VIP > 1.5)	AUC (95% CI)
RHI	MMP-9, TNFR2, IL-1ra, HGF	0.82 (0.54–1)
NHI	MMP-9, TNFR2, IL-1ra, HGF	0.82 (0.5–1)
Mayo	sICAM-1 IL-6 IL-1ra IL-15	0.91 (0.5–1)
ECCS	sVCAM-1, TNFR2, IL-1ra	0.75 (0.25–1)

### RNA Sequence Analysis

**FIGURE 3.**
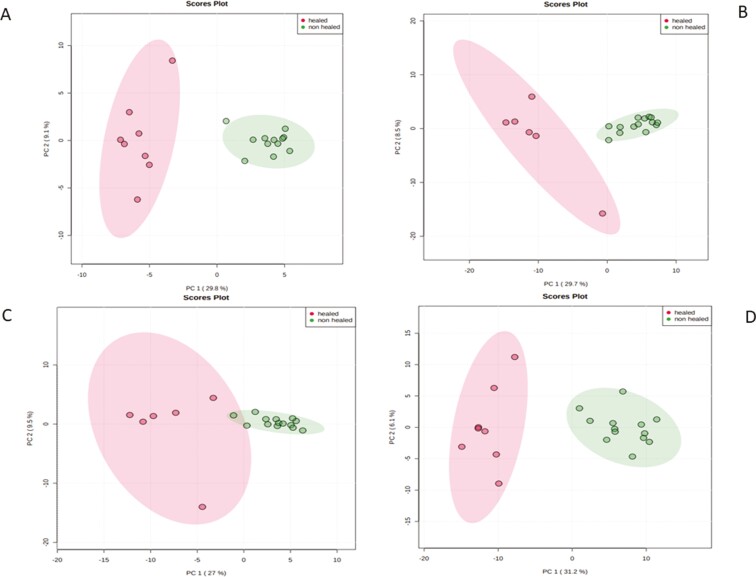
Principal component analysis score plots represented on the differentially expressed transcriptome datasets. Each of the plots demonstrating clustering of patients according to the healed vs nonhealed categories. Each of the dots (red or green) represent samples (or patients) and are colored according to the subject cohort (healed vs nonhealed). Ellipses represent 95% confidence healed or nonhealed patients. Results are plotted according to the top 2 principal components scores: principal component 1 (PC1) and principal component 2 (PC2). PC1 and PC2 scores, with the percentage variation explained by the *x* and *y* axis. Four different definitions of the healed vs nonhealed defined as (A) RHI scores, (B) NHI, (C) ECCS scores, and (D) Mayo score.

**FIGURE 4.**
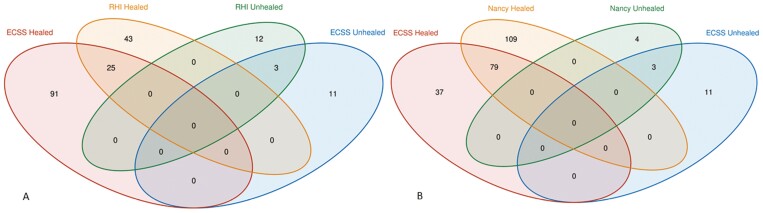
Overlap of upregulated genes in healed and nonhealed mucosa as defined by (A) ECSS and RHI scores, and (B) ECSS and NHI scores.

We performed RNA sequence analysis using each of the scores (MES, ECSS, RHI, NHI) and identified upregulated and downregulated genes in each of the outcome variables (Supplementary Table 1). Moreover, principal component analysis (PCA) shows clear separation of the healing vs nonhealing samples for all the scores, including ECSS, MES, RHI, and NHI index scores (Fig. 3). Comparing ECCS scores healing vs nonhealing, we found 116 genes upregulated and 14 genes downregulated. By RHI scores, 68 genes were upregulated and 15 genes were downregulated. Nancy Histological Index scores for healing vs nonhealing resulted in 188 upregulated and 7 downregulated genes. Those commonly identified by ECSS and RHI scores are summarized by the Venn diagram in Fig. 4. Twenty-five genes were overlapping between healed mucosa defined by ECSS and RHI score; 79 genes were overlapping between healed mucosa defined by ECSS and NHI index. (Supplementary Table 2)

We listed all the important genes prioritized by variable importance in projection of more than 1 in the PLS-DA model. We found that healing and nonhealing samples were separated by differentially expressed genes. For healing vs nonhealing comparison as defined by RHI, NHI, Mayo and ECCS, there were 32, 83, 127, and 60 differentially genes respectively (Supplementary Table 3). All these genes were highly predictive. For example, the AUC value using 60 genes together for healing vs nonhealing defined by ECCS was 1. The AUC value for each of the genes is listed in the Supplementary Table 4.

**FIGURE 5.**
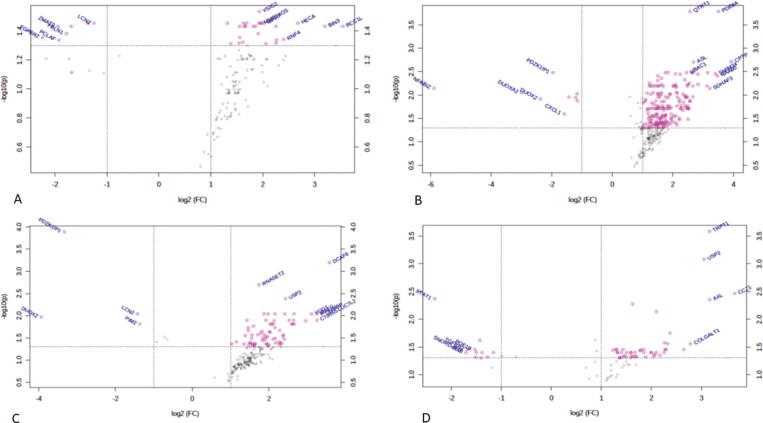
Volcano plot representations of the differentially expressed genes in healed vs nonhealed mucosa as defined by (A) ECSS, (B) Mayo, (C) NHI, and (D) RHI scores.

As shown by the volcano plots in Figure 5 these included genes relevant to TGF- β signaling such as TGFBR2, PDZK1IP1, USP2, and YOD1 and macrophage recruitment into tissues such as RNASET2, neutrophil and plasma cell function RNF4 and PIM2, and tumor suppressor genes HECA and BIN3. A number of those identified were shared by MES and ECSS defined healing, in addition to histological criteria defined healing (NHI and RHI).

### Gene Enrichment Analysis

**FIGURE 6.**
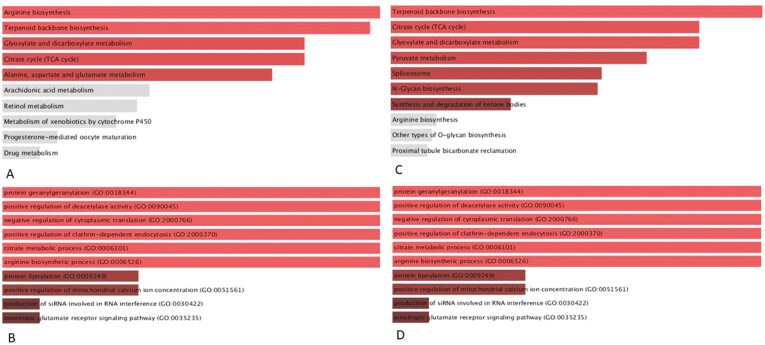
GO and KEGG pathway enrichment analysis of commonly upregulated genes in healed mucosa defined by ECSS and RHI scores (A and B) and ECSS and NHI scores (C and D). Pathways for which *P* < 0.05 are shaded red.

To investigate the roles of the gene pathways that might be involved in the healing process, gene enrichment analysis on the overlapping genes with KEGG and gene-ontology (GO) was used. Supplementary Tables 5 and 6 summarize the enriched pathways (*P* < 0.05) identified by KEGG and GO biological process analysis, respectively, for the differentially expressed genes as defined by each score. Those identified for the 25 differentially expressed genes that were commonly upregulated according to ECSS vs RHI scores and the 79 genes commonly upregulated between ECSS and NHI scores are illustrated in Figure 6 and summarized in Supplementary Table 7. In summary, the constitutive androstane receptor (CAR) and pregnane x receptor (PXR) pathways—in addition to the metabolic pathways related to aminoacids, terpinoid, and andarachidonic acid and the tricarboxylic acid (TCA) cycle were identified and considered mechanistically plausible. The biological relevance of these pathways to MH is presented in the discussion.

## DISCUSSION

Both ER and HR are considered therapeutic targets to prevent long-term complication in IBD. However, ER may not always translate accurately to HR, especially when previous generation of endoscopes is used. Although with the current generation of HD and VCE endoscopes, discrepancy between endoscopy and histology is smaller.^17^ Advances in endoscopy technologies have dramatically improved the way to assess the intestinal mucosa, allowing in vivo microstructural mucosal features to be visualized. The endocytoscope, although only available in limited number of centers, currently operates in a similar way to a standard endoscope and can be switched to electronic chromoendoscopy and ultra-high magnification in vivo microscopic mode at the press of a button. For ultra-high magnification, methylene blue spray is required, a technique familiar to gastroenterologists for dye spray endoscopy.

In this study, we explored whether the latest generation endocytoscope with ultra-high magnification can accurately assess subtle inflammatory changes in the colonic mucosa and better determine HR of patients in UC. We confirmed that mucosal ER defined by the latest-generation endocytoscope does correlate strongly with HR in UC. The best value of ECSS for predicting HR with RHI and NHI was ≤3. We further sought to detect the best predictors among each endocytoscope score items for their ability to differentiate between MH and mild inflammation. The best predictors were the distance between crypts and total ECSS (Table 4). Similarly, Natazako et al showed a good diagnostic accuracy of ECSS, with sensitivity of 77% (95% CI, 59–89), specificity of 97% (95% CI, 83–99) ,and accuracy of 86 % (95% CI, 75–93) to predict HR in patients with UC.^22^ Bessho et al developed the first ECSS score and showed good correlation of each item and histopathological grade.^25^ Therefore, this study also confirmed that endocytoscopy features such as crypt architecture, distance between crypts, cellular infiltration, and visibility of microvessels at endoscopy were strongly correlated with histological scores. Furthermore, each of these features could accurately predict histology defined using the validated scores such as RHI and NHI for UC. (Tables 4–6).

Of note, Maeda et al described a computer-aided diagnosis (CAD) endocytoscopy system to predict persistent histologic activity.^46^ It is likely that CAD diagnosis may also be related to long-term clinical prognoses. However, this requires a prospective longitudinal follow-up study with specific therapies. We have recently reported that, even with high definition and electronic chromoendoscopy, endoscopic remission and histologic remission equally predict clinical outcomes at 1 year.^36^ Thus endoscopy is getting closer to histology and AI might enable efficient use of endocytoscopy with minimal training and time.

We also investigated if the introduction of soluble markers in blood could provide a noninvasive method to predict HR in UC. We found that serum levels of BDNF + MIP-1α predicted HR defined by histological scores of RHI and NHI. Leukemia inhibitory factor and sVCAM1 showed higher values associated with active disease by histology. Interestingly, BDNF and MIP-1α have been associated with healing in different tissues and derived from specific subsets of macrophages and plasma cells and so could be mechanistically relevant.

In this study, sVCAM-1 concentration was related to HR in UC. A mechanistic explanation might be that mucosal VCAM-1 adheres to monocyte-expressed α4β7 integrin and directs in vivo gut homing.^47^ This facilitates recruitment of subtypes of macrophages that have been identified as important in restitution and repair in IBD, and hence, soluble markers such as sVCAM and MIP-1α are of interest.^30^ In the future, this may minimize and avoid invasive procedures to monitor response to therapy and assess MH. However, in this study soluble markers were not compared with fecal calprotectin. Recently, a new Monitr serological test was developed to assess mucosal inflammation by evaluating 13 biomarkers in CD patients.^27, 48^ There is no obvious overlap between this panel in CD and our panel in UC, except VCAM and further large studies are required to replicate and validate these preliminary findings.

A panel of cytokines and soluble proteins from mucosal biopsy cultures that could predict MH by endoscopic, endocytoscopic, and histologic criteria was also identified (Table 4). This was aimed at mechanistic assessment of MH and the process of mucosal restitution and repair. Several of these molecules (IL-1Ra, TNFR2) are associated with healing by antagonising inflammatory cytokines.^49, 50^ Matrix metalloproteinase (MMP) 9 has been demonstrated to be involved in wound healing and angiogenesis. Matrix metalloproteinase has been shown to be involved in intestinal healing in mice. It has also been implicated in inflammation, though blocking MMP9 did not improve active UC.^51^ Matrix metalloproteinases including MMP9 play a role in tissue remodeling, but further studies are required regarding MMP9 in MH.^51, 52^ Notably, MMP9 expression regulates epithelial barrier function, as evident by decreased paracellular permeability and reorganization of claudins; it also acts as a tumor suppressor in colitis-associated cancer by sustaining the epithelial mucosal integrity through/ the activation of EGFR-Sp1 signaling pathway.^52^

Hepatocyte growth factor (HGF) is a paracrine multifunctional protein involved in angiogenesis and regeneration of tissues.^53^

It is of note that sVCAM-1 was associated with HR in both the serum and biopsy culture supernatants.

RNA-seq analysis identified genes expressed in colonic biopsies that discriminated between healed and nonhealed mucosa and overlapped gene expression between ECSS defined healing and histology defined healing (Figs 4–5). These gene expression profiles relate to a number of metabolic pathways involved in vital tissue functions that may be damaged in inflammation related to tissue destruction (Fig. 6 and Supplementary Table 7) or involved in intestinal restitutive and repair processes triggered by damage.

Partial least squares discriminant analysis, volcano plot, gene enrichment, GO analysis, and KEGG analysis identified a number of genes and pathways that are biologically plausible to be involved in MH. It is interesting that a number of tumor suppressor genes are upregulated in healed mucosa (HECA, BIN3) compared with nonhealed inflamed mucosa. This may be relevant to dysplasia risk. In this study, the TGF-β pathway, neutrophil function, and macrophage recruitment were identified as important in defining healing by histological indices (RHI and NHI) and by ECSS. (Fig. 5). In addition, biological processes and pathway enrichment (GO, KEGG, and WIKI) identified a number of molecular functions and pathways that may be relevant for MH including the pregane X receptor (PXR)-JNK axis, which has been shown to be involved in healing in other organ such as skin.^54^ Constitutive androstane receptor (CAR) pathway regulates intestinal mucosal response to injury^55^ in mice. Activation of PXR, which is a close relative of CAR, may also enhance intestinal epithelial wound healing.^56^ Therefore, a small molecule inhibitor of the xenobiotic receptor CAR (eg, CINPA1^57^) may be useful in MH and needs further translational studies. In addition to these pathways, this study identified several metabolic pathways including those involving arachidonic acid, amino acids, terpenoid biosynthesis, and the TCA cycle, whose involvement in healing is already recognized.^51, 53, 54^ Interestingly, the involvement of amino acids histidine and arginine in intestinal cell restitution may also involve TGF-β pathways.^58^ Transforming growth factor-β promotes protein translation at least in part by increasing the mitochondrial oxidation of glucose and glutamine carbons to support the energy demand of translation. In addition to stimulating the entry of glucose and glutamine carbon into the TCA cycle, TGFβ induced the biosynthesis of proline from glutamine in a Smad4-dependent fashion.^59^ Oxidoreductase activity is an important molecular function in intestinal healing due to intestinal damage resulting from neutrophil-derived reactive oxygen.^60^ Terpenoids are bioactive and can help anchor proteins to cell membranes and are shown to affect wound healing.^61^

The strengths of this study include the use of ultra-high magnification in in vivo microscopy to identify ultrastructural features of ER, the application of 2 validated histology scores (RHI and NHI), the study of molecular markers from mucosal biopsy culture, and the RNA sequencing analysis of mucosal biopsies and bioinformatics to identify potential molecules and molecular pathways that are relevant. In addition, this study has examined potential molecular pathways and soluble markers that might be relevant to the healing process and therefore indicators of healing or targets for therapy. The MES acted as a comparator of a routinely used endoscopy score in clinical practice, which poorly correlated with ECSS.

Limitations of the study include a relatively small number of patients giving large confidence intervals for results; thus validation in a larger independent cohort is needed. There were no fecal calprotectin assays from any patients, as this was not an aim for this study, but this biomarker is reported extensively in the context of advanced endoscopies recently.^62^ This study did not investigate CD patients, though mucosal biopsies may not represent molecular events in CD and UC. Of note, there was no focus on the mechanistic effect of the selected genes on the mucosal architecture because it was beyond the scope of this exploratory study; it will require minigut organoids, molecular imaging, and animal models using conditional gene knockdowns—a task for future studies.

In conclusion, ultra-high magnification endocytoscopy score strongly correlated with either RHI or NHI but not with MES. A number of soluble markers were identified, which could predict HR, including molecules such as sVCAM1, which was elevated in both peripheral blood and mucosal biopsy cultures. RNA transcriptomics analysis identified differentially expressed genes that were shared between ECSS and healing defined by histological score.

## Supplementary Material

izab059_suppl_Supplementary_Table_1

izab059_suppl_Supplementary_Table_2

izab059_suppl_Supplementary_Table_3

izab059_suppl_Supplementary_Table_4

izab059_suppl_Supplementary_Table_5

izab059_suppl_Supplementary_Table_6

izab059_suppl_Supplementary_Table_7

izab059_suppl_Supplementary_Table_Legends

## Notes

Author Contribution: MI and SG contributed to the study conception and design. MI, SS, OMN, US, DZ, and RC contributed to data acquisition. MI, AA, LJ, OMN, AB, JW, GK, and SG contributed to the analysis and interpretation of data. AA and AB contributed to the biostatistical analysis. MI, OMN, LJ, AA, and SG drafted the article. MI, AA, LJ, OMN, SS, US, JW DZ, RC, GK, and SG critically revised the manuscript. MI, AA, LJ, OMN, SS,.US, JW, DZ, RC, GK, and SG gave final approval of the manuscript.

Supported by: MI and SG are funded by the NIHR Birmingham Biomedical Research Centre at the University Hospitals Birmingham NHS Foundation Trust and the University of Birmingham. AA was supported by the National Institute for Health Research (NIHR) Surgical Reconstruction and Microbiology Research Centre (SRMRC), Birmingham, UK. The views expressed are those of the author(s) and not necessarily those of the NHS, the NIHR or the Department of Health. Olympus has kindly loaned an endocytoscope for this study.

## Abbreviations

IBD: inflammatory bowel disease
UC: ulcerative colitis
CD: Crohn’s disease
MH: mucosal healing
MES: Mayo endoscopic score
HH: histological healing
ER: endoscopic remission
HR: histological remission
HD: WLE, high definition white light endoscopy
NBI: narrow banding imaging
ECSS: endocytoscope scoring system
UCEIS: ulcerative colitis endoscopic index score
SES-CD: simple endoscopic score for CD
PICaSSO: Paddington International virtual chromoendoscopy score
CDAI: Crohn’s disease activity index
HBI: Harvey–Bradshaw index
RHI: Robarts Histological Index
LP: lamina propria
GO: gene ontogeny
KEGG: kyoto encyclopedia of genes and genomes
AUC: area under curve
AUROC: area under receiver operating characteristic curve
PLS-DA: partial least squares discriminant analysis
PCA: principal component analysis
VIP: variable importance projection
VF: visual field
