# Supplementary material for: Ultra-high Magnification Endocytoscopy and Molecular Markers for Defining Endoscopic and Histologic Remission in Ulcerative Colitis—An Exploratory Study to Define Deep Remission
Source: Inflamm Bowel Dis. 2021 May 21;27(11):1719–30. doi: 10.1093/ibd/izab059 (PMC8528147; doi:10.1093/ibd/izab059)
Supplement: izab059_suppl_Supplementary_Table_1 [file izab059_suppl_supplementary_table_1.docx]

Supplementary file 1: Differentially expressed (Up and down) regulated genes in healed versus non-healed mucosa as defined by A) ECSS, B) Mayo, C)RHI and D) Nancy scores

A) ECCS Score

| Gene | logFC | CI.L | CI.R | AveExpr | t | P.Value | adj.P.Val | B |
| --- | --- | --- | --- | --- | --- | --- | --- | --- |
| RBM19 | 2.044353 | 1.116333 | 2.972373 | 0.883196 | 152.0693 | 4.10E-30 | 9.59E-28 | 57.21332 |
| QTRT2 | 2.500183 | 1.992547 | 3.007819 | 1.121663 | 119.7676 | 3.38E-28 | 6.73E-26 | 52.55863 |
| SVBP | 2.806107 | 2.510506 | 3.101708 | 1.640306 | 62.14957 | 6.12E-23 | 8.77E-21 | 39.7553 |
| USP2 | 2.933185 | 2.365593 | 3.500777 | 1.322209 | 59.38294 | 1.41E-22 | 1.97E-20 | 38.70751 |
| HECA | 2.187658 | 1.479372 | 2.895944 | 0.656094 | 48.5896 | 7.25E-21 | 9.13E-19 | 34.82907 |
| LACTB2 | 2.54801 | 0.978397 | 4.117623 | 1.073903 | 37.62444 | 6.13E-19 | 6.65E-17 | 31.56301 |
| RNF4 | 2.70509 | 2.192251 | 3.217928 | 0.907569 | 36.2566 | 1.21E-18 | 1.29E-16 | 31.51774 |
| CCZ1 | 2.553754 | 1.91243 | 3.195077 | 1.021038 | 32.39274 | 9.98E-18 | 1.02E-15 | 27.14156 |
| ZNF581 | 2.714869 | 2.049755 | 3.379983 | 0.979933 | 28.62194 | 8.90E-17 | 8.54E-15 | 26.93485 |
| CYP2B6 | 2.290757 | -0.69481 | 5.276326 | 1.161438 | 31.15583 | 1.91E-17 | 1.89E-15 | 26.32576 |
| TRNP | 3.733059 | 3.182252 | 4.283866 | 1.489721 | 30.18452 | 3.61E-17 | 3.52E-15 | 25.5099 |
| AIMP2 | 3.459043 | 2.206548 | 4.711538 | 0.868984 | 25.28981 | 8.32E-16 | 7.40E-14 | 25.44484 |
| PEPD | 3.364649 | 2.743272 | 3.986025 | 1.327562 | 27.76661 | 1.64E-16 | 1.52E-14 | 24.01114 |
| ATPAF1 | 4.063603 | 3.53749 | 4.589717 | 1.741297 | 27.67168 | 1.78E-16 | 1.64E-14 | 23.75224 |
| RCC1L | 2.085837 | 1.740788 | 2.430887 | 0.569284 | 23.43631 | 3.27E-15 | 2.76E-13 | 22.08107 |
| RMDN2 | 2.190249 | 1.552459 | 2.828039 | 0.861871 | 23.1537 | 4.75E-15 | 3.96E-13 | 20.46097 |
| TSNAX | 3.170193 | 2.551519 | 3.788867 | 1.487764 | 22.62958 | 6.47E-15 | 5.29E-13 | 20.14427 |
| UBFD1 | 3.078114 | 1.106412 | 5.049817 | 0.974677 | 17.59026 | 5.30E-13 | 3.93E-11 | 18.4169 |
| DHX8 | 2.13624 | 0.77594 | 3.49654 | 0.897775 | 20.4134 | 4.43E-14 | 3.47E-12 | 18.23961 |
| AKIRIN2 | 3.132657 | 2.559279 | 3.706035 | 1.235573 | 20.19476 | 5.23E-14 | 4.08E-12 | 17.90485 |
| SLC22A18AS | 2.427324 | 1.996222 | 2.858425 | 1.098352 | 19.3042 | 1.13E-13 | 8.64E-12 | 17.26576 |
| FSIP2-AS1 | 2.538045 | 2.231385 | 2.844705 | 0.812913 | 15.29682 | 5.99E-12 | 4.16E-10 | 16.61552 |
| ANGEL2 | 2.318862 | 1.746083 | 2.89164 | 0.85416 | 14.40591 | 1.68E-11 | 1.10E-09 | 15.28387 |
| MPP7 | 2.674475 | 1.078823 | 4.270127 | 1.174432 | 17.24219 | 8.45E-13 | 6.16E-11 | 14.97573 |
| ENPP4 | 2.191354 | 2.191322 | 2.191387 | 1.035684 | 16.62234 | 1.60E-12 | 1.16E-10 | 14.25335 |
| BIN3 | 3.529334 | 3.210426 | 3.848243 | 0.803558 | 16.33373 | 2.17E-12 | 1.55E-10 | 14.07418 |
| WEE1 | 2.079114 | 1.738722 | 2.419507 | 0.656271 | 16.3161 | 2.21E-12 | 1.57E-10 | 14.03614 |
| TRNI | 2.810699 | 1.838388 | 3.78301 | 1.220718 | 13.0676 | 8.73E-11 | 5.39E-09 | 12.70529 |
| TUSC2 | 2.963634 | 2.538834 | 3.388434 | 1.260674 | 15.14867 | 7.90E-12 | 5.46E-10 | 12.66635 |
| EEF1E1 | 3.249916 | 1.850422 | 4.649411 | 1.56335 | 15.01392 | 9.03E-12 | 6.18E-10 | 12.51874 |
| MED31 | 2.943466 | 2.56789 | 3.319043 | 1.213335 | 14.88753 | 1.06E-11 | 7.16E-10 | 12.40289 |
| C1orf35 | 3.700921 | 3.250473 | 4.151369 | 1.553506 | 14.69477 | 1.33E-11 | 8.76E-10 | 12.09741 |
| BTN3A1 | 2.060327 | 1.630121 | 2.490534 | 1.018597 | 14.50001 | 1.66E-11 | 1.09E-09 | 11.86975 |
| CPTP | 2.454969 | 2.008764 | 2.901173 | 0.937513 | 13.73866 | 4.14E-11 | 2.62E-09 | 10.88354 |
| FEM1C | 2.168002 | 1.33555 | 3.000455 | 1.233111 | 13.42129 | 6.04E-11 | 3.76E-09 | 10.54138 |
| ANKLE2 | 2.24581 | -1.03958 | 5.531202 | 1.040567 | 12.836 | 1.29E-10 | 7.90E-09 | 9.691726 |
| STAT6 | 2.104962 | 1.668676 | 2.541248 | 0.773144 | 9.597838 | 1.30E-08 | 6.64E-07 | 9.227968 |
| NTAN1 | 3.200848 | 2.515339 | 3.886357 | 1.616328 | 11.94191 | 4.23E-10 | 2.53E-08 | 8.550779 |
| DENND2A | 2.002039 | 1.533173 | 2.470906 | 0.70024 | 11.38297 | 9.28E-10 | 5.36E-08 | 7.906954 |
| MCU | 2.398353 | 1.307934 | 3.488772 | 1.195997 | 11.56166 | 7.20E-10 | 4.21E-08 | 7.902982 |
| RAB32 | 3.685852 | 3.114968 | 4.256736 | 1.265912 | 11.43687 | 8.59E-10 | 5.00E-08 | 7.760085 |
| GEMIN6 | 2.220184 | 1.312218 | 3.12815 | 1.346597 | 11.17728 | 1.24E-09 | 7.07E-08 | 7.392286 |
| POLR3GL | 3.730165 | 1.787087 | 5.673243 | 2.103339 | 10.97982 | 1.66E-09 | 9.38E-08 | 7.014975 |
| RNASET2 | 3.813432 | 2.687211 | 4.939652 | 2.67838 | 10.76361 | 2.28E-09 | 1.27E-07 | 6.548834 |
| BIN1 | 2.095973 | 0.710333 | 3.481613 | 0.93652 | 10.55876 | 3.10E-09 | 1.70E-07 | 6.398415 |
| SNAPIN | 3.663573 | 1.447871 | 5.879274 | 1.599302 | 10.58326 | 2.99E-09 | 1.65E-07 | 6.327282 |
| C6orf203 | 3.365172 | 2.820174 | 3.910171 | 1.559197 | 10.34479 | 4.29E-09 | 2.32E-07 | 6.018038 |
| C1orf123 | 3.465322 | 2.664821 | 4.265824 | 1.578687 | 10.24949 | 4.96E-09 | 2.66E-07 | 5.781661 |
| LY96 | 3.020784 | 2.18904 | 3.852528 | 1.393876 | 10.06976 | 6.56E-09 | 3.46E-07 | 5.69782 |
| SLC35B1 | 2.00445 | 0.527743 | 3.481156 | 0.990241 | 10.10326 | 6.22E-09 | 3.31E-07 | 5.658887 |
| PLRG1 | 2.942645 | 2.374229 | 3.511061 | 1.043451 | 7.789984 | 2.97E-07 | 1.31E-05 | 5.120305 |
| TNIP2 | 2.835446 | 2.075904 | 3.594989 | 1.259205 | 9.621056 | 1.33E-08 | 6.79E-07 | 5.02605 |
| AP1AR | 2.097359 | 2.097274 | 2.097443 | 0.98335 | 9.395108 | 1.92E-08 | 9.62E-07 | 4.48124 |
| INSIG1 | 2.721966 | 1.448222 | 3.995711 | 1.490891 | 9.344891 | 2.09E-08 | 1.04E-06 | 4.413448 |
| KIAA1522 | 2.085089 | 1.725738 | 2.44444 | 0.962693 | 9.284152 | 2.31E-08 | 1.15E-06 | 4.409443 |
| AKAP1 | 2.993598 | 2.563517 | 3.42368 | 1.436996 | 9.434641 | 1.80E-08 | 9.05E-07 | 4.399287 |
| LCN2 | -4.65975 | -6.48264 | -2.83687 | 4.924502 | -9.27705 | 2.34E-08 | 1.15E-06 | 4.209871 |
| RCN2 | 3.006828 | 2.404383 | 3.609273 | 1.41372 | 8.624381 | 7.03E-08 | 3.34E-06 | 3.080181 |
| ACAT1 | 2.812549 | 0.397801 | 5.227297 | 1.317663 | 8.526152 | 8.34E-08 | 3.92E-06 | 2.833017 |
| PPP2R3C | 3.379457 | 0.875809 | 5.883105 | 1.578451 | 8.427482 | 9.91E-08 | 4.62E-06 | 2.668195 |
| SHKBP1 | 2.903541 | 1.566629 | 4.240452 | 1.343222 | 8.308066 | 1.22E-07 | 5.64E-06 | 2.540237 |
| FNTA | 3.093005 | 2.272423 | 3.913587 | 1.543854 | 8.211334 | 1.45E-07 | 6.63E-06 | 2.260459 |
| TMEM171 | 3.307068 | 2.654412 | 3.959725 | 1.53924 | 7.915541 | 2.47E-07 | 1.10E-05 | 1.709427 |
| GLRX5 | 3.926558 | 3.349388 | 4.503728 | 1.926578 | 7.867183 | 2.70E-07 | 1.19E-05 | 1.653165 |
| TMEM56 | 2.216914 | 1.516645 | 2.917183 | 1.040859 | 7.7165 | 3.56E-07 | 1.56E-05 | 1.562078 |
| CSE1L | 2.353413 | 2.352159 | 2.354666 | 1.186302 | 7.69338 | 3.72E-07 | 1.62E-05 | 1.302135 |
| CRYL1 | 2.574171 | 0.584418 | 4.563924 | 1.402709 | 7.636687 | 4.13E-07 | 1.79E-05 | 1.250575 |
| SIRT6 | 2.342843 | 2.342823 | 2.342863 | 1.279651 | 7.299488 | 7.78E-07 | 3.25E-05 | 0.989804 |
| MMP24OS | 4.018274 | 3.703297 | 4.33325 | 1.962005 | 7.408871 | 6.33E-07 | 2.70E-05 | 0.677336 |
| CRYZL1 | 2.936727 | 2.356852 | 3.516602 | 1.443914 | 7.330937 | 7.33E-07 | 3.09E-05 | 0.656205 |
| RNASE6 | 2.02006 | 1.059716 | 2.980404 | 1.036319 | 7.343701 | 7.16E-07 | 3.02E-05 | 0.592286 |
| EDEM1 | 2.326945 | 1.098154 | 3.555736 | 1.147248 | 7.322101 | 7.46E-07 | 3.13E-05 | 0.509772 |
| PPID | 2.834726 | 2.486594 | 3.182857 | 1.417196 | 7.120982 | 1.10E-06 | 4.50E-05 | 0.160021 |
| PQBP1 | 2.545887 | 0.640088 | 4.451686 | 1.231564 | 7.071331 | 1.21E-06 | 4.93E-05 | 0.118905 |
| UMAD1 | 2.78878 | 2.788731 | 2.788829 | 1.077013 | 5.106448 | 6.79E-05 | 0.001996 | -0.2002 |
| TUFM | 2.842723 | 2.300926 | 3.38452 | 2.125172 | 6.92576 | 1.60E-06 | 6.37E-05 | -0.29034 |
| FAM104B | 2.039369 | 1.095508 | 2.98323 | 1.113283 | 6.803856 | 2.03E-06 | 8.04E-05 | -0.32903 |
| PPP1R35 | 3.956437 | 3.521764 | 4.391109 | 1.032592 | 4.803125 | 0.000133 | 0.003675 | -0.35351 |
| FLOT1 | 2.207133 | 1.933999 | 2.480268 | 1.210963 | 6.705476 | 2.47E-06 | 9.60E-05 | -0.499 |
| SDHAF3 | 2.403949 | 2.060778 | 2.747121 | 0.923721 | 6.723027 | 2.38E-06 | 9.35E-05 | -0.51366 |
| PCLAF | -3.16645 | -4.07282 | -2.26009 | 2.525913 | -6.71029 | 2.44E-06 | 9.54E-05 | -0.74725 |
| PTPRCAP | 2.911392 | 2.608748 | 3.214037 | 1.556755 | 6.639252 | 2.82E-06 | 0.000109 | -0.81622 |
| NR3C2 | 2.279403 | 1.506352 | 3.052453 | 1.179453 | 6.617856 | 2.94E-06 | 0.000113 | -0.89331 |
| GINS2 | 2.667458 | 2.179674 | 3.155242 | 1.13673 | 6.310668 | 5.46E-06 | 0.0002 | -1.3956 |
| HOXB9 | 2.072191 | 1.316101 | 2.82828 | 1.329053 | 6.307175 | 5.50E-06 | 0.000201 | -1.4846 |
| PDZK1IP1 | -4.49771 | -4.49785 | -4.49758 | 4.006909 | -6.32967 | 5.25E-06 | 0.000193 | -1.54133 |
| GTF3A | 3.74885 | 3.030238 | 4.467462 | 2.858464 | 6.299026 | 5.59E-06 | 0.000204 | -1.6372 |
| ALG3 | 2.436639 | 1.123307 | 3.74997 | 1.307249 | 5.991992 | 1.05E-05 | 0.000365 | -2.04125 |
| VSIG2 | 3.680443 | 2.230037 | 5.13085 | 2.007373 | 5.988658 | 1.06E-05 | 0.000366 | -2.28218 |
| NUDC | 2.372985 | 0.956802 | 3.789169 | 1.740297 | 5.8451 | 1.43E-05 | 0.000481 | -2.49247 |
| POGLUT1 | 2.230454 | 1.850632 | 2.610276 | 0.570013 | 3.735506 | 0.001456 | 0.033023 | -2.53831 |
| ASL | 3.140256 | 1.938284 | 4.342227 | 1.610077 | 5.708434 | 1.90E-05 | 0.000632 | -2.82054 |
| GCNT3 | -2.26342 | -3.24085 | -1.28599 | 3.67741 | -5.61656 | 2.31E-05 | 0.000751 | -3.004 |
| ACO2 | 2.113277 | 1.401618 | 2.824937 | 1.316509 | 5.604662 | 2.37E-05 | 0.000768 | -3.05205 |
| TGFBR2 | -2.3281 | -3.05997 | -1.59623 | 1.720101 | -5.59644 | 2.41E-05 | 0.000778 | -3.12285 |
| FZD5 | 2.176893 | 1.641974 | 2.711812 | 1.306024 | 5.544673 | 2.70E-05 | 0.000858 | -3.23433 |
| CCDC124 | 3.584463 | 3.065455 | 4.103471 | 2.037902 | 5.468355 | 3.17E-05 | 0.000998 | -3.38698 |
| VSIG10 | 2.081888 | 1.403044 | 2.760733 | 1.270378 | 5.418477 | 3.53E-05 | 0.001098 | -3.47837 |
| R3HCC1 | 2.035309 | 1.474976 | 2.595642 | 1.011615 | 5.167585 | 6.07E-05 | 0.00182 | -3.72733 |
| DUOX2 | -2.70628 | -2.7069 | -2.70566 | 2.121494 | -5.1522 | 6.28E-05 | 0.001873 | -4.07936 |
| COMMD4 | 2.956851 | 1.015696 | 4.898005 | 1.694066 | 5.141614 | 6.42E-05 | 0.001908 | -4.11925 |
| LSM14A | -2.20224 | -2.67968 | -1.7248 | 1.907003 | -5.12404 | 6.67E-05 | 0.00197 | -4.13684 |
| OXSR1 | 2.038853 | 1.363474 | 2.714231 | 1.161741 | 5.043004 | 7.96E-05 | 0.00232 | -4.25057 |
| CCT8 | 2.661845 | 0.483494 | 4.840197 | 1.472151 | 4.97226 | 9.30E-05 | 0.002668 | -4.41191 |
| DPM2 | 2.50036 | 2.169522 | 2.831198 | 1.319774 | 4.907122 | 0.000107 | 0.003045 | -4.47075 |
| TRPT1 | 2.815742 | 2.81308 | 2.818404 | 1.718767 | 4.959394 | 9.57E-05 | 0.002732 | -4.47434 |
| TXNL4A | 3.280097 | 0.232872 | 6.327323 | 1.959008 | 4.900434 | 0.000109 | 0.003077 | -4.65583 |
| TTC38 | 2.329782 | 1.671477 | 2.988087 | 1.566354 | 4.817851 | 0.000131 | 0.00363 | -4.86865 |
| PCK1 | 2.293387 | 1.681215 | 2.905559 | 1.497706 | 4.782835 | 0.000141 | 0.003889 | -4.92314 |
| ERI3 | 2.329428 | 1.637618 | 3.021238 | 1.506631 | 4.677703 | 0.000178 | 0.004828 | -5.10822 |
| TBCC | 2.3716 | 1.720187 | 3.023013 | 1.454201 | 4.632804 | 0.000197 | 0.005249 | -5.17766 |
| DAAM1 | -2.19259 | -3.74964 | -0.63554 | 1.599992 | -4.64212 | 0.000193 | 0.005162 | -5.24066 |
| CPEB4 | 2.335422 | -0.43306 | 5.103907 | 1.534287 | 4.6438 | 0.000192 | 0.005159 | -5.27049 |
| SCYL2 | 2.053542 | -0.96678 | 5.073859 | 1.227669 | 4.566437 | 0.000228 | 0.006047 | -5.34163 |
| PRDM1 | -2.06882 | -3.68521 | -0.45243 | 2.440991 | -4.58194 | 0.00022 | 0.005854 | -5.40429 |
| YIPF6 | 2.380613 | 1.886943 | 2.874282 | 1.470888 | 4.474145 | 0.00028 | 0.007326 | -5.61929 |
| B4GALT5 | 2.268608 | 1.789039 | 2.748177 | 1.297247 | 4.444259 | 0.000299 | 0.007786 | -5.67928 |
| ZMAT2 | -2.58039 | -4.05927 | -1.10152 | 2.412718 | -4.29361 | 0.000419 | 0.010636 | -6.05215 |
| TMEM208 | 3.433656 | 1.358988 | 5.508323 | 2.647198 | 4.159178 | 0.000567 | 0.014138 | -6.32329 |
| SNORA16A | -2.34988 | -3.09815 | -1.6016 | 2.065895 | -4.16176 | 0.000564 | 0.014083 | -6.3275 |
| PKP3 | 2.281578 | 1.513092 | 3.050065 | 1.657891 | 4.127334 | 0.000609 | 0.015018 | -6.43159 |
| NSD3 | -2.02584 | -2.50037 | -1.55132 | 2.017893 | -4.0894 | 0.000663 | 0.016204 | -6.47639 |
| PSMD3 | 2.222183 | 2.017898 | 2.426469 | 1.509329 | 3.97369 | 0.00086 | 0.020421 | -6.65177 |
| DHRSX_1 | 2.633795 | 1.965426 | 3.302164 | 1.658882 | 3.941504 | 0.000925 | 0.021684 | -6.8282 |
| FBLN1 | -2.15995 | -3.09809 | -1.22181 | 1.991384 | -3.94069 | 0.000927 | 0.021686 | -6.84792 |
| APOC1 | 2.428941 | 0.260529 | 4.597353 | 1.899563 | 3.641852 | 0.001814 | 0.040404 | -7.41234 |
| SMIM24 | 2.205442 | 1.61878 | 2.792104 | 1.195596 | 3.604961 | 0.001971 | 0.043601 | -7.46222 |
| AHR | -2.00219 | -2.69203 | -1.31236 | 2.240724 | -3.60397 | 0.001975 | 0.043625 | -7.58488 |
| PCCB | 2.290676 | 1.846339 | 2.735012 | 1.577295 | 3.569708 | 0.002133 | 0.046343 | -7.6349 |
| NUDCD2 | 2.118842 | 0.055209 | 4.182476 | 1.593866 | 3.550107 | 0.002229 | 0.048112 | -7.69202 |

B) Mayo Score

| Gene | logFC | CI.L | CI.R | AveExpr | t | P.Value | adj.P.Val | B |
| --- | --- | --- | --- | --- | --- | --- | --- | --- |
| IFI35 | 3.81213 | 2.709277 | 4.914983 | 1.35181 | 160.0525 | 5.20E-32 | 2.24E-29 | 63.68156 |
| IL6R | 2.38608 | 1.242511 | 3.529649 | 0.889591 | 154.5484 | 1.03E-31 | 4.29E-29 | 63.00309 |
| CCZ1 | 2.427393 | 2.426635 | 2.42815 | 0.972417 | 127.9454 | 4.16E-30 | 1.52E-27 | 59.25316 |
| SLC16A9 | 2.509788 | 1.16473 | 3.854846 | 1.114532 | 124.1351 | 7.52E-30 | 2.66E-27 | 58.64044 |
| CDS1 | 3.213184 | 2.762118 | 3.664249 | 1.303199 | 121.9818 | 1.06E-29 | 3.63E-27 | 58.33087 |
| NSUN2 | 2.316363 | 1.421229 | 3.211498 | 1.164852 | 115.4933 | 3.09E-29 | 9.72E-27 | 57.18276 |
| C6orf203 | 3.700584 | 2.719135 | 4.682032 | 1.48495 | 98.14872 | 7.45E-28 | 2.17E-25 | 53.75342 |
| C5orf51 | 3.097821 | 2.771519 | 3.424124 | 1.469766 | 82.80612 | 2.07E-26 | 5.60E-24 | 50.39084 |
| GINS2 | 3.58299 | 1.28423 | 5.881749 | 1.250605 | 78.06734 | 6.53E-26 | 1.69E-23 | 49.19201 |
| SMIM24 | 3.597224 | 3.25418 | 3.940267 | 1.309807 | 75.89623 | 1.13E-25 | 2.84E-23 | 49.09286 |
| PEPD | 3.744219 | 3.478287 | 4.010151 | 1.486938 | 75.84919 | 1.15E-25 | 2.84E-23 | 48.49344 |
| GMPPB | 2.89751 | 0.815772 | 4.979248 | 1.506204 | 68.76586 | 7.78E-25 | 1.78E-22 | 46.57878 |
| METTL7B | 3.550573 | 1.744268 | 5.356879 | 1.285729 | 68.62506 | 8.10E-25 | 1.81E-22 | 46.48247 |
| TRNP | 3.653698 | 3.243021 | 4.064375 | 1.418782 | 66.98538 | 1.30E-24 | 2.85E-22 | 46.08393 |
| SSRP1 | 2.24064 | 1.60227 | 2.87901 | 0.976933 | 60.94665 | 8.20E-24 | 1.77E-21 | 44.24517 |
| STX10 | 3.707534 | 2.429258 | 4.98581 | 1.334305 | 52.68165 | 1.40E-22 | 2.77E-20 | 41.97753 |
| EMC2 | 2.680362 | 0.618868 | 4.741857 | 1.000875 | 52.44487 | 1.53E-22 | 2.97E-20 | 40.98563 |
| EHHADH | 2.224983 | 2.154053 | 2.295913 | 0.748809 | 43.33901 | 6.67E-21 | 1.09E-18 | 37.0141 |
| KDM1A | 2.571702 | 2.197611 | 2.945794 | 1.003076 | 42.84543 | 7.80E-21 | 1.24E-18 | 36.93486 |
| TPSB2 | 3.851953 | 3.333078 | 4.370828 | 2.014786 | 43.20272 | 6.62E-21 | 1.09E-18 | 36.84314 |
| DCAF11 | 2.538222 | 2.047576 | 3.028869 | 0.999963 | 42.17972 | 1.12E-20 | 1.71E-18 | 36.4318 |
| UBE2D1 | 3.010271 | 2.538591 | 3.481951 | 1.454046 | 41.29398 | 1.59E-20 | 2.40E-18 | 36.20461 |
| RPA2 | 3.554161 | 1.766482 | 5.341841 | 1.694258 | 38.50748 | 6.15E-20 | 8.83E-18 | 34.69293 |
| UBP1 | 2.646686 | 2.646667 | 2.646705 | 1.262138 | 38.48611 | 6.22E-20 | 8.83E-18 | 34.67733 |
| AP1AR | 2.754229 | 2.135495 | 3.372963 | 1.083713 | 37.39509 | 1.16E-19 | 1.58E-17 | 33.97911 |
| ELAVL1 | 2.165178 | 1.70654 | 2.623816 | 0.761008 | 37.00949 | 1.41E-19 | 1.91E-17 | 33.84603 |
| CRYL1 | 3.60478 | 3.174428 | 4.035133 | 1.57977 | 35.40139 | 3.12E-19 | 4.14E-17 | 33.06312 |
| COMT | 2.785729 | 2.218543 | 3.352915 | 1.075839 | 34.74066 | 4.78E-19 | 6.18E-17 | 32.52022 |
| QTRT1 | 4.277155 | 2.656463 | 5.897847 | 2.147958 | 33.95247 | 7.36E-19 | 9.33E-17 | 31.86571 |
| UBAC1 | 3.34443 | 2.899612 | 3.789247 | 1.544884 | 30.71572 | 4.84E-18 | 5.76E-16 | 30.08277 |
| MGAT1 | 2.793909 | 2.215027 | 3.372792 | 1.551716 | 30.62158 | 5.11E-18 | 6.01E-16 | 29.96342 |
| RCOR1 | 2.197091 | 1.387772 | 3.006409 | 0.774117 | 30.57017 | 5.58E-18 | 6.50E-16 | 29.94961 |
| ISOC1 | 3.214549 | 2.73698 | 3.692118 | 1.745207 | 29.36287 | 1.14E-17 | 1.29E-15 | 29.20222 |
| GCHFR | 4.382337 | 2.860813 | 5.903861 | 1.929309 | 28.40045 | 2.24E-17 | 2.44E-15 | 28.54509 |
| PICALM | 2.508703 | 1.943153 | 3.074253 | 0.928804 | 25.13422 | 2.23E-16 | 2.22E-14 | 27.73191 |
| CPTP | 3.046462 | 2.343441 | 3.749483 | 1.069698 | 26.70145 | 7.41E-17 | 7.64E-15 | 27.10533 |
| NIPSNAP3A | 3.58658 | 1.766912 | 5.406249 | 1.480322 | 24.63434 | 3.43E-16 | 3.36E-14 | 25.58758 |
| HRCT1 | 3.390992 | 2.438035 | 4.343949 | 1.7973 | 24.35176 | 4.07E-16 | 3.95E-14 | 25.55902 |
| RMND5A | 2.109962 | 1.778944 | 2.44098 | 1.270568 | 24.29042 | 4.38E-16 | 4.22E-14 | 25.31225 |
| CYP2B6 | 2.313181 | 1.151238 | 3.475123 | 1.106131 | 24.11333 | 5.15E-16 | 4.91E-14 | 25.24244 |
| UGT2A3 | 2.775578 | 2.459482 | 3.091674 | 1.265528 | 24.03472 | 5.47E-16 | 5.19E-14 | 25.14209 |
| C1orf123 | 4.041846 | 3.674865 | 4.408826 | 1.503512 | 23.60209 | 7.72E-16 | 7.26E-14 | 24.57638 |
| KLHDC3 | 2.969751 | 2.121091 | 3.818411 | 1.176795 | 22.66835 | 1.66E-15 | 1.51E-13 | 23.95003 |
| EDIL3 | 3.109006 | 3.08937 | 3.128643 | 1.613949 | 22.69385 | 1.62E-15 | 1.50E-13 | 23.9122 |
| DHX8 | 2.119304 | 1.655522 | 2.583086 | 0.997975 | 22.48799 | 1.93E-15 | 1.74E-13 | 23.83181 |
| SNHG6 | 3.570333 | 2.390385 | 4.750281 | 1.916656 | 22.4463 | 1.99E-15 | 1.79E-13 | 23.72219 |
| MT1H | 4.828434 | 4.305371 | 5.351497 | 1.899374 | 21.93606 | 3.07E-15 | 2.67E-13 | 23.20686 |
| GON7 | 4.364283 | 2.607778 | 6.120787 | 2.211136 | 21.87368 | 3.24E-15 | 2.80E-13 | 23.11003 |
| KLHL24 | 2.280189 | 1.859959 | 2.700418 | 1.15928 | 21.68252 | 3.83E-15 | 3.28E-13 | 22.93861 |
| PPID | 3.278712 | 0.899326 | 5.658097 | 1.349711 | 21.4851 | 4.55E-15 | 3.87E-13 | 22.87312 |
| HOXB9 | 2.492461 | 1.65971 | 3.325212 | 1.265765 | 21.0622 | 6.60E-15 | 5.53E-13 | 22.50281 |
| PDE8A | 2.526607 | 1.529064 | 3.52415 | 1.011164 | 19.92914 | 1.86E-14 | 1.52E-12 | 21.25222 |
| POLR2G | 3.225841 | 2.592118 | 3.859564 | 1.748929 | 19.43002 | 2.99E-14 | 2.40E-12 | 21.01812 |
| METAP1 | 2.165615 | 0.756234 | 3.574995 | 0.796519 | 18.94324 | 4.79E-14 | 3.79E-12 | 20.49525 |
| ST20-MTHFS | 2.413194 | 2.044643 | 2.781744 | 1.444491 | 18.83517 | 5.32E-14 | 4.16E-12 | 20.34026 |
| ZNF703 | 2.849812 | 1.39277 | 4.306853 | 1.456374 | 18.73601 | 5.87E-14 | 4.56E-12 | 20.09334 |
| AXL | 2.234778 | 1.556791 | 2.912765 | 1.163575 | 17.92656 | 1.33E-13 | 1.01E-11 | 19.38932 |
| DCTN3 | 2.767126 | 2.188439 | 3.345814 | 1.437411 | 17.67059 | 1.74E-13 | 1.31E-11 | 19.00382 |
| ABCG2 | 2.272444 | 1.748544 | 2.796343 | 1.210788 | 17.01961 | 3.47E-13 | 2.56E-11 | 18.22351 |
| POLR2B | 2.521145 | 2.288938 | 2.753352 | 1.271944 | 16.8248 | 4.28E-13 | 3.12E-11 | 18.05307 |
| EGLN2 | 3.23813 | 2.819456 | 3.656805 | 1.434663 | 16.19795 | 8.58E-13 | 6.17E-11 | 17.32045 |
| GTF2B | 3.160028 | 2.535178 | 3.784878 | 1.140545 | 16.02395 | 1.04E-12 | 7.42E-11 | 17.11223 |
| TMEM87B | 3.115862 | 0.654439 | 5.577286 | 1.514928 | 15.73169 | 1.46E-12 | 1.03E-10 | 16.7399 |
| RMDN2 | 2.096451 | 1.65944 | 2.533462 | 0.820829 | 15.50005 | 1.91E-12 | 1.31E-10 | 16.43505 |
| APOC1 | 4.269745 | 1.43317 | 7.10632 | 1.809108 | 15.41195 | 2.12E-12 | 1.45E-10 | 16.39729 |
| GDPD3 | 3.380005 | 2.066475 | 4.693535 | 1.675791 | 14.85846 | 4.11E-12 | 2.77E-10 | 15.83581 |
| JUP | 2.659902 | 1.332486 | 3.987318 | 1.287695 | 14.52814 | 6.16E-12 | 4.10E-10 | 15.17961 |
| RNASET2 | 3.769172 | 3.17281 | 4.365535 | 2.778469 | 14.31608 | 8.03E-12 | 5.31E-10 | 14.85875 |
| TRNL2 | 3.249239 | 2.895854 | 3.602624 | 1.88589 | 14.11415 | 1.04E-11 | 6.66E-10 | 14.72313 |
| NUDC | 2.76272 | 2.187744 | 3.337696 | 1.686914 | 14.03616 | 1.14E-11 | 7.26E-10 | 14.70426 |
| HBB | 4.504794 | 3.55275 | 5.456838 | 1.80157 | 14.18375 | 9.48E-12 | 6.20E-10 | 14.70168 |
| PTPA | 2.101996 | 0.944142 | 3.259851 | 0.876405 | 14.03376 | 1.15E-11 | 7.26E-10 | 14.6194 |
| AKAP1 | 2.542423 | 1.699333 | 3.385513 | 1.368567 | 14.03888 | 1.14E-11 | 7.26E-10 | 14.61507 |
| DHRSX_1 | 2.962739 | 0.738356 | 5.187123 | 1.579888 | 13.8596 | 1.43E-11 | 9.02E-10 | 14.33733 |
| SLC22A18AS | 2.360073 | 2.358622 | 2.361525 | 1.19562 | 13.76647 | 1.62E-11 | 1.01E-09 | 14.33176 |
| GEMIN6 | 2.230654 | 1.970449 | 2.490859 | 1.282473 | 13.6158 | 1.96E-11 | 1.22E-09 | 14.15574 |
| RBM19 | 2.127396 | 2.127229 | 2.127563 | 0.841139 | 13.60894 | 1.98E-11 | 1.23E-09 | 14.13576 |
| TULP4 | 2.366759 | 2.156843 | 2.576674 | 1.383276 | 13.53519 | 2.18E-11 | 1.34E-09 | 13.82481 |
| ASL | 3.778875 | 1.90886 | 5.648891 | 1.758782 | 13.51108 | 2.25E-11 | 1.37E-09 | 13.77286 |
| FNTA | 3.149908 | 2.610374 | 3.689443 | 1.470337 | 13.36091 | 2.75E-11 | 1.67E-09 | 13.70339 |
| WDR82 | 2.156375 | 1.496326 | 2.816425 | 1.17754 | 13.28963 | 3.02E-11 | 1.80E-09 | 13.67529 |
| PTPRR | 2.503541 | 1.823214 | 3.183867 | 1.336919 | 13.02069 | 4.33E-11 | 2.54E-09 | 13.29222 |
| MIR200CHG | 4.351649 | 3.235042 | 5.468256 | 1.965887 | 12.62096 | 7.50E-11 | 4.30E-09 | 12.61469 |
| CDKN2B-AS1 | 2.279781 | 1.897688 | 2.661874 | 1.286727 | 12.4561 | 9.44E-11 | 5.31E-09 | 12.38239 |
| PDZK1IP1 | -4.9404 | -7.73611 | -2.14468 | 3.876659 | -12.4044 | 1.01E-10 | 5.66E-09 | 12.1684 |
| NCEH1 | 2.078717 | 0.753769 | 3.403665 | 1.098455 | 12.25654 | 1.25E-10 | 6.90E-09 | 12.11041 |
| DHX9 | 2.270945 | 1.857732 | 2.684158 | 1.187952 | 12.19581 | 1.36E-10 | 7.46E-09 | 12.014 |
| PCK1 | 2.97274 | 1.914884 | 4.030595 | 1.662883 | 12.17344 | 1.41E-10 | 7.63E-09 | 11.86426 |
| VPS4A | 3.383841 | 1.016586 | 5.751095 | 1.853375 | 12.18928 | 1.38E-10 | 7.49E-09 | 11.86176 |
| EPS8L3 | 2.70447 | 1.98811 | 3.42083 | 1.37344 | 12.02552 | 1.74E-10 | 9.34E-09 | 11.73268 |
| SAFB2 | 2.14293 | 1.251768 | 3.034093 | 0.874248 | 12.01532 | 1.77E-10 | 9.44E-09 | 11.70135 |
| SEC23B | 2.216806 | 1.346739 | 3.086874 | 1.306142 | 11.83936 | 2.28E-10 | 1.20E-08 | 11.53295 |
| NRDE2 | 2.03272 | 0.468988 | 3.596453 | 0.69734 | 11.72187 | 2.71E-10 | 1.41E-08 | 11.47314 |
| CHORDC1 | 2.132197 | 1.558722 | 2.705673 | 0.859779 | 11.62282 | 3.13E-10 | 1.61E-08 | 11.21632 |
| MSMO1 | 2.580988 | 2.039975 | 3.122002 | 1.346873 | 11.60938 | 3.20E-10 | 1.63E-08 | 11.19388 |
| ENDOD1 | 2.209804 | 1.489946 | 2.929663 | 1.099391 | 11.54878 | 3.50E-10 | 1.77E-08 | 10.9964 |
| CDK1 | 2.217946 | 0.834661 | 3.60123 | 1.204917 | 11.23049 | 5.64E-10 | 2.75E-08 | 10.42379 |
| DDT | 3.653811 | 2.022977 | 5.284644 | 3.649753 | 11.01818 | 7.80E-10 | 3.77E-08 | 10.11206 |
| KRTCAP3 | 4.382291 | 2.86385 | 5.900732 | 1.816872 | 10.9691 | 8.42E-10 | 4.05E-08 | 9.997693 |
| MCU | 2.267021 | 1.242524 | 3.291517 | 1.139044 | 10.83029 | 1.04E-09 | 4.99E-08 | 9.868723 |
| TBC1D15 | 2.010804 | -0.45177 | 4.473383 | 0.991343 | 10.38657 | 2.11E-09 | 9.98E-08 | 9.184823 |
| MEP1A | 2.971993 | 1.748593 | 4.195392 | 1.561482 | 10.33833 | 2.28E-09 | 1.07E-07 | 9.100863 |
| CAPN7 | 2.421791 | 1.180803 | 3.662779 | 1.218891 | 10.30808 | 2.39E-09 | 1.11E-07 | 9.010872 |
| NDUFS2 | 2.5687 | 1.887132 | 3.250268 | 1.561587 | 10.35622 | 2.21E-09 | 1.04E-07 | 8.977252 |
| TBCC | 2.595216 | 1.685796 | 3.504636 | 1.497952 | 9.938386 | 4.38E-09 | 1.98E-07 | 8.321841 |
| BIN1 | 2.063841 | 0.343497 | 3.784185 | 0.891924 | 9.866327 | 4.94E-09 | 2.16E-07 | 8.284155 |
| DPP7 | 2.502624 | 2.115685 | 2.889563 | 1.362659 | 9.894798 | 4.71E-09 | 2.09E-07 | 8.245976 |
| SMIM26 | 4.223594 | 3.466123 | 4.981065 | 3.92523 | 9.903227 | 4.64E-09 | 2.08E-07 | 8.225271 |
| DENR | 2.755666 | 2.462633 | 3.0487 | 1.407055 | 9.746094 | 6.04E-09 | 2.63E-07 | 8.001609 |
| TMEM209 | 2.112862 | 1.394212 | 2.831512 | 0.928052 | 9.516977 | 8.90E-09 | 3.87E-07 | 7.646688 |
| RBMX2 | 2.87727 | 0.655741 | 5.098798 | 1.601598 | 9.511563 | 8.98E-09 | 3.89E-07 | 7.577739 |
| TRIM25 | 2.100822 | 0.529186 | 3.672458 | 1.374375 | 9.467198 | 9.69E-09 | 4.17E-07 | 7.517458 |
| USF2 | 2.092815 | 0.962363 | 3.223268 | 1.357562 | 9.365801 | 1.15E-08 | 4.87E-07 | 7.407158 |
| FAM3B | 2.631898 | 1.914849 | 3.348947 | 1.281982 | 9.357987 | 1.17E-08 | 4.90E-07 | 7.396624 |
| VSIG10 | 2.025469 | 0.980631 | 3.070308 | 1.332005 | 9.44115 | 1.01E-08 | 4.32E-07 | 7.39645 |
| DNAJC2 | 2.849515 | 2.128011 | 3.571019 | 1.529434 | 9.35826 | 1.17E-08 | 4.90E-07 | 7.236794 |
| DUOX2 | -3.20995 | -4.27972 | -2.14017 | 2.020471 | -9.34469 | 1.20E-08 | 5.00E-07 | 7.212729 |
| SEMA5A | 2.259509 | 0.855222 | 3.663795 | 1.308777 | 8.952808 | 2.38E-08 | 9.35E-07 | 6.562141 |
| FAM120AOS | 2.804813 | 0.954221 | 4.655406 | 1.531234 | 8.897614 | 2.62E-08 | 1.02E-06 | 6.403628 |
| HEXB | 2.659623 | 2.081079 | 3.238167 | 1.621662 | 8.780592 | 3.23E-08 | 1.24E-06 | 6.222549 |
| ACY1 | 3.233039 | 3.162951 | 3.303127 | 1.887555 | 8.768603 | 3.30E-08 | 1.27E-06 | 6.190719 |
| NAA60 | 2.274401 | 1.891634 | 2.657168 | 0.909134 | 8.630444 | 4.24E-08 | 1.59E-06 | 5.988539 |
| WASL | 3.01237 | 1.775154 | 4.249586 | 1.908825 | 8.678902 | 3.88E-08 | 1.47E-06 | 5.979991 |
| GSTM4 | 2.095161 | 1.474303 | 2.71602 | 1.087826 | 8.568633 | 4.74E-08 | 1.76E-06 | 5.899534 |
| GGCT | 3.141801 | 2.738239 | 3.545364 | 1.850985 | 8.579498 | 4.65E-08 | 1.74E-06 | 5.890665 |
| DHRS11 | 3.463438 | 1.205335 | 5.721542 | 2.360587 | 8.575079 | 4.69E-08 | 1.75E-06 | 5.788515 |
| RHOF | 3.113268 | 1.830658 | 4.395878 | 1.899445 | 8.552337 | 4.89E-08 | 1.80E-06 | 5.787411 |
| ALDH6A1 | 2.421922 | 1.915792 | 2.928052 | 1.48238 | 8.512368 | 5.26E-08 | 1.92E-06 | 5.763965 |
| MRPL39 | 2.74205 | 0.646018 | 4.838083 | 2.304083 | 8.483816 | 5.54E-08 | 2.02E-06 | 5.639038 |
| RBM7 | 2.048493 | 2.033246 | 2.06374 | 1.21035 | 8.33107 | 7.34E-08 | 2.63E-06 | 5.526453 |
| ASNA1 | 3.120116 | 1.330311 | 4.909921 | 1.79675 | 8.356107 | 7.01E-08 | 2.52E-06 | 5.455621 |
| SQLE | 2.40892 | 1.625445 | 3.192395 | 1.187621 | 8.304944 | 7.71E-08 | 2.75E-06 | 5.352156 |
| CD14 | 3.228474 | 2.803788 | 3.65316 | 2.104191 | 8.22877 | 8.88E-08 | 3.13E-06 | 5.171648 |
| NAAA | 2.624839 | 1.818549 | 3.431128 | 1.753833 | 8.236114 | 8.76E-08 | 3.09E-06 | 5.125568 |
| GOT1 | 2.089117 | 2.089088 | 2.089147 | 1.075095 | 8.03142 | 1.29E-07 | 4.35E-06 | 4.933915 |
| IDH3G | 3.097601 | 2.616924 | 3.578279 | 1.714499 | 8.053938 | 1.23E-07 | 4.20E-06 | 4.872652 |
| TMEM170A | 2.506949 | 1.468569 | 3.545329 | 1.723101 | 8.103409 | 1.12E-07 | 3.85E-06 | 4.867786 |
| ALG2 | 2.45518 | 1.584282 | 3.326078 | 1.79052 | 8.043763 | 1.26E-07 | 4.26E-06 | 4.823251 |
| ETFDH | 2.45396 | -5.1E+10 | 5.05E+10 | 1.3846 | 8.028066 | 1.30E-07 | 4.36E-06 | 4.758491 |
| SDHAF3 | 2.645626 | 2.287163 | 3.004089 | 1.131932 | 7.910958 | 1.62E-07 | 5.39E-06 | 4.606017 |
| CBX1 | 2.353133 | 1.295522 | 3.410744 | 1.35995 | 7.884401 | 1.70E-07 | 5.66E-06 | 4.597429 |
| CTSA | 2.997917 | 2.545724 | 3.45011 | 1.682387 | 7.878723 | 1.72E-07 | 5.70E-06 | 4.50686 |
| WDR76 | 2.078544 | 1.543009 | 2.614079 | 1.118039 | 7.807443 | 1.97E-07 | 6.43E-06 | 4.476394 |
| AVPI1 | 2.490747 | 1.913512 | 3.067981 | 1.464245 | 7.839037 | 1.86E-07 | 6.08E-06 | 4.469332 |
| EMC10 | 2.801643 | 1.67193 | 3.931357 | 1.730104 | 7.84348 | 1.84E-07 | 6.05E-06 | 4.39603 |
| TMC4 | 2.23297 | 1.569498 | 2.896443 | 1.266361 | 7.664409 | 2.60E-07 | 8.33E-06 | 4.13207 |
| FRMD1 | 2.208894 | 0.60428 | 3.813509 | 1.461439 | 7.692992 | 2.46E-07 | 7.91E-06 | 4.103194 |
| TSNAX | 2.427658 | 2.111656 | 2.743661 | 1.536256 | 7.462122 | 3.87E-07 | 1.20E-05 | 3.701055 |
| GLIPR2 | 2.226456 | 1.255941 | 3.196971 | 1.558569 | 7.468906 | 3.82E-07 | 1.19E-05 | 3.684874 |
| CNDP2 | 2.151342 | 1.421078 | 2.881606 | 1.321477 | 7.480402 | 3.73E-07 | 1.16E-05 | 3.667321 |
| TMEM220 | 2.586355 | 0.983657 | 4.189052 | 1.386511 | 7.387453 | 4.48E-07 | 1.37E-05 | 3.505768 |
| DERA | 2.753326 | 1.460227 | 4.046425 | 2.536157 | 7.374307 | 4.60E-07 | 1.40E-05 | 3.44003 |
| SURF1 | 3.150912 | 2.649333 | 3.652492 | 2.841486 | 7.386158 | 4.49E-07 | 1.37E-05 | 3.42142 |
| AAMDC | 3.09191 | 2.841921 | 3.341899 | 1.516078 | 7.315322 | 5.17E-07 | 1.57E-05 | 3.343767 |
| VPS28 | 3.397973 | 3.134684 | 3.661261 | 2.916194 | 7.314033 | 5.19E-07 | 1.57E-05 | 3.301342 |
| TOR1AIP1 | 2.381197 | 0.964916 | 3.797478 | 1.405983 | 7.297388 | 5.36E-07 | 1.61E-05 | 3.267319 |
| OSBPL1A | 2.243912 | 0.074655 | 4.413169 | 1.159407 | 7.272305 | 5.64E-07 | 1.69E-05 | 3.199127 |
| TUFM | 2.647824 | 1.125266 | 4.170381 | 2.229522 | 7.24925 | 5.90E-07 | 1.76E-05 | 3.169104 |
| HSD17B2 | 3.554527 | 2.347388 | 4.761666 | 2.050393 | 7.203819 | 6.46E-07 | 1.91E-05 | 3.087958 |
| SLC38A1 | 2.026942 | 1.581235 | 2.472649 | 1.656473 | 7.189716 | 6.65E-07 | 1.96E-05 | 3.01441 |
| SAMD13 | 2.323792 | 0.452136 | 4.195447 | 1.187624 | 7.079699 | 8.30E-07 | 2.40E-05 | 2.951015 |
| CRK | 2.040108 | 0.783944 | 3.296272 | 1.253374 | 7.045106 | 8.90E-07 | 2.56E-05 | 2.826589 |
| NUDT19 | 2.267359 | 0.803483 | 3.731236 | 1.511936 | 7.065839 | 8.54E-07 | 2.46E-05 | 2.791979 |
| CUTA | 3.214536 | 2.763573 | 3.6655 | 3.317159 | 7.041614 | 8.96E-07 | 2.57E-05 | 2.727376 |
| ZHX1 | 2.011036 | 0.542515 | 3.479557 | 1.186531 | 6.911713 | 1.17E-06 | 3.30E-05 | 2.558568 |
| PCGF5 | 2.303893 | 0.156108 | 4.451678 | 1.425258 | 6.861663 | 1.29E-06 | 3.63E-05 | 2.338789 |
| RARRES3 | 2.811636 | 2.499595 | 3.123677 | 1.502137 | 6.747696 | 1.64E-06 | 4.51E-05 | 2.199945 |
| SNX2 | 2.038931 | 1.556562 | 2.5213 | 1.464372 | 6.67959 | 1.88E-06 | 5.13E-05 | 1.95447 |
| NOB1 | 2.400336 | 1.978632 | 2.82204 | 1.365484 | 6.629186 | 2.09E-06 | 5.60E-05 | 1.910452 |
| CREG1 | 2.353018 | -0.36597 | 5.072007 | 1.335665 | 6.632584 | 2.08E-06 | 5.58E-05 | 1.877562 |
| RGS10 | 3.095706 | 3.074386 | 3.117025 | 1.537646 | 6.568315 | 2.38E-06 | 6.33E-05 | 1.754538 |
| CNOT1 | 2.046916 | 1.222125 | 2.871707 | 1.418955 | 6.528415 | 2.58E-06 | 6.84E-05 | 1.603029 |
| AIP | 2.792925 | 1.806215 | 3.779636 | 1.142675 | 6.478214 | 2.87E-06 | 7.51E-05 | 1.599579 |
| LCN2 | -4.29177 | -4.80555 | -3.77799 | 4.752504 | -6.49879 | 2.75E-06 | 7.23E-05 | 1.588486 |
| PQBP1 | 2.658083 | 0.92163 | 4.394535 | 1.172918 | 6.429656 | 3.18E-06 | 8.28E-05 | 1.496309 |
| TLK1 | 2.263834 | 1.386696 | 3.140973 | 1.628956 | 6.425613 | 3.21E-06 | 8.34E-05 | 1.406737 |
| SMIM31 | 2.954233 | 1.107271 | 4.801194 | 2.023474 | 6.41416 | 3.28E-06 | 8.50E-05 | 1.395006 |
| MT1G | 4.546925 | 4.208851 | 4.884999 | 4.649754 | 6.419261 | 3.25E-06 | 8.43E-05 | 1.378188 |
| MT1F | 4.259469 | 3.753884 | 4.765054 | 2.208922 | 6.406498 | 3.34E-06 | 8.62E-05 | 1.369809 |
| REP15 | 3.033199 | 1.932429 | 4.133969 | 2.735606 | 6.396021 | 3.41E-06 | 8.78E-05 | 1.355553 |
| SCIN | 2.190109 | 1.099575 | 3.280644 | 1.52838 | 6.325107 | 3.96E-06 | 0.000101 | 1.25388 |
| PSMG2 | 3.159388 | 2.510438 | 3.808339 | 1.802924 | 6.311551 | 4.08E-06 | 0.000103 | 1.216825 |
| RAB4B | 2.198232 | 1.986056 | 2.410409 | 1.498087 | 6.262671 | 4.53E-06 | 0.000113 | 1.187994 |
| ANAPC5 | 2.243913 | 1.965057 | 2.52277 | 1.427675 | 6.267312 | 4.48E-06 | 0.000112 | 1.150458 |
| EPB41L4B | 2.019451 | 1.566974 | 2.471928 | 1.217863 | 6.275018 | 4.41E-06 | 0.00011 | 1.097075 |
| C1orf115 | 2.308287 | 1.743402 | 2.873173 | 1.454249 | 6.250897 | 4.64E-06 | 0.000115 | 1.022 |
| PABPN1 | 2.446337 | 1.967203 | 2.925471 | 1.89907 | 6.239065 | 4.76E-06 | 0.000117 | 0.966488 |
| USP2 | 2.105157 | -0.39798 | 4.608291 | 1.259246 | 6.179989 | 5.40E-06 | 0.000133 | 0.9304 |
| TRMT10C | 2.646964 | 2.468229 | 2.825698 | 1.645522 | 6.069186 | 6.85E-06 | 0.000166 | 0.742142 |
| GOLGA5 | 2.413181 | 1.974364 | 2.851998 | 1.198381 | 6.01531 | 7.70E-06 | 0.000186 | 0.541526 |
| FRA10AC1 | 2.49868 | 1.782121 | 3.215239 | 1.890328 | 5.970625 | 8.48E-06 | 0.000203 | 0.379245 |
| RAB11B | 3.112027 | 1.311563 | 4.912491 | 2.165634 | 5.92332 | 9.40E-06 | 0.000223 | 0.267539 |
| PLIN3 | 2.982609 | 2.267094 | 3.698124 | 2.473966 | 5.920072 | 9.46E-06 | 0.000224 | 0.259613 |
| SFT2D1 | 3.660535 | 3.530818 | 3.790253 | 3.292997 | 5.900918 | 9.86E-06 | 0.000232 | 0.240878 |
| B3GNT3 | 2.157871 | 1.770258 | 2.545484 | 1.351462 | 5.813051 | 1.19E-05 | 0.000277 | 0.113425 |
| TEX264 | 2.461619 | 2.016966 | 2.906272 | 1.330928 | 5.794122 | 1.25E-05 | 0.000286 | 0.07098 |
| TM2D3 | 2.000059 | -0.55653 | 4.556649 | 1.483152 | 5.683959 | 1.59E-05 | 0.000357 | -0.07691 |
| ERI3 | 2.335942 | 2.029368 | 2.642517 | 1.593841 | 5.691795 | 1.56E-05 | 0.000353 | -0.14751 |
| GRPEL1 | 2.992974 | 2.605364 | 3.380583 | 2.33467 | 5.724792 | 1.45E-05 | 0.000329 | -0.15827 |
| RBBP8 | 2.163771 | -0.50428 | 4.831827 | 1.064359 | 5.643565 | 1.73E-05 | 0.000388 | -0.1986 |
| NFKBIZ | -2.77804 | -3.4394 | -2.11668 | 1.507165 | -5.59643 | 1.92E-05 | 0.000426 | -0.44976 |
| CSNK2A1 | 2.246974 | 0.989489 | 3.504459 | 1.78731 | 5.591062 | 1.95E-05 | 0.00043 | -0.45779 |
| RAB10 | 2.067494 | -0.17113 | 4.306121 | 3.102669 | 5.567047 | 2.05E-05 | 0.000451 | -0.49479 |
| SCGB2A1 | 3.076714 | 1.68196 | 4.471468 | 1.76205 | 5.521866 | 2.27E-05 | 0.000496 | -0.52268 |
| ACP5 | 2.034824 | 1.105118 | 2.964531 | 1.225476 | 5.486241 | 2.46E-05 | 0.000532 | -0.63158 |
| DCTN6 | 2.721257 | 2.450373 | 2.99214 | 1.640891 | 5.460951 | 2.60E-05 | 0.000559 | -0.66584 |
| HSD11B2 | 2.453495 | 0.971747 | 3.935242 | 1.571458 | 5.482981 | 2.47E-05 | 0.000535 | -0.69212 |
| CAMLG | 2.318806 | 2.312485 | 2.325128 | 1.283769 | 5.36543 | 3.21E-05 | 0.000679 | -0.84466 |
| TCEAL8 | 2.84198 | 2.205847 | 3.478112 | 2.238952 | 5.403129 | 2.96E-05 | 0.000632 | -0.8674 |
| MICU1 | 2.542093 | 1.97413 | 3.110056 | 1.646492 | 5.381378 | 3.10E-05 | 0.000658 | -0.89163 |
| PPP1R2 | 2.412873 | 0.424197 | 4.40155 | 1.687844 | 5.374533 | 3.15E-05 | 0.000666 | -0.94141 |
| NUCB1 | 2.118127 | 2.118085 | 2.11817 | 1.407478 | 5.330652 | 3.47E-05 | 0.000727 | -0.98209 |
| ECHDC1 | 2.275178 | 1.759934 | 2.790422 | 1.559403 | 5.294962 | 3.76E-05 | 0.000783 | -1.08842 |
| EFNA1 | 2.781345 | 0.281601 | 5.281088 | 1.808627 | 5.2696 | 3.98E-05 | 0.000823 | -1.13756 |
| DUOXA2 | -3.08296 | -4.11024 | -2.05568 | 2.24527 | -5.24289 | 4.23E-05 | 0.000869 | -1.24219 |
| THYN1 | 3.035184 | 3.029653 | 3.040716 | 2.203594 | 5.189171 | 4.77E-05 | 0.000969 | -1.365 |
| GSN | -2.40557 | -3.28037 | -1.53078 | 2.453364 | -5.19478 | 4.71E-05 | 0.00096 | -1.37154 |
| FUCA2 | 2.571891 | 1.930309 | 3.213473 | 1.752289 | 5.180771 | 4.87E-05 | 0.000983 | -1.38019 |
| CUL3 | 2.016938 | 1.69653 | 2.337345 | 1.326686 | 5.168617 | 5.00E-05 | 0.001003 | -1.39137 |
| CCL5 | 3.20274 | 2.445547 | 3.959933 | 2.062241 | 5.118803 | 5.60E-05 | 0.001105 | -1.48415 |
| CCDC124 | 2.849704 | 1.689602 | 4.009805 | 1.940859 | 5.114499 | 5.65E-05 | 0.001114 | -1.48551 |
| ALDH3A2 | 2.417598 | 1.713577 | 3.12162 | 1.992443 | 5.077639 | 6.14E-05 | 0.001207 | -1.62127 |
| FIS1 | 3.177036 | 2.740012 | 3.614061 | 3.155817 | 5.022674 | 6.96E-05 | 0.001344 | -1.73776 |
| OAF | 2.800186 | 1.805476 | 3.794897 | 1.948356 | 5.012568 | 7.12E-05 | 0.001362 | -1.75026 |
| CFTR | 2.283704 | 1.826024 | 2.741384 | 2.035253 | 5.02991 | 6.84E-05 | 0.001324 | -1.75867 |
| LEO1 | 2.218259 | 1.422328 | 3.01419 | 1.727513 | 4.998242 | 7.35E-05 | 0.0014 | -1.77089 |
| ARCN1 | 2.445365 | 1.78792 | 3.10281 | 1.745105 | 4.936888 | 8.45E-05 | 0.001588 | -1.95514 |
| MISP | 2.824194 | 2.472112 | 3.176276 | 2.316248 | 4.939784 | 8.40E-05 | 0.001581 | -1.96879 |
| MBOAT1 | 2.06409 | 1.472302 | 2.655878 | 1.885916 | 4.922342 | 8.74E-05 | 0.001634 | -1.98321 |
| JUN | -2.30367 | -2.65096 | -1.95638 | 2.745356 | -4.86551 | 9.95E-05 | 0.001836 | -2.1264 |
| PDCD6 | 2.559205 | 1.539612 | 3.578799 | 2.831536 | 4.850862 | 0.000103 | 0.001884 | -2.16367 |
| SERINC3 | 2.764837 | 2.324077 | 3.205597 | 2.072232 | 4.841282 | 0.000105 | 0.001919 | -2.1984 |
| MT1M | 3.412285 | 2.436821 | 4.387749 | 2.22102 | 4.804034 | 0.000114 | 0.002067 | -2.22839 |
| HIST1H1C | 3.713998 | 3.38133 | 4.046665 | 2.886076 | 4.808676 | 0.000113 | 0.002052 | -2.22919 |
| TXNL4A | 2.587991 | 0.511485 | 4.664498 | 1.865722 | 4.791755 | 0.000118 | 0.002116 | -2.25635 |
| TFAM | 2.131513 | 1.655924 | 2.607103 | 1.417571 | 4.754157 | 0.000128 | 0.002288 | -2.33221 |
| MB | 2.033137 | -0.32049 | 4.386768 | 1.319524 | 4.633974 | 0.000169 | 0.002951 | -2.50301 |
| AK6 | 2.6649 | 1.965073 | 3.364728 | 2.826331 | 4.671661 | 0.000155 | 0.002719 | -2.51355 |
| LACTB | 2.078689 | 1.505678 | 2.6517 | 1.469511 | 4.613965 | 0.000177 | 0.003071 | -2.67449 |
| MTIF3 | 2.597731 | 1.964495 | 3.230966 | 2.085317 | 4.584171 | 0.000189 | 0.003265 | -2.78567 |
| CAT | 3.069179 | 2.604507 | 3.53385 | 2.678741 | 4.526631 | 0.000216 | 0.0037 | -2.92487 |
| UBXN1 | 2.522243 | 1.262767 | 3.78172 | 2.12293 | 4.4547 | 0.000255 | 0.004284 | -3.0429 |
| MKRN1 | 2.452401 | 1.515779 | 3.389022 | 1.992011 | 4.415428 | 0.000279 | 0.00465 | -3.17724 |
| CCL2 | 3.257504 | 3.190672 | 3.324335 | 2.504038 | 4.374086 | 0.000307 | 0.005072 | -3.22197 |
| LEPROTL1 | 2.231479 | 1.755336 | 2.707623 | 1.721721 | 4.385941 | 0.000299 | 0.00495 | -3.22449 |
| CLCN3 | 2.000289 | 1.029934 | 2.970643 | 1.760372 | 4.331861 | 0.000339 | 0.005556 | -3.33923 |
| DNAJC1 | 2.198136 | 1.508259 | 2.888012 | 1.926814 | 4.240776 | 0.000418 | 0.006666 | -3.56829 |
| AKR1B10 | 2.451956 | 1.434059 | 3.469852 | 2.434046 | 4.199418 | 0.00046 | 0.007236 | -3.66526 |
| MRPS34 | 3.165582 | 2.925414 | 3.40575 | 2.740135 | 4.193928 | 0.000466 | 0.007309 | -3.67071 |
| MRPL50 | 2.44726 | -0.0759 | 4.97042 | 1.994857 | 4.172634 | 0.00049 | 0.007647 | -3.7128 |
| NOSIP | 2.195432 | 1.050878 | 3.339986 | 1.741842 | 4.149011 | 0.000517 | 0.008045 | -3.74454 |
| POLR3GL | 2.536324 | 2.106239 | 2.966409 | 2.052716 | 4.10777 | 0.000569 | 0.008803 | -3.84602 |
| PPP2CB | 2.475692 | 1.98568 | 2.965704 | 1.846391 | 4.109277 | 0.000567 | 0.008784 | -3.85509 |
| PSMD8 | 2.696705 | 1.777912 | 3.615498 | 2.118568 | 4.104463 | 0.000573 | 0.008859 | -3.88969 |
| WASHC3 | 3.16387 | 2.418596 | 3.909144 | 2.557657 | 4.062413 | 0.000632 | 0.00967 | -3.94749 |
| MTHFS | 2.082718 | 0.620448 | 3.544988 | 1.637732 | 4.049919 | 0.00065 | 0.00992 | -3.97408 |
| CENPV | 2.516683 | 2.084395 | 2.94897 | 1.908838 | 4.021741 | 0.000694 | 0.010519 | -4.05766 |
| TRPT1 | 2.369377 | 1.896312 | 2.842442 | 1.636921 | 3.99311 | 0.000742 | 0.011082 | -4.07932 |
| RIOK3 | 2.570696 | 1.992926 | 3.148465 | 2.518952 | 4.010326 | 0.000713 | 0.01069 | -4.13334 |
| GINM1 | 2.707617 | 1.241943 | 4.173291 | 2.220697 | 3.97112 | 0.000781 | 0.011571 | -4.22069 |
| SDF4 | 2.242175 | 1.741515 | 2.742836 | 1.856597 | 3.900327 | 0.00092 | 0.013476 | -4.36125 |
| CS | 2.20779 | 2.046722 | 2.368859 | 1.735868 | 3.899202 | 0.000922 | 0.013476 | -4.38389 |
| PLD3 | 2.030092 | 0.976929 | 3.083255 | 2.214734 | 3.783553 | 0.001205 | 0.017178 | -4.62292 |
| PRKACB | 2.111075 | 1.622899 | 2.59925 | 2.128957 | 3.787886 | 0.001193 | 0.017049 | -4.63516 |
| LGALS2 | 3.609555 | 3.100026 | 4.119085 | 3.054542 | 3.777752 | 0.001221 | 0.017368 | -4.63576 |
| UBL3 | 2.220294 | 0.242522 | 4.198067 | 1.713003 | 3.746462 | 0.001312 | 0.0184 | -4.73793 |
| HADHB | 2.676563 | 2.19824 | 3.154886 | 2.443162 | 3.743767 | 0.001321 | 0.01846 | -4.75133 |
| DUSP23 | 2.368486 | 1.548847 | 3.188124 | 3.358994 | 3.717875 | 0.001402 | 0.019491 | -4.76453 |
| MPST | 2.729302 | 2.300547 | 3.158058 | 2.832505 | 3.701063 | 0.001457 | 0.020166 | -4.84484 |
| PIM2 | -2.37988 | -3.01189 | -1.74787 | 3.157232 | -3.67665 | 0.001542 | 0.021283 | -4.89979 |
| MT2A | 3.001102 | 0.813853 | 5.188352 | 4.313885 | 3.659577 | 0.001604 | 0.022033 | -4.92711 |
| CHP2 | 2.050242 | 1.125041 | 2.975443 | 2.493356 | 3.636102 | 0.001693 | 0.023041 | -4.97068 |
| NOP53 | 2.49899 | 0.657569 | 4.34041 | 3.311144 | 3.621248 | 0.001752 | 0.023788 | -5.01285 |
| DDRGK1 | 2.6094 | 2.257471 | 2.96133 | 2.25895 | 3.574733 | 0.00195 | 0.026051 | -5.10036 |
| SLC20A1 | 2.311949 | 1.751494 | 2.872405 | 1.956708 | 3.511036 | 0.002257 | 0.029816 | -5.26482 |
| TOMM7 | 2.210883 | -0.29474 | 4.716508 | 4.711357 | 3.467487 | 0.002494 | 0.03258 | -5.36075 |
| GUCA2A | 3.083389 | 2.292911 | 3.873867 | 3.884702 | 3.451519 | 0.002587 | 0.033606 | -5.41128 |
| SPTSSA | 2.308714 | 1.745421 | 2.872007 | 2.844345 | 3.427498 | 0.002734 | 0.03527 | -5.45168 |
| ARL8B | 2.456741 | 1.738536 | 3.174945 | 2.146121 | 3.423641 | 0.002758 | 0.035544 | -5.47525 |
| MSRB2 | 2.149326 | 0.493179 | 3.805472 | 1.9014 | 3.384664 | 0.003015 | 0.038517 | -5.50669 |
| UBE2I | 2.102909 | -0.34137 | 4.547187 | 2.36129 | 3.37004 | 0.003117 | 0.039695 | -5.57671 |
| MT1X | 2.099136 | 0.039158 | 4.159114 | 5.600482 | 3.371197 | 0.003109 | 0.039634 | -5.5817 |
| LTB | 2.716037 | 2.233168 | 3.198907 | 2.353622 | 3.322524 | 0.003474 | 0.043762 | -5.6509 |
| GRAMD2B | 2.050927 | 0.144836 | 3.957018 | 1.907566 | 3.333032 | 0.003392 | 0.042772 | -5.67031 |
| SCO2 | 2.39592 | 1.723721 | 3.068118 | 2.07891 | 3.3086 | 0.003586 | 0.045075 | -5.70868 |
| SEC13 | 2.703729 | 0.711021 | 4.696438 | 2.538779 | 3.279988 | 0.003828 | 0.047594 | -5.78664 |
| TST | 2.452017 | 1.84682 | 3.057213 | 3.721221 | 3.274126 | 0.003879 | 0.04813 | -5.79301 |
| CEBPG | 2.059055 | 0.866524 | 3.251586 | 2.161297 | 3.265415 | 0.003957 | 0.048937 | -5.81591 |
| TCEA3 | 2.734556 | 2.732428 | 2.736683 | 2.308541 | 3.266377 | 0.003948 | 0.048882 | -5.81946 |
| CXCL1 | -2.74943 | -3.28559 | -2.21326 | 2.841187 | -3.2589 | 0.004016 | 0.049614 | -5.83582 |

C) RHI Score

| Gene | logFC | CI.L | CI.R | AveExpr | t | P.Value | adj.P.Val | B |
| --- | --- | --- | --- | --- | --- | --- | --- | --- |
| COLGALT1 | 2.448437 | 1.287695 | 3.609179 | 0.854771 | 133.8343 | 3.56E-29 | 3.17E-26 | 56.83971 |
| AXL | 2.809427 | 1.900998 | 3.717855 | 1.04306 | 94.14676 | 2.42E-26 | 1.62E-23 | 50.05356 |
| CCZ1 | 2.662905 | 2.247912 | 3.077897 | 1.021038 | 89.46849 | 6.23E-26 | 3.78E-23 | 48.93296 |
| MCU | 2.633375 | 1.984741 | 3.282008 | 1.195997 | 81.42972 | 3.56E-25 | 2.07E-22 | 46.97422 |
| USP2 | 3.063964 | 2.282935 | 3.844992 | 1.322209 | 68.28226 | 9.27E-24 | 4.95E-21 | 43.79647 |
| CYP2B6 | 2.477012 | 1.433219 | 3.520806 | 1.161438 | 67.50043 | 1.15E-23 | 5.88E-21 | 43.57976 |
| CDK11B | 2.280687 | 1.387327 | 3.174047 | 1.269938 | 57.93331 | 1.93E-22 | 8.84E-20 | 40.25036 |
| SNORD89 | 3.288007 | 2.518838 | 4.057176 | 1.733113 | 57.84613 | 1.99E-22 | 8.84E-20 | 40.20446 |
| TSNAX | 3.262442 | 2.913089 | 3.611795 | 1.487764 | 37.35855 | 6.23E-19 | 2.25E-16 | 31.67218 |
| FNTA | 3.77068 | 3.119835 | 4.421526 | 1.543854 | 37.68046 | 5.32E-19 | 1.97E-16 | 31.65076 |
| ECHDC1 | 2.869572 | 2.438665 | 3.30048 | 1.421871 | 20.37056 | 3.70E-14 | 9.67E-12 | 19.95997 |
| TRNY | 3.747186 | 2.338096 | 5.156277 | 2.250054 | 17.37944 | 6.13E-13 | 1.49E-10 | 16.85812 |
| RMDN2 | 2.077343 | 1.641188 | 2.513499 | 0.861871 | 16.22082 | 2.05E-12 | 4.55E-10 | 15.68228 |
| TUSC2 | 2.997918 | 2.537783 | 3.458053 | 1.260674 | 15.69503 | 3.63E-12 | 7.80E-10 | 15.17195 |
| SLC52A2 | 3.179016 | 0.775827 | 5.582205 | 1.306519 | 15.22431 | 6.14E-12 | 1.26E-09 | 14.59268 |
| DCTN3 | 2.401108 | 2.093894 | 2.708321 | 1.317697 | 15.11622 | 6.94E-12 | 1.40E-09 | 14.37497 |
| NCEH1 | 2.091172 | 1.429724 | 2.75262 | 1.153378 | 14.16496 | 2.12E-11 | 3.92E-09 | 13.26254 |
| MED31 | 2.757586 | 2.101858 | 3.413315 | 1.213335 | 13.34268 | 5.85E-11 | 1.04E-08 | 12.19444 |
| UBP1 | 2.336538 | 0.752199 | 3.920877 | 1.096856 | 13.08268 | 8.15E-11 | 1.41E-08 | 11.9008 |
| ARRDC1 | 2.60054 | 1.756701 | 3.444379 | 1.342597 | 12.09071 | 3.04E-10 | 4.83E-08 | 10.47093 |
| ENPP4 | 2.202717 | 1.190456 | 3.214977 | 1.035684 | 12.11223 | 2.96E-10 | 4.75E-08 | 10.41172 |
| ACO2 | 2.499574 | 1.997658 | 3.00149 | 1.316509 | 11.14704 | 1.16E-09 | 1.73E-07 | 9.012808 |
| TRPT1 | 3.941203 | 3.601082 | 4.281323 | 1.718767 | 11.08127 | 1.27E-09 | 1.89E-07 | 8.814524 |
| PTPN18 | 2.884837 | 2.88476 | 2.884914 | 1.543403 | 10.70163 | 2.24E-09 | 3.24E-07 | 8.301359 |
| MRPS9 | 3.251539 | 2.105933 | 4.397146 | 1.723736 | 9.930204 | 7.37E-09 | 1.00E-06 | 7.017412 |
| P4HTM | 2.51378 | 2.027298 | 3.000261 | 1.305442 | 9.285492 | 2.10E-08 | 2.67E-06 | 6.068406 |
| RNASET2 | 3.706666 | 2.405789 | 5.007544 | 2.67838 | 9.242938 | 2.26E-08 | 2.81E-06 | 5.839428 |
| LUZP1 | 2.305325 | 1.462749 | 3.1479 | 1.268563 | 8.258746 | 1.24E-07 | 1.46E-05 | 4.037207 |
| GALE | 3.338605 | 2.56122 | 4.11599 | 1.562478 | 8.230661 | 1.30E-07 | 1.52E-05 | 4.026588 |
| CTCF | 2.192899 | 0.996964 | 3.388834 | 1.300226 | 7.651132 | 3.75E-07 | 4.04E-05 | 3.028586 |
| GSTM4 | 2.034871 | 1.585114 | 2.484629 | 1.142218 | 7.534297 | 4.68E-07 | 4.80E-05 | 2.748886 |
| STAT1 | -2.86201 | -4.80319 | -0.92083 | 2.103253 | -7.58761 | 4.23E-07 | 4.44E-05 | 2.713932 |
| RHOF | 2.979916 | 2.187616 | 3.772216 | 1.751 | 7.437187 | 5.62E-07 | 5.72E-05 | 2.478323 |
| RNPS1 | -2.6085 | -2.87046 | -2.34654 | 1.97136 | -7.40112 | 6.02E-07 | 6.08E-05 | 2.39208 |
| NUDT19 | 2.340475 | 2.340377 | 2.340572 | 1.547869 | 7.30119 | 7.28E-07 | 7.20E-05 | 2.214708 |
| CCDC28A | 2.70434 | 0.782514 | 4.626167 | 1.675085 | 7.25234 | 8.00E-07 | 7.85E-05 | 2.100491 |
| ASL | 2.74243 | 2.18036 | 3.3045 | 1.610077 | 7.152311 | 9.70E-07 | 9.38E-05 | 2.026058 |
| SNORD47 | -3.77433 | -5.04639 | -2.50227 | 2.689265 | -7.14624 | 9.82E-07 | 9.42E-05 | 1.851693 |
| MRPL1 | 2.49601 | 1.920505 | 3.071515 | 1.304657 | 7.099315 | 1.08E-06 | 0.0001 | 1.846544 |
| TMEM56 | 2.263489 | 1.769788 | 2.75719 | 1.040859 | 6.980714 | 1.36E-06 | 0.000126 | 1.666687 |
| SCYL2 | 2.024057 | 1.732167 | 2.315947 | 1.227669 | 6.912677 | 1.55E-06 | 0.000142 | 1.470867 |
| ERI3 | 2.532138 | 2.282368 | 2.781907 | 1.506631 | 6.765543 | 2.07E-06 | 0.000184 | 1.174109 |
| GATA6 | 2.25594 | 1.854613 | 2.657266 | 1.090087 | 6.608187 | 2.84E-06 | 0.000245 | 0.984401 |
| FAM104B | 2.135061 | 0.491856 | 3.778267 | 1.113283 | 6.444472 | 3.96E-06 | 0.000334 | 0.598621 |
| NUDT16 | 2.15245 | 1.837376 | 2.467525 | 1.123216 | 6.493513 | 3.58E-06 | 0.000304 | 0.590711 |
| DCTN6 | 2.711836 | 2.109516 | 3.314155 | 1.722936 | 6.293621 | 5.38E-06 | 0.000443 | 0.196032 |
| NUDC | 2.386658 | 1.838586 | 2.934731 | 1.740297 | 6.165839 | 7.01E-06 | 0.00056 | -0.06869 |
| PDZK1IP1 | -4.27711 | -4.70096 | -3.85325 | 4.006909 | -6.1693 | 6.96E-06 | 0.000559 | -0.14769 |
| ANAPC5 | 2.424776 | 1.698403 | 3.15115 | 1.499058 | 5.987883 | 1.01E-05 | 0.000774 | -0.47304 |
| WWP1 | -2.43861 | -2.90071 | -1.9765 | 2.078839 | -5.90628 | 1.20E-05 | 0.000907 | -0.72288 |
| HEPACAM2 | 2.758108 | 2.072547 | 3.443669 | 2.04585 | 5.848822 | 1.36E-05 | 0.001017 | -0.8705 |
| CASP5 | -3.13102 | -3.70178 | -2.56026 | 2.674777 | -5.6184 | 2.22E-05 | 0.001556 | -1.35102 |
| TUFM | 2.383816 | 2.224527 | 2.543105 | 2.125172 | 5.53015 | 2.68E-05 | 0.001841 | -1.49993 |
| C1orf210 | 2.614948 | 2.32017 | 2.909726 | 2.031779 | 5.524543 | 2.71E-05 | 0.001854 | -1.53121 |
| CPEB4 | 2.041106 | 1.809122 | 2.27309 | 1.534287 | 5.32803 | 4.14E-05 | 0.002736 | -2.00794 |
| AK2 | -2.23196 | -5.02828 | 0.564354 | 2.192713 | -5.25646 | 4.84E-05 | 0.003135 | -2.1686 |
| TGFBR2 | -2.20349 | -2.63462 | -1.77236 | 1.720101 | -5.20286 | 5.44E-05 | 0.003473 | -2.30211 |
| MRPL54 | 2.530108 | 1.870616 | 3.189599 | 2.903509 | 5.131535 | 6.36E-05 | 0.003983 | -2.39607 |
| GLRX5 | 2.963585 | 2.179231 | 3.747939 | 1.926578 | 5.054231 | 7.54E-05 | 0.004633 | -2.50828 |
| FIS1 | 2.992747 | 1.453327 | 4.532168 | 3.313608 | 5.01044 | 8.30E-05 | 0.005009 | -2.69166 |
| BZW2 | 2.047876 | -0.08112 | 4.176871 | 0.994719 | 4.776815 | 0.000139 | 0.008178 | -3.13516 |
| DHRSX_1 | 2.40107 | 0.08533 | 4.716809 | 1.658882 | 4.671179 | 0.000176 | 0.010124 | -3.45128 |
| NMI | -2.66251 | -3.26277 | -2.06226 | 2.120964 | -4.66221 | 0.00018 | 0.010241 | -3.5135 |
| ANP32A | 2.321131 | 1.91442 | 2.727843 | 2.993507 | 4.43022 | 0.000302 | 0.016309 | -4.04041 |
| SBDS | 2.193404 | 1.363331 | 3.023476 | 3.633575 | 4.392038 | 0.000329 | 0.017418 | -4.12096 |
| DYNC1LI2 | 2.7302 | 0.683578 | 4.776821 | 2.105871 | 4.403642 | 0.000321 | 0.017105 | -4.13022 |
| HNF4G | -2.00194 | -2.40374 | -1.60013 | 1.67976 | -4.30819 | 0.000397 | 0.020545 | -4.28997 |
| SLC25A1 | 2.568915 | 2.056027 | 3.081802 | 1.792055 | 4.280549 | 0.000423 | 0.021449 | -4.31281 |
| LXN | -2.77299 | -3.62682 | -1.91916 | 2.539336 | -4.28191 | 0.000422 | 0.021449 | -4.35401 |
| MGAT1 | 2.141514 | -0.32581 | 4.608837 | 1.487852 | 4.270835 | 0.000432 | 0.021841 | -4.37863 |
| DUOX2 | -2.41709 | -4.28455 | -0.54963 | 2.121494 | -4.25488 | 0.000448 | 0.02247 | -4.40015 |
| FERMT1 | 2.375085 | 1.979159 | 2.771011 | 2.145787 | 4.255541 | 0.000447 | 0.02247 | -4.43323 |
| UBXN1 | 2.441601 | 1.986914 | 2.896287 | 1.998288 | 4.208712 | 0.000497 | 0.024207 | -4.4799 |
| PLEKHJ1 | 3.032695 | 2.668536 | 3.396854 | 2.589318 | 4.173841 | 0.000538 | 0.025627 | -4.62688 |
| NUDCD2 | 2.210172 | 1.641529 | 2.778814 | 1.593866 | 4.112137 | 0.000618 | 0.028442 | -4.73626 |
| RNPEP | 2.494355 | 2.090464 | 2.898246 | 1.835929 | 4.036981 | 0.000733 | 0.033246 | -4.8742 |
| MMP24OS | 2.313257 | 2.022827 | 2.603686 | 1.962005 | 3.986317 | 0.000822 | 0.036533 | -4.95112 |
| POF1B | -2.36615 | -3.36627 | -1.36603 | 2.157271 | -3.98965 | 0.000815 | 0.03638 | -5.06196 |
| HERPUD1 | 2.304271 | 2.03267 | 2.575871 | 3.386884 | 3.957943 | 0.000876 | 0.038567 | -5.0805 |
| IFI6 | -2.05529 | -2.55977 | -1.55082 | 4.201512 | -3.9208 | 0.000953 | 0.04167 | -5.11411 |
| KRCC1 | 2.763519 | 2.20942 | 3.317619 | 2.255007 | 3.896246 | 0.001007 | 0.043761 | -5.24842 |
| RNF114 | 2.033581 | 1.301424 | 2.765739 | 1.503655 | 3.847728 | 0.001124 | 0.048032 | -5.32398 |
| NDUFB5 | -3.06875 | -6.31655 | 0.179039 | 3.649938 | -3.84488 | 0.001131 | 0.048053 | -5.38419 |

D) Nancy Score

| Gene | logFC | CI.L | CI.R | AveExpr | t | P.Value | adj.P.Val | B |
| --- | --- | --- | --- | --- | --- | --- | --- | --- |
| C7orf55-LUC7L2 | 2.973356 | 2.60268 | 3.344032 | 0.853482 | 973.1416 | 6.52E-45 | 3.48E-42 | 92.43679 |
| BTN2A1 | 2.589775 | 2.287399 | 2.892151 | 0.902246 | 433.8996 | 1.93E-38 | 3.67E-36 | 77.13244 |
| WEE1 | 2.320904 | 0.384721 | 4.257088 | 0.656271 | 398.4486 | 9.29E-38 | 1.55E-35 | 76.99243 |
| CCZ1 | 2.683669 | 0.415992 | 4.951345 | 1.021038 | 245.9757 | 6.79E-34 | 9.24E-32 | 66.21257 |
| MED31 | 3.315601 | 1.335997 | 5.295206 | 1.213335 | 242.3434 | 8.93E-34 | 1.20E-31 | 65.68843 |
| EDN3 | 2.888385 | 1.701879 | 4.07489 | 0.746363 | 205.131 | 1.93E-32 | 2.39E-30 | 64.46794 |
| SLC16A9 | 2.510903 | 1.832845 | 3.188962 | 1.170259 | 205.8292 | 1.81E-32 | 2.28E-30 | 62.26269 |
| USP2 | 3.063715 | 0.485283 | 5.642147 | 1.322209 | 170.4111 | 5.90E-31 | 6.40E-29 | 58.95116 |
| CPTP | 2.919831 | 1.717799 | 4.121863 | 0.937513 | 123.0577 | 2.38E-28 | 2.13E-26 | 53.81366 |
| RNF4 | 2.705159 | 1.970925 | 3.439394 | 0.907569 | 120.0257 | 3.77E-28 | 3.31E-26 | 53.44855 |
| TMEM97 | 3.306198 | 1.558131 | 5.054265 | 1.031917 | 95.05034 | 2.77E-26 | 2.11E-24 | 48.85804 |
| COLGALT1 | 2.462431 | 2.059514 | 2.865347 | 0.854771 | 91.66869 | 5.40E-26 | 4.03E-24 | 48.69441 |
| AIMP2 | 3.487156 | 2.736872 | 4.237441 | 0.868984 | 89.3522 | 8.66E-26 | 6.38E-24 | 48.47704 |
| CYP2B6 | 2.477085 | 1.237846 | 3.716324 | 1.161438 | 69.48567 | 8.84E-24 | 5.89E-22 | 42.48504 |
| CRACR2B | 2.282575 | 0.185604 | 4.379545 | 0.662691 | 57.30274 | 3.05E-22 | 1.84E-20 | 41.28492 |
| DNMBP | 2.326228 | -1.05072 | 5.703172 | 0.576117 | 65.63341 | 3.39E-23 | 2.22E-21 | 39.64474 |
| RMDN2 | 2.408938 | 1.434472 | 3.383405 | 0.861871 | 58.32415 | 2.92E-22 | 1.78E-20 | 37.247 |
| HOXB9 | 2.682496 | 2.00064 | 3.364352 | 1.329053 | 56.53259 | 3.91E-22 | 2.33E-20 | 36.96108 |
| EHHADH | 2.223402 | 1.567511 | 2.879294 | 0.763597 | 53.99182 | 1.19E-21 | 6.91E-20 | 35.89604 |
| MAFG | 2.228223 | 1.819736 | 2.63671 | 0.598625 | 42.24393 | 8.11E-20 | 4.22E-18 | 34.29909 |
| MCU | 2.729448 | 1.482053 | 3.976844 | 1.195997 | 48.02243 | 8.31E-21 | 4.56E-19 | 33.70988 |
| TRIP10 | 3.009567 | 3.009267 | 3.009867 | 1.262253 | 40.38897 | 1.84E-19 | 9.48E-18 | 32.5676 |
| C1orf174 | 2.29682 | 1.319431 | 3.27421 | 1.01741 | 36.53863 | 1.14E-18 | 5.59E-17 | 31.02043 |
| TRNP | 3.790563 | 2.02831 | 5.552816 | 1.489721 | 39.28826 | 3.36E-19 | 1.70E-17 | 29.80364 |
| KNOP1 | 3.555052 | 0.229917 | 6.880186 | 1.075287 | 29.31891 | 6.21E-17 | 2.87E-15 | 27.2852 |
| POLR1C | 2.41116 | 1.815291 | 3.007028 | 0.981427 | 27.17174 | 2.45E-16 | 1.09E-14 | 25.83447 |
| TCEANC2 | 2.114084 | 1.536106 | 2.692061 | 0.640683 | 30.3227 | 4.12E-17 | 1.91E-15 | 24.8894 |
| YOD1 | 2.251726 | 1.40002 | 3.103432 | 0.630349 | 22.08382 | 1.01E-14 | 4.16E-13 | 23.72576 |
| FNTA | 3.8008 | 3.188863 | 4.412737 | 1.543854 | 27.94975 | 1.69E-16 | 7.67E-15 | 23.2208 |
| TSNAX | 3.213507 | 0.379325 | 6.047689 | 1.487764 | 27.66631 | 1.96E-16 | 8.83E-15 | 23.16127 |
| RMI1 | 2.092177 | 0.053513 | 4.130841 | 0.809893 | 22.23836 | 8.90E-15 | 3.70E-13 | 22.99896 |
| SLC35B1 | 2.732088 | 1.038645 | 4.425532 | 0.990241 | 22.54165 | 6.99E-15 | 2.93E-13 | 22.59159 |
| KIAA1191 | 2.777883 | 1.416633 | 4.139133 | 1.296049 | 23.14282 | 4.37E-15 | 1.86E-13 | 22.38208 |
| CARNMT1 | 2.395839 | 1.601053 | 3.190626 | 0.882447 | 24.6258 | 1.71E-15 | 7.49E-14 | 20.8106 |
| PEPD | 3.375827 | 2.716792 | 4.034861 | 1.327562 | 22.5639 | 7.96E-15 | 3.33E-13 | 19.25178 |
| RCOR1 | 2.207431 | 0.766057 | 3.648805 | 0.695448 | 22.06852 | 1.19E-14 | 4.90E-13 | 18.87775 |
| ESCO1 | 2.410864 | 1.589855 | 3.231874 | 0.989235 | 21.22402 | 2.38E-14 | 9.62E-13 | 18.12087 |
| SLC22A18AS | 2.451748 | 2.451727 | 2.45177 | 1.098352 | 21.03564 | 2.75E-14 | 1.11E-12 | 18.09003 |
| ANGEL2 | 2.320377 | 1.747174 | 2.893581 | 0.85416 | 16.94474 | 1.07E-12 | 3.95E-11 | 18.08162 |
| DHX8 | 2.126274 | 0.458546 | 3.794001 | 0.897775 | 21.13826 | 2.55E-14 | 1.03E-12 | 18.06963 |
| NRARP | 2.43004 | 1.962687 | 2.897392 | 1.068269 | 17.46356 | 6.33E-13 | 2.35E-11 | 17.72656 |
| AXL | 2.237418 | 2.026419 | 2.448417 | 1.04306 | 20.58388 | 4.08E-14 | 1.63E-12 | 17.58648 |
| RNASET2 | 4.249637 | 3.913789 | 4.585486 | 2.67838 | 20.56747 | 4.14E-14 | 1.65E-12 | 17.31053 |
| PDZK1IP1 | -5.60813 | -6.03222 | -5.18403 | 4.006909 | -20.0512 | 6.47E-14 | 2.54E-12 | 16.86215 |
| SLC52A2 | 3.270025 | 0.55141 | 5.988641 | 1.306519 | 19.79658 | 8.08E-14 | 3.14E-12 | 16.84347 |
| TWSG1 | 2.486368 | 0.923375 | 4.049361 | 1.029715 | 15.76793 | 3.72E-12 | 1.30E-10 | 16.11874 |
| SURF2 | 2.783291 | 2.276591 | 3.289991 | 0.868903 | 14.25796 | 2.09E-11 | 7.06E-10 | 15.22762 |
| CDK11B | 2.228709 | 0.945362 | 3.512057 | 1.269938 | 17.82835 | 4.94E-13 | 1.86E-11 | 14.89304 |
| TUSC2 | 2.999059 | 2.989158 | 3.00896 | 1.260674 | 16.68282 | 1.59E-12 | 5.78E-11 | 13.66459 |
| ELAVL1 | 2.187195 | 1.595485 | 2.778905 | 0.799058 | 16.48699 | 1.95E-12 | 7.02E-11 | 13.41318 |
| SSRP1 | 2.41651 | 2.112921 | 2.720099 | 1.02578 | 16.27372 | 2.44E-12 | 8.74E-11 | 13.21645 |
| KLHDC2 | 2.078547 | 1.39049 | 2.766605 | 1.009655 | 15.91742 | 3.56E-12 | 1.25E-10 | 13.0265 |
| ENPP4 | 2.179243 | -0.10535 | 4.46383 | 1.035684 | 16.15862 | 2.75E-12 | 9.84E-11 | 13.00227 |
| SLC27A2 | 2.976645 | 2.03798 | 3.915309 | 1.290051 | 15.94305 | 3.47E-12 | 1.22E-10 | 12.90597 |
| SCML1 | 2.151266 | 1.948615 | 2.353916 | 0.890799 | 15.94217 | 3.47E-12 | 1.22E-10 | 12.76882 |
| EMC2 | 2.457522 | 2.153252 | 2.761793 | 1.028416 | 15.2968 | 7.03E-12 | 2.45E-10 | 12.07289 |
| BTN3A1 | 2.078392 | 1.233696 | 2.923088 | 1.018597 | 15.25522 | 7.37E-12 | 2.55E-10 | 12.04292 |
| RBM19 | 2.147287 | 0.323798 | 3.970777 | 0.883196 | 14.68492 | 1.41E-11 | 4.82E-10 | 11.4609 |
| TOMM40 | 3.174469 | 2.811968 | 3.536971 | 1.44834 | 11.51221 | 7.37E-10 | 2.23E-08 | 10.39573 |
| UBE2D1 | 2.816295 | 2.594203 | 3.038387 | 1.314221 | 13.8573 | 3.75E-11 | 1.24E-09 | 10.32199 |
| PPP1R35 | 4.043265 | 1.469199 | 6.617332 | 1.032592 | 10.76411 | 2.18E-09 | 6.25E-08 | 10.2874 |
| C1orf35 | 3.660725 | 3.408885 | 3.912565 | 1.553506 | 13.80063 | 4.01E-11 | 1.32E-09 | 10.20995 |
| PSMF1 | 2.115044 | 1.612514 | 2.617574 | 1.100495 | 13.81859 | 3.93E-11 | 1.29E-09 | 10.14865 |
| MGAT4B | 2.096633 | 1.643736 | 2.54953 | 0.818526 | 13.68205 | 4.64E-11 | 1.51E-09 | 10.06462 |
| POGLUT1 | 2.248572 | 0.981399 | 3.515744 | 0.570013 | 11.97195 | 3.88E-10 | 1.21E-08 | 10.05983 |
| CAPN7 | 2.341091 | 2.061652 | 2.62053 | 1.091799 | 13.33016 | 7.17E-11 | 2.32E-09 | 9.792124 |
| RAB32 | 3.785741 | 3.309704 | 4.261778 | 1.265912 | 12.97195 | 1.13E-10 | 3.63E-09 | 9.221832 |
| NTAN1 | 3.185764 | 1.823052 | 4.548477 | 1.616328 | 12.78952 | 1.43E-10 | 4.54E-09 | 9.016292 |
| ANKLE2 | 2.235264 | 1.822963 | 2.647566 | 1.040567 | 12.37396 | 2.46E-10 | 7.77E-09 | 8.332503 |
| SNHG25 | 3.301412 | 2.68459 | 3.918234 | 1.4569 | 10.05838 | 6.41E-09 | 1.74E-07 | 8.298405 |
| C5orf51 | 2.530155 | 1.277495 | 3.782815 | 1.35589 | 11.94619 | 4.39E-10 | 1.35E-08 | 7.679155 |
| THAP1 | 2.074863 | 0.986198 | 3.163527 | 1.114663 | 11.70405 | 6.12E-10 | 1.87E-08 | 7.428032 |
| EPS8L3 | 2.850031 | 2.253827 | 3.446235 | 1.279373 | 11.72497 | 5.95E-10 | 1.83E-08 | 7.353802 |
| DUOX2 | -3.33027 | -3.68371 | -2.97684 | 2.121494 | -11.609 | 6.99E-10 | 2.13E-08 | 7.080796 |
| UBFD1 | 2.689606 | 2.689558 | 2.689653 | 0.974677 | 8.842133 | 4.68E-08 | 1.16E-06 | 7.078165 |
| PDE8A | 2.271803 | 0.97669 | 3.566915 | 0.896731 | 11.16351 | 1.32E-09 | 3.87E-08 | 6.535539 |
| GTF2B | 2.912327 | 2.912247 | 2.912406 | 1.197572 | 10.82742 | 2.15E-09 | 6.24E-08 | 6.06125 |
| NCEH1 | 2.002233 | 1.339632 | 2.664834 | 1.153378 | 10.81695 | 2.18E-09 | 6.25E-08 | 5.979975 |
| GLRX5 | 4.280981 | 3.75816 | 4.803802 | 1.926578 | 10.82551 | 2.15E-09 | 6.24E-08 | 5.963599 |
| UGT2A3 | 2.532018 | 2.078919 | 2.985116 | 1.145835 | 10.68313 | 2.66E-09 | 7.52E-08 | 5.838591 |
| UBP1 | 2.285544 | -0.15752 | 4.728604 | 1.096856 | 10.55124 | 3.24E-09 | 9.00E-08 | 5.64475 |
| C1orf123 | 3.495482 | 2.708355 | 4.282609 | 1.578687 | 10.60944 | 2.97E-09 | 8.34E-08 | 5.636488 |
| VSIG10 | 2.364122 | 1.981099 | 2.747145 | 1.270378 | 10.58769 | 3.07E-09 | 8.59E-08 | 5.583776 |
| CNNM4 | 2.097288 | 2.097254 | 2.097322 | 0.91612 | 10.53647 | 3.31E-09 | 9.18E-08 | 5.536932 |
| DHRSX_1 | 3.258274 | 2.775611 | 3.740937 | 1.658882 | 10.57878 | 3.11E-09 | 8.69E-08 | 5.50189 |
| SNX14 | 2.1982 | 1.257286 | 3.139114 | 1.103197 | 10.38995 | 4.13E-09 | 1.14E-07 | 5.395501 |
| BIN1 | 2.114224 | 1.494234 | 2.734214 | 0.93652 | 10.37461 | 4.23E-09 | 1.16E-07 | 5.368496 |
| RCN2 | 3.096913 | 1.704593 | 4.489234 | 1.41372 | 10.22543 | 5.31E-09 | 1.45E-07 | 5.105555 |
| AP1AR | 2.120758 | 1.025418 | 3.216097 | 0.98335 | 9.952441 | 8.12E-09 | 2.17E-07 | 4.648369 |
| NELFCD | 2.771497 | 0.69712 | 4.845875 | 1.232264 | 9.937995 | 8.30E-09 | 2.22E-07 | 4.594104 |
| MIR22HG | 2.913346 | 2.06404 | 3.762652 | 0.868125 | 7.055286 | 1.22E-06 | 2.52E-05 | 4.517802 |
| C6orf203 | 3.154998 | 2.338211 | 3.971785 | 1.559197 | 9.724124 | 1.16E-08 | 3.08E-07 | 4.247387 |
| AKAP1 | 2.996021 | 2.491645 | 3.500397 | 1.436996 | 9.739193 | 1.14E-08 | 3.01E-07 | 4.201744 |
| POLR3GL | 3.526519 | 3.184796 | 3.868242 | 2.103339 | 9.482831 | 1.72E-08 | 4.49E-07 | 3.859821 |
| TMEM209 | 2.103622 | -0.15381 | 4.361055 | 0.974455 | 9.355783 | 2.11E-08 | 5.46E-07 | 3.633699 |
| ASL | 3.588122 | 3.205127 | 3.971117 | 1.610077 | 9.253373 | 2.50E-08 | 6.40E-07 | 3.359054 |
| LCN2 | -4.8817 | -6.88305 | -2.88035 | 4.924502 | -9.20957 | 2.68E-08 | 6.83E-07 | 3.346671 |
| GSTM4 | 2.119042 | 1.85711 | 2.380974 | 1.142218 | 9.069587 | 3.39E-08 | 8.56E-07 | 3.14862 |
| OXSR1 | 2.53886 | 0.29241 | 4.785311 | 1.161741 | 8.891075 | 4.57E-08 | 1.14E-06 | 2.741917 |
| ISOC1 | 2.743681 | 1.984805 | 3.502558 | 1.601496 | 8.764776 | 5.66E-08 | 1.39E-06 | 2.687141 |
| ZFYVE21 | 2.97238 | 2.971923 | 2.972838 | 1.317557 | 8.694322 | 6.39E-08 | 1.55E-06 | 2.433465 |
| TMEM56 | 2.407083 | 1.545139 | 3.269028 | 1.040859 | 8.412209 | 1.04E-07 | 2.48E-06 | 2.093342 |
| HSPA14 | 2.585395 | 1.539956 | 3.630835 | 0.853805 | 5.803582 | 1.53E-05 | 0.000273 | 2.062648 |
| CRYL1 | 2.642929 | 2.017582 | 3.268277 | 1.402709 | 8.447708 | 9.79E-08 | 2.35E-06 | 2.000301 |
| GOT1 | 2.099817 | 0.34234 | 3.857295 | 1.12885 | 8.347945 | 1.17E-07 | 2.76E-06 | 1.964918 |
| TNIP2 | 2.721967 | 1.340159 | 4.103776 | 1.259205 | 8.257663 | 1.37E-07 | 3.20E-06 | 1.880354 |
| TMEM171 | 3.36368 | 3.363421 | 3.363939 | 1.53924 | 8.387995 | 1.09E-07 | 2.59E-06 | 1.877229 |
| ITGB1BP1 | 2.074926 | 0.64479 | 3.505063 | 1.100548 | 8.164326 | 1.61E-07 | 3.71E-06 | 1.779527 |
| TUFM | 3.034215 | 1.739648 | 4.328781 | 2.125172 | 8.223816 | 1.45E-07 | 3.38E-06 | 1.487497 |
| PCK1 | 2.605471 | -0.80546 | 6.016405 | 1.497706 | 8.166244 | 1.61E-07 | 3.70E-06 | 1.372002 |
| ACAT1 | 2.719574 | 2.100674 | 3.338474 | 1.317663 | 8.035478 | 2.03E-07 | 4.64E-06 | 1.232942 |
| ZNF703 | 2.436165 | 1.513695 | 3.358636 | 1.301567 | 7.901053 | 2.59E-07 | 5.85E-06 | 0.999204 |
| DCAF6 | 2.070073 | 1.483048 | 2.657099 | 0.599172 | 5.075775 | 7.33E-05 | 0.001185 | 0.935852 |
| CSE1L | 2.34291 | 1.375322 | 3.310499 | 1.186302 | 7.832599 | 2.94E-07 | 6.59E-06 | 0.869885 |
| RSBN1L | 2.424064 | 2.104211 | 2.743917 | 1.320355 | 7.82726 | 2.97E-07 | 6.64E-06 | 0.824219 |
| DUSP3 | 2.064711 | 1.475769 | 2.653653 | 0.862563 | 7.592072 | 4.58E-07 | 1.00E-05 | 0.713651 |
| RNF11 | 2.332395 | 1.667296 | 2.997495 | 1.062682 | 7.528117 | 5.16E-07 | 1.12E-05 | 0.492373 |
| TRPT1 | 3.647179 | 1.701423 | 5.592936 | 1.718767 | 7.640164 | 4.19E-07 | 9.26E-06 | 0.401975 |
| CDK1 | 2.112382 | 1.884341 | 2.340423 | 1.239469 | 7.529813 | 5.14E-07 | 1.12E-05 | 0.20739 |
| SULT1A1 | 2.016674 | 0.362954 | 3.670394 | 0.816475 | 4.890616 | 0.00011 | 0.001731 | 0.189336 |
| P4HTM | 2.408499 | 1.148882 | 3.668116 | 1.305442 | 7.381554 | 6.79E-07 | 1.46E-05 | 0.065232 |
| ACTR1B | 2.32397 | 0.692559 | 3.955382 | 0.791875 | 7.361093 | 7.06E-07 | 1.51E-05 | 0.062269 |
| PPID | 2.876656 | 2.473251 | 3.280061 | 1.417196 | 7.395204 | 6.62E-07 | 1.43E-05 | -0.00659 |
| CEP70 | 2.110711 | 0.634833 | 3.58659 | 1.176033 | 7.283588 | 8.17E-07 | 1.73E-05 | -0.15888 |
| MSMO1 | 2.617393 | 2.613929 | 2.620858 | 1.38079 | 7.238338 | 8.91E-07 | 1.87E-05 | -0.25953 |
| MRPS9 | 3.044866 | 2.418714 | 3.671019 | 1.723736 | 7.194536 | 9.69E-07 | 2.03E-05 | -0.41486 |
| FLOT1 | 2.289003 | 1.796035 | 2.781971 | 1.210963 | 7.041368 | 1.30E-06 | 2.67E-05 | -0.52858 |
| SDHAF3 | 2.47459 | 0.170415 | 4.778765 | 0.923721 | 7.028835 | 1.33E-06 | 2.73E-05 | -0.60883 |
| TMED5 | 2.18228 | 1.589209 | 2.775351 | 1.40524 | 7.044483 | 1.29E-06 | 2.66E-05 | -0.72353 |
| PQBP1 | 2.56265 | 1.952007 | 3.173292 | 1.231564 | 7.001052 | 1.41E-06 | 2.87E-05 | -0.73686 |
| UMAD1 | 2.369403 | 0.682056 | 4.05675 | 1.077013 | 4.828917 | 0.000126 | 0.001965 | -0.93346 |
| ACO2 | 2.268683 | 0.140477 | 4.396889 | 1.316509 | 6.858664 | 1.86E-06 | 3.74E-05 | -1.09854 |
| QTRT1 | 3.442982 | 1.88831 | 4.997653 | 1.977843 | 6.8593 | 1.85E-06 | 3.74E-05 | -1.12608 |
| FAM104B | 2.007063 | 0.993456 | 3.02067 | 1.113283 | 6.704513 | 2.51E-06 | 4.97E-05 | -1.23871 |
| GCHFR | 3.709967 | 2.513954 | 4.90598 | 2.025775 | 6.460125 | 4.09E-06 | 7.87E-05 | -1.85542 |
| GINS2 | 2.709001 | -0.638 | 6.056003 | 1.13673 | 6.416866 | 4.47E-06 | 8.51E-05 | -1.88028 |
| MMP24OS | 3.529966 | 2.75141 | 4.308523 | 1.962005 | 6.365356 | 4.96E-06 | 9.43E-05 | -2.12635 |
| R3HCC1 | 2.362893 | 1.924178 | 2.801609 | 1.011615 | 6.218686 | 6.68E-06 | 0.000125 | -2.18902 |
| GMFB | 2.212609 | 0.952692 | 3.472526 | 0.875928 | 3.634836 | 0.001833 | 0.022912 | -2.59653 |
| CBX1 | 2.439385 | 1.680544 | 3.198227 | 1.427948 | 6.043383 | 9.57E-06 | 0.000176 | -2.68758 |
| PET117 | 2.959902 | 1.948051 | 3.971753 | 1.858143 | 6.078905 | 8.90E-06 | 0.000165 | -2.78756 |
| SFXN4 | 2.166839 | 1.661853 | 2.671826 | 1.249208 | 6.016005 | 1.01E-05 | 0.000186 | -2.82505 |
| NR3C2 | 2.205622 | -0.72971 | 5.140952 | 1.179453 | 6.028719 | 9.87E-06 | 0.000182 | -2.82904 |
| ALG3 | 2.436979 | 2.140497 | 2.733462 | 1.307249 | 5.908004 | 1.27E-05 | 0.00023 | -2.91305 |
| C8orf82 | 2.802414 | 2.802328 | 2.8025 | 1.064694 | 3.703489 | 0.001571 | 0.019956 | -3.01453 |
| ECSIT | 3.420267 | 2.866547 | 3.973987 | 1.219854 | 3.718712 | 0.001518 | 0.01932 | -3.10885 |
| ZNF22 | 3.060888 | 2.431157 | 3.690618 | 1.888848 | 5.874973 | 1.36E-05 | 0.000245 | -3.16983 |
| RHOF | 2.94349 | 2.240266 | 3.646713 | 1.751 | 5.83653 | 1.47E-05 | 0.000264 | -3.26822 |
| MRPL1 | 2.759888 | 1.463961 | 4.055816 | 1.304657 | 5.78498 | 1.64E-05 | 0.000292 | -3.33834 |
| SCYL2 | 2.188636 | 2.187996 | 2.189276 | 1.227669 | 5.771178 | 1.69E-05 | 0.0003 | -3.34408 |
| SRSF2 | 2.003466 | 2.003156 | 2.003776 | 0.967469 | 5.697387 | 1.97E-05 | 0.000348 | -3.3664 |
| VAMP3 | 2.230182 | 1.602781 | 2.857583 | 1.242426 | 5.739752 | 1.80E-05 | 0.000319 | -3.46064 |
| ZAR1 | 2.055048 | 1.657868 | 2.452227 | 1.145776 | 5.66365 | 2.12E-05 | 0.000373 | -3.54314 |
| ECHDC1 | 2.405591 | 1.798266 | 3.012916 | 1.421871 | 5.700651 | 1.96E-05 | 0.000346 | -3.56433 |
| VSIG2 | 3.599777 | 2.004296 | 5.195259 | 2.007373 | 5.639449 | 2.23E-05 | 0.000391 | -3.72519 |
| NIPSNAP3A | 3.069046 | 0.44227 | 5.695823 | 1.554338 | 5.589474 | 2.48E-05 | 0.000433 | -3.7396 |
| POLR2G | 2.79577 | 1.809943 | 3.781598 | 1.836376 | 5.573093 | 2.57E-05 | 0.000447 | -3.76254 |
| FUCA2 | 2.728046 | 2.263576 | 3.192516 | 1.631229 | 5.600982 | 2.42E-05 | 0.000423 | -3.78953 |
| ATPAF1 | 3.00056 | 2.628135 | 3.372985 | 1.741297 | 5.545591 | 2.72E-05 | 0.000472 | -3.86223 |
| NUDC | 2.355102 | 1.744796 | 2.965408 | 1.740297 | 5.511019 | 2.93E-05 | 0.000505 | -3.92898 |
| DPM2 | 2.634481 | 1.835302 | 3.433661 | 1.319774 | 5.273361 | 4.87E-05 | 0.000816 | -4.36661 |
| GOLGA5 | 2.336888 | 1.828551 | 2.845225 | 1.2583 | 5.292422 | 4.68E-05 | 0.000785 | -4.42948 |
| CCDC124 | 3.404749 | 2.985173 | 3.824324 | 2.037902 | 5.15842 | 6.25E-05 | 0.001025 | -4.762 |
| APOC1 | 3.401035 | 2.850621 | 3.951449 | 1.899563 | 5.11662 | 6.85E-05 | 0.001112 | -4.78699 |
| MT1H | 3.695767 | 2.195583 | 5.19595 | 1.737959 | 5.077434 | 7.46E-05 | 0.001204 | -4.89096 |
| MYO1B | 2.06177 | 1.470182 | 2.653359 | 1.212623 | 5.052306 | 7.88E-05 | 0.001269 | -4.92469 |
| CTSA | 2.573179 | 2.276685 | 2.869672 | 1.506444 | 5.04328 | 8.03E-05 | 0.00129 | -4.94791 |
| NOB1 | 2.176812 | 1.325793 | 3.027831 | 1.433758 | 5.013069 | 8.58E-05 | 0.001372 | -5.02925 |
| ERI3 | 2.418652 | 1.933861 | 2.903443 | 1.506631 | 4.937971 | 0.000101 | 0.001602 | -5.2146 |
| RNF167 | 2.092779 | 0.997214 | 3.188344 | 1.518743 | 4.916788 | 0.000106 | 0.001673 | -5.28941 |
| FZD5 | 2.074947 | 1.660916 | 2.488978 | 1.306024 | 4.879538 | 0.000115 | 0.001804 | -5.39514 |
| SMIM24 | 2.509003 | 1.932175 | 3.085832 | 1.195596 | 4.79683 | 0.000138 | 0.002132 | -5.41253 |
| CDCA7 | 2.007039 | 1.321438 | 2.69264 | 1.342108 | 4.815575 | 0.000132 | 0.002053 | -5.52106 |
| SLAIN2 | 2.503268 | 0.517553 | 4.488983 | 1.654989 | 4.692049 | 0.000174 | 0.002638 | -5.77225 |
| RAMP2 | 2.562878 | 2.246831 | 2.878926 | 1.407561 | 4.470399 | 0.000284 | 0.004183 | -6.15971 |
| PLA2G2A | -2.40297 | -2.67877 | -2.12716 | 5.445552 | -4.47957 | 0.000279 | 0.004103 | -6.26186 |
| FRA10AC1 | 2.597565 | 1.932592 | 3.262537 | 1.784234 | 4.489994 | 0.000272 | 0.004022 | -6.29118 |
| DCTN6 | 2.46058 | 0.933868 | 3.987293 | 1.722936 | 4.421763 | 0.000317 | 0.004621 | -6.38547 |
| ANAPC5 | 2.244461 | 1.894544 | 2.594378 | 1.499058 | 4.376223 | 0.000351 | 0.005088 | -6.47927 |
| CPEB4 | 2.170472 | 1.590245 | 2.750698 | 1.534287 | 4.40705 | 0.000328 | 0.004762 | -6.48573 |
| UBAC1 | 2.422318 | 1.903208 | 2.941427 | 1.533242 | 4.307527 | 0.000409 | 0.005913 | -6.66032 |
| CCT8 | 2.262592 | 2.262455 | 2.262729 | 1.472151 | 4.236113 | 0.00048 | 0.006834 | -6.80559 |
| FIS1 | 3.15974 | 2.676863 | 3.642618 | 3.313608 | 4.178578 | 0.000546 | 0.007684 | -6.98974 |
| S100P | -2.40653 | -4.4701 | -0.34296 | 5.361695 | -4.08701 | 0.000671 | 0.00925 | -7.17508 |
| PIM2 | -2.71056 | -3.37664 | -2.04447 | 3.27922 | -4.09061 | 0.000665 | 0.009185 | -7.20864 |
| MRPL54 | 2.197727 | 2.18798 | 2.207474 | 2.903509 | 3.985897 | 0.000841 | 0.011361 | -7.37421 |
| PKP3 | 2.242736 | 1.948856 | 2.536617 | 1.657891 | 3.966333 | 0.000879 | 0.011847 | -7.47729 |
| TXNL4A | 2.740742 | 1.254701 | 4.226782 | 1.959008 | 3.904483 | 0.00101 | 0.013476 | -7.59001 |
| PSMD7 | -2.76606 | -3.09546 | -2.43666 | 3.974223 | -3.88269 | 0.001061 | 0.014054 | -7.61306 |
| PKIG | 2.294194 | 1.043281 | 3.545106 | 1.626934 | 3.825928 | 0.001205 | 0.015793 | -7.70669 |
| DHRS11 | 2.832458 | -0.06152 | 5.726434 | 2.232465 | 3.704095 | 0.001584 | 0.02011 | -8.05798 |
| GTF3A | 3.181624 | 3.178957 | 3.18429 | 2.858464 | 3.635697 | 0.001847 | 0.02305 | -8.22108 |
| FERMT1 | 2.566723 | 1.933945 | 3.199501 | 2.145787 | 3.422914 | 0.002973 | 0.035575 | -8.70165 |
| TTC19 | 2.010088 | 1.557543 | 2.462633 | 1.394691 | 3.387022 | 0.003221 | 0.038333 | -8.74945 |
| MGAT1 | 2.046709 | 1.495983 | 2.597434 | 1.487852 | 3.269718 | 0.004182 | 0.04838 | -9.00111 |
