# Supplementary material for: Ultra-high Magnification Endocytoscopy and Molecular Markers for Defining Endoscopic and Histologic Remission in Ulcerative Colitis—An Exploratory Study to Define Deep Remission
Source: Inflamm Bowel Dis. 2021 May 21;27(11):1719–30. doi: 10.1093/ibd/izab059 (PMC8528147; doi:10.1093/ibd/izab059)
Supplement: izab059_suppl_Supplementary_Table_2 [file izab059_suppl_supplementary_table_2.docx]

Supplementary file 2: Differentially expressed genes common in healed mucosa defined by A) ECSS and RHI and B)ECSS and Nancy scores

1. Overlapping between healed mucosa defined by ECSS and RHI score

| Overlapping between healed mucosa defined by ECSS and RHI score |
| --- |
| USP2 |
| CCZ1 |
| CYP2B6 |
| RMDN2 |
| TSNAX |
| ENPP4 |
| TUSC2 |
| MED31 |
| MCU |
| RNASET2 |
| FNTA |
| GLRX5 |
| TMEM56 |
| MMP24OS |
| TUFM |
| FAM104B |
| NUDC |
| ASL |
| ACO2 |
| TRPT1 |
| ERI3 |
| CPEB4 |
| SCYL2 |
| DHRSX_1 |
| NUDCD2 |

1. Overlapping between healed mucosa defined by ECSS and NHI index

| RBM19 |
| --- |
| USP2 |
| RNF4 |
| CCZ1 |
| CYP2B6 |
| TRNP |
| AIMP2 |
| PEPD |
| ATPAF1 |
| RMDN2 |
| TSNAX |
| UBFD1 |
| DHX8 |
| SLC22A18AS |
| ANGEL2 |
| ENPP4 |
| WEE1 |
| TUSC2 |
| MED31 |
| C1orf35 |
| BTN3A1 |
| CPTP |
| ANKLE2 |
| NTAN1 |
| MCU |
| RAB32 |
| POLR3GL |
| RNASET2 |
| BIN1 |
| C6orf203 |
| C1orf123 |
| SLC35B1 |
| TNIP2 |
| AP1AR |
| AKAP1 |
| RCN2 |
| ACAT1 |
| FNTA |
| TMEM171 |
| GLRX5 |
| TMEM56 |
| CSE1L |
| CRYL1 |
| MMP24OS |
| PPID |
| PQBP1 |
| UMAD1 |
| TUFM |
| FAM104B |
| PPP1R35 |
| FLOT1 |
| SDHAF3 |
| NR3C2 |
| GINS2 |
| HOXB9 |
| GTF3A |
| ALG3 |
| VSIG2 |
| NUDC |
| POGLUT1 |
| ASL |
| ACO2 |
| FZD5 |
| CCDC124 |
| VSIG10 |
| R3HCC1 |
| OXSR1 |
| CCT8 |
| DPM2 |
| TRPT1 |
| TXNL4A |
| PCK1 |
| ERI3 |
| CPEB4 |
| SCYL2 |
| PKP3 |
| DHRSX_1 |
| APOC1 |
| SMIM24 |
