# Supplementary material for: Ultra-high Magnification Endocytoscopy and Molecular Markers for Defining Endoscopic and Histologic Remission in Ulcerative Colitis—An Exploratory Study to Define Deep Remission
Source: Inflamm Bowel Dis. 2021 May 21;27(11):1719–30. doi: 10.1093/ibd/izab059 (PMC8528147; doi:10.1093/ibd/izab059)
Supplement: izab059_suppl_Supplementary_Table_3 [file izab059_suppl_supplementary_table_3.docx]

**Supplementary Table 3:** Partial Least Square Discriminant Analysis ( PLS/DA) of healed vs non-healed mucosa defined by A)ECSS, B)Mayo, C)RHI and D) Nancy scores. Differentially expressed genes for which VIP>1 are listed.

**A) ECSS**

| Sample | VIP score | logFC | CI.L | CI.R | AveExpr | t | P.Value | adj.P.Val | B |
| --- | --- | --- | --- | --- | --- | --- | --- | --- | --- |
| VSIG2 | 1.5303 | 3.6804 | 2.2300 | 5.1309 | 2.0074 | 5.9887 | 0.0000 | 0.0004 | -2.2822 |
| CPEB4 | 1.4385 | 2.3354 | -0.4331 | 5.1039 | 1.5343 | 4.6438 | 0.0002 | 0.0052 | -5.2705 |
| HECA | 1.3919 | 2.1877 | 1.4794 | 2.8959 | 0.6561 | 48.5896 | 0.0000 | 0.0000 | 34.8291 |
| MMP24OS | 1.3518 | 4.0183 | 3.7033 | 4.3332 | 1.9620 | 7.4089 | 0.0000 | 0.0000 | 0.6773 |
| LCN2 | 1.3464 | -4.6598 | -6.4826 | -2.8369 | 4.9245 | -9.2771 | 0.0000 | 0.0000 | 4.2099 |
| EDEM1 | 1.3400 | 2.3269 | 1.0982 | 3.5557 | 1.1472 | 7.3221 | 0.0000 | 0.0000 | 0.5098 |
| GTF3A | 1.3378 | 3.7488 | 3.0302 | 4.4675 | 2.8585 | 6.2990 | 0.0000 | 0.0002 | -1.6372 |
| PRDM1 | 1.3348 | -2.0688 | -3.6852 | -0.4524 | 2.4410 | -4.5819 | 0.0002 | 0.0059 | -5.4043 |
| MPP7 | 1.3183 | 2.6745 | 1.0788 | 4.2701 | 1.1744 | 17.2422 | 0.0000 | 0.0000 | 14.9757 |
| B4GALT5 | 1.3159 | 2.2686 | 1.7890 | 2.7482 | 1.2972 | 4.4443 | 0.0003 | 0.0078 | -5.6793 |
| FZD5 | 1.3096 | 2.1769 | 1.6420 | 2.7118 | 1.3060 | 5.5447 | 0.0000 | 0.0009 | -3.2343 |
| TTC38 | 1.2866 | 2.3298 | 1.6715 | 2.9881 | 1.5664 | 4.8179 | 0.0001 | 0.0036 | -4.8687 |
| AKAP1 | 1.2815 | 2.9936 | 2.5635 | 3.4237 | 1.4370 | 9.4346 | 0.0000 | 0.0000 | 4.3993 |
| PDZK1IP1 | 1.2762 | -4.4977 | -4.4978 | -4.4976 | 4.0069 | -6.3297 | 0.0000 | 0.0002 | -1.5413 |
| ATPAF1 | 1.2603 | 4.0636 | 3.5375 | 4.5897 | 1.7413 | 27.6717 | 0.0000 | 0.0000 | 23.7522 |
| YIPF6 | 1.2512 | 2.3806 | 1.8869 | 2.8743 | 1.4709 | 4.4741 | 0.0003 | 0.0073 | -5.6193 |
| UMAD1 | 1.2477 | 2.7888 | 2.7887 | 2.7888 | 1.0770 | 5.1064 | 0.0001 | 0.0020 | -0.2002 |
| BIN3 | 1.2415 | 3.5293 | 3.2104 | 3.8482 | 0.8036 | 16.3337 | 0.0000 | 0.0000 | 14.0742 |
| ZMAT2 | 1.2383 | -2.5804 | -4.0593 | -1.1015 | 2.4127 | -4.2936 | 0.0004 | 0.0106 | -6.0522 |
| CCDC124 | 1.2366 | 3.5845 | 3.0655 | 4.1035 | 2.0379 | 5.4684 | 0.0000 | 0.0010 | -3.3870 |
| RCC1L | 1.2326 | 2.0858 | 1.7408 | 2.4309 | 0.5693 | 23.4363 | 0.0000 | 0.0000 | 22.0811 |
| ENPP4 | 1.2114 | 2.1914 | 2.1913 | 2.1914 | 1.0357 | 16.6223 | 0.0000 | 0.0000 | 14.2534 |
| FBLN1 | 1.2081 | -2.1599 | -3.0981 | -1.2218 | 1.9914 | -3.9407 | 0.0009 | 0.0217 | -6.8479 |
| TGFBR2 | 1.1950 | -2.3281 | -3.0600 | -1.5962 | 1.7201 | -5.5964 | 0.0000 | 0.0008 | -3.1228 |
| TXNL4A | 1.1902 | 3.2801 | 0.2329 | 6.3273 | 1.9590 | 4.9004 | 0.0001 | 0.0031 | -4.6558 |
| RNF4 | 1.1816 | 2.7051 | 2.1923 | 3.2179 | 0.9076 | 36.2566 | 0.0000 | 0.0000 | 31.5177 |
| PCLAF | 1.1739 | -3.1665 | -4.0728 | -2.2601 | 2.5259 | -6.7103 | 0.0000 | 0.0001 | -0.7473 |
| UBFD1 | 1.1706 | 3.0781 | 1.1064 | 5.0498 | 0.9747 | 17.5903 | 0.0000 | 0.0000 | 18.4169 |
| COMMD4 | 1.1617 | 2.9569 | 1.0157 | 4.8980 | 1.6941 | 5.1416 | 0.0001 | 0.0019 | -4.1192 |
| ANGEL2 | 1.1537 | 2.3189 | 1.7461 | 2.8916 | 0.8542 | 14.4059 | 0.0000 | 0.0000 | 15.2839 |
| DHRSX_1 | 1.1512 | 2.6338 | 1.9654 | 3.3022 | 1.6589 | 3.9415 | 0.0009 | 0.0217 | -6.8282 |
| PKP3 | 1.1457 | 2.2816 | 1.5131 | 3.0501 | 1.6579 | 4.1273 | 0.0006 | 0.0150 | -6.4316 |
| KIAA1522 | 1.1415 | 2.0851 | 1.7257 | 2.4444 | 0.9627 | 9.2842 | 0.0000 | 0.0000 | 4.4094 |
| USP2 | 1.1334 | 2.9332 | 2.3656 | 3.5008 | 1.3222 | 59.3829 | 0.0000 | 0.0000 | 38.7075 |
| VSIG10 | 1.1198 | 2.0819 | 1.4030 | 2.7607 | 1.2704 | 5.4185 | 0.0000 | 0.0011 | -3.4784 |
| RCN2 | 1.1063 | 3.0068 | 2.4044 | 3.6093 | 1.4137 | 8.6244 | 0.0000 | 0.0000 | 3.0802 |
| CCT8 | 1.0923 | 2.6618 | 0.4835 | 4.8402 | 1.4722 | 4.9723 | 0.0001 | 0.0027 | -4.4119 |
| GCNT3 | 1.0883 | -2.2634 | -3.2409 | -1.2860 | 3.6774 | -5.6166 | 0.0000 | 0.0008 | -3.0040 |
| CRYZL1 | 1.0866 | 2.9367 | 2.3569 | 3.5166 | 1.4439 | 7.3309 | 0.0000 | 0.0000 | 0.6562 |
| TMEM208 | 1.0829 | 3.4337 | 1.3590 | 5.5083 | 2.6472 | 4.1592 | 0.0006 | 0.0141 | -6.3233 |
| DENND2A | 1.0782 | 2.0020 | 1.5332 | 2.4709 | 0.7002 | 11.3830 | 0.0000 | 0.0000 | 7.9070 |
| TRPT1 | 1.0775 | 2.8157 | 2.8131 | 2.8184 | 1.7188 | 4.9594 | 0.0001 | 0.0027 | -4.4743 |
| DHX8 | 1.0768 | 2.1362 | 0.7759 | 3.4965 | 0.8978 | 20.4134 | 0.0000 | 0.0000 | 18.2396 |
| LSM14A | 1.0654 | -2.2022 | -2.6797 | -1.7248 | 1.9070 | -5.1240 | 0.0001 | 0.0020 | -4.1368 |
| RNASET2 | 1.0614 | 3.8134 | 2.6872 | 4.9397 | 2.6784 | 10.7636 | 0.0000 | 0.0000 | 6.5488 |
| TUFM | 1.0537 | 2.8427 | 2.3009 | 3.3845 | 2.1252 | 6.9258 | 0.0000 | 0.0001 | -0.2903 |
| DAAM1 | 1.0516 | -2.1926 | -3.7496 | -0.6355 | 1.6000 | -4.6421 | 0.0002 | 0.0052 | -5.2407 |
| AKIRIN2 | 1.0493 | 3.1327 | 2.5593 | 3.7060 | 1.2356 | 20.1948 | 0.0000 | 0.0000 | 17.9049 |
| RNASE6 | 1.0493 | 2.0201 | 1.0597 | 2.9804 | 1.0363 | 7.3437 | 0.0000 | 0.0000 | 0.5923 |
| RAB32 | 1.0442 | 3.6859 | 3.1150 | 4.2567 | 1.2659 | 11.4369 | 0.0000 | 0.0000 | 7.7601 |
| GLRX5 | 1.0421 | 3.9266 | 3.3494 | 4.5037 | 1.9266 | 7.8672 | 0.0000 | 0.0000 | 1.6532 |
| ANKLE2 | 1.0415 | 2.2458 | -1.0396 | 5.5312 | 1.0406 | 12.8360 | 0.0000 | 0.0000 | 9.6917 |
| PPP2R3C | 1.0353 | 3.3795 | 0.8758 | 5.8831 | 1.5785 | 8.4275 | 0.0000 | 0.0000 | 2.6682 |
| CSE1L | 1.0344 | 2.3534 | 2.3522 | 2.3547 | 1.1863 | 7.6934 | 0.0000 | 0.0000 | 1.3021 |
| LACTB2 | 1.0307 | 2.5480 | 0.9784 | 4.1176 | 1.0739 | 37.6244 | 0.0000 | 0.0000 | 31.5630 |
| STAT6 | 1.0237 | 2.1050 | 1.6687 | 2.5412 | 0.7731 | 9.5978 | 0.0000 | 0.0000 | 9.2280 |
| SCYL2 | 1.0236 | 2.0535 | -0.9668 | 5.0739 | 1.2277 | 4.5664 | 0.0002 | 0.0060 | -5.3416 |
| CCZ1 | 1.0209 | 2.5538 | 1.9124 | 3.1951 | 1.0210 | 32.3927 | 0.0000 | 0.0000 | 27.1416 |
| SDHAF3 | 1.0034 | 2.4039 | 2.0608 | 2.7471 | 0.9237 | 6.7230 | 0.0000 | 0.0001 | -0.5137 |
| FSIP2-AS1 | 1.0021 | 2.5380 | 2.2314 | 2.8447 | 0.8129 | 15.2968 | 0.0000 | 0.0000 | 16.6155 |

**B) Mayo**

| Sample | VIP score | logFC | CI.L | CI.R | AveExpr | t | P.Value | adj.P.Val | B |
| --- | --- | --- | --- | --- | --- | --- | --- | --- | --- |
| IFI35 | 1.3778 | 3.8121 | 2.7093 | 4.9150 | 1.3518 | 160.0525 | 0.0000 | 0.0000 | 63.6816 |
| IL6R | 1.0552 | 2.3861 | 1.2425 | 3.5297 | 0.8896 | 154.5484 | 0.0000 | 0.0000 | 63.0031 |
| CDS1 | 1.2857 | 3.2132 | 2.7621 | 3.6643 | 1.3032 | 121.9818 | 0.0000 | 0.0000 | 58.3309 |
| C6orf203 | 1.1594 | 3.7006 | 2.7191 | 4.6820 | 1.4850 | 98.1487 | 0.0000 | 0.0000 | 53.7534 |
| C5orf51 | 1.0052 | 3.0978 | 2.7715 | 3.4241 | 1.4698 | 82.8061 | 0.0000 | 0.0000 | 50.3908 |
| GINS2 | 1.0722 | 3.5830 | 1.2842 | 5.8818 | 1.2506 | 78.0673 | 0.0000 | 0.0000 | 49.1920 |
| SMIM24 | 1.3663 | 3.5972 | 3.2542 | 3.9403 | 1.3098 | 75.8962 | 0.0000 | 0.0000 | 49.0929 |
| PEPD | 1.1416 | 3.7442 | 3.4783 | 4.0102 | 1.4869 | 75.8492 | 0.0000 | 0.0000 | 48.4934 |
| METTL7B | 1.0931 | 3.5506 | 1.7443 | 5.3569 | 1.2857 | 68.6251 | 0.0000 | 0.0000 | 46.4825 |
| STX10 | 1.0637 | 3.7075 | 2.4293 | 4.9858 | 1.3343 | 52.6817 | 0.0000 | 0.0000 | 41.9775 |
| EMC2 | 1.0558 | 2.6804 | 0.6189 | 4.7419 | 1.0009 | 52.4449 | 0.0000 | 0.0000 | 40.9856 |
| EHHADH | 1.2524 | 2.2250 | 2.1541 | 2.2959 | 0.7488 | 43.3390 | 0.0000 | 0.0000 | 37.0141 |
| TPSB2 | 1.1268 | 3.8520 | 3.3331 | 4.3708 | 2.0148 | 43.2027 | 0.0000 | 0.0000 | 36.8431 |
| DCAF11 | 1.1634 | 2.5382 | 2.0476 | 3.0289 | 1.0000 | 42.1797 | 0.0000 | 0.0000 | 36.4318 |
| UBP1 | 1.1794 | 2.6467 | 2.6467 | 2.6467 | 1.2621 | 38.4861 | 0.0000 | 0.0000 | 34.6773 |
| AP1AR | 1.1187 | 2.7542 | 2.1355 | 3.3730 | 1.0837 | 37.3951 | 0.0000 | 0.0000 | 33.9791 |
| ELAVL1 | 1.1616 | 2.1652 | 1.7065 | 2.6238 | 0.7610 | 37.0095 | 0.0000 | 0.0000 | 33.8460 |
| CRYL1 | 1.3015 | 3.6048 | 3.1744 | 4.0351 | 1.5798 | 35.4014 | 0.0000 | 0.0000 | 33.0631 |
| COMT | 1.0662 | 2.7857 | 2.2185 | 3.3529 | 1.0758 | 34.7407 | 0.0000 | 0.0000 | 32.5202 |
| QTRT1 | 1.5812 | 4.2772 | 2.6565 | 5.8979 | 2.1480 | 33.9525 | 0.0000 | 0.0000 | 31.8657 |
| UBAC1 | 1.4008 | 3.3444 | 2.8996 | 3.7893 | 1.5449 | 30.7157 | 0.0000 | 0.0000 | 30.0828 |
| MGAT1 | 1.0870 | 2.7939 | 2.2150 | 3.3728 | 1.5517 | 30.6216 | 0.0000 | 0.0000 | 29.9634 |
| RCOR1 | 1.1313 | 2.1971 | 1.3878 | 3.0064 | 0.7741 | 30.5702 | 0.0000 | 0.0000 | 29.9496 |
| PICALM | 1.0578 | 2.5087 | 1.9432 | 3.0743 | 0.9288 | 25.1342 | 0.0000 | 0.0000 | 27.7319 |
| CPTP | 1.4722 | 3.0465 | 2.3434 | 3.7495 | 1.0697 | 26.7015 | 0.0000 | 0.0000 | 27.1053 |
| NIPSNAP3A | 1.0894 | 3.5866 | 1.7669 | 5.4063 | 1.4803 | 24.6343 | 0.0000 | 0.0000 | 25.5876 |
| C1orf123 | 1.2944 | 4.0419 | 3.6749 | 4.4088 | 1.5035 | 23.6021 | 0.0000 | 0.0000 | 24.5764 |
| MT1H | 1.3961 | 4.8284 | 4.3054 | 5.3515 | 1.8994 | 21.9361 | 0.0000 | 0.0000 | 23.2069 |
| GON7 | 1.1908 | 4.3643 | 2.6078 | 6.1208 | 2.2111 | 21.8737 | 0.0000 | 0.0000 | 23.1100 |
| KLHL24 | 1.4107 | 2.2802 | 1.8600 | 2.7004 | 1.1593 | 21.6825 | 0.0000 | 0.0000 | 22.9386 |
| PDE8A | 1.5866 | 2.5266 | 1.5291 | 3.5242 | 1.0112 | 19.9291 | 0.0000 | 0.0000 | 21.2522 |
| METAP1 | 1.1693 | 2.1656 | 0.7562 | 3.5750 | 0.7965 | 18.9432 | 0.0000 | 0.0000 | 20.4953 |
| DCTN3 | 1.0851 | 2.7671 | 2.1884 | 3.3458 | 1.4374 | 17.6706 | 0.0000 | 0.0000 | 19.0038 |
| ABCG2 | 1.2701 | 2.2724 | 1.7485 | 2.7963 | 1.2108 | 17.0196 | 0.0000 | 0.0000 | 18.2235 |
| POLR2B | 1.2430 | 2.5212 | 2.2889 | 2.7534 | 1.2719 | 16.8248 | 0.0000 | 0.0000 | 18.0531 |
| GTF2B | 1.1257 | 3.1600 | 2.5352 | 3.7849 | 1.1406 | 16.0240 | 0.0000 | 0.0000 | 17.1122 |
| TMEM87B | 1.2126 | 3.1159 | 0.6544 | 5.5773 | 1.5149 | 15.7317 | 0.0000 | 0.0000 | 16.7399 |
| APOC1 | 1.0119 | 4.2697 | 1.4332 | 7.1063 | 1.8091 | 15.4120 | 0.0000 | 0.0000 | 16.3973 |
| JUP | 1.0521 | 2.6599 | 1.3325 | 3.9873 | 1.2877 | 14.5281 | 0.0000 | 0.0000 | 15.1796 |
| RNASET2 | 1.1120 | 3.7692 | 3.1728 | 4.3655 | 2.7785 | 14.3161 | 0.0000 | 0.0000 | 14.8588 |
| HBB | 1.3560 | 4.5048 | 3.5528 | 5.4568 | 1.8016 | 14.1838 | 0.0000 | 0.0000 | 14.7017 |
| RBM19 | 1.0868 | 2.1274 | 2.1272 | 2.1276 | 0.8411 | 13.6089 | 0.0000 | 0.0000 | 14.1358 |
| ASL | 1.4580 | 3.7789 | 1.9089 | 5.6489 | 1.7588 | 13.5111 | 0.0000 | 0.0000 | 13.7729 |
| CDKN2B-AS1 | 1.0002 | 2.2798 | 1.8977 | 2.6619 | 1.2867 | 12.4561 | 0.0000 | 0.0000 | 12.3824 |
| PDZK1IP1 | 1.3849 | -4.9404 | -7.7361 | -2.1447 | 3.8767 | -12.4044 | 0.0000 | 0.0000 | 12.1684 |
| DHX9 | 1.0501 | 2.2710 | 1.8577 | 2.6842 | 1.1880 | 12.1958 | 0.0000 | 0.0000 | 12.0140 |
| PCK1 | 1.2858 | 2.9727 | 1.9149 | 4.0306 | 1.6629 | 12.1734 | 0.0000 | 0.0000 | 11.8643 |
| VPS4A | 1.2702 | 3.3838 | 1.0166 | 5.7511 | 1.8534 | 12.1893 | 0.0000 | 0.0000 | 11.8618 |
| SAFB2 | 1.1189 | 2.1429 | 1.2518 | 3.0341 | 0.8743 | 12.0153 | 0.0000 | 0.0000 | 11.7014 |
| ENDOD1 | 1.2616 | 2.2098 | 1.4900 | 2.9297 | 1.0994 | 11.5488 | 0.0000 | 0.0000 | 10.9964 |
| DDT | 1.2041 | 3.6538 | 2.0230 | 5.2846 | 3.6498 | 11.0182 | 0.0000 | 0.0000 | 10.1121 |
| KRTCAP3 | 1.2595 | 4.3823 | 2.8639 | 5.9007 | 1.8169 | 10.9691 | 0.0000 | 0.0000 | 9.9977 |
| MEP1A | 1.0052 | 2.9720 | 1.7486 | 4.1954 | 1.5615 | 10.3383 | 0.0000 | 0.0000 | 9.1009 |
| NDUFS2 | 1.1225 | 2.5687 | 1.8871 | 3.2503 | 1.5616 | 10.3562 | 0.0000 | 0.0000 | 8.9773 |
| BIN1 | 1.0367 | 2.0638 | 0.3435 | 3.7842 | 0.8919 | 9.8663 | 0.0000 | 0.0000 | 8.2842 |
| DPP7 | 1.2650 | 2.5026 | 2.1157 | 2.8896 | 1.3627 | 9.8948 | 0.0000 | 0.0000 | 8.2460 |
| SMIM26 | 1.2521 | 4.2236 | 3.4661 | 4.9811 | 3.9252 | 9.9032 | 0.0000 | 0.0000 | 8.2253 |
| DENR | 1.0747 | 2.7557 | 2.4626 | 3.0487 | 1.4071 | 9.7461 | 0.0000 | 0.0000 | 8.0016 |
| TMEM209 | 1.0916 | 2.1129 | 1.3942 | 2.8315 | 0.9281 | 9.5170 | 0.0000 | 0.0000 | 7.6467 |
| DNAJC2 | 1.3551 | 2.8495 | 2.1280 | 3.5710 | 1.5294 | 9.3583 | 0.0000 | 0.0000 | 7.2368 |
| DUOX2 | 1.1469 | -3.2100 | -4.2797 | -2.1402 | 2.0205 | -9.3447 | 0.0000 | 0.0000 | 7.2127 |
| FAM120AOS | 1.1979 | 2.8048 | 0.9542 | 4.6554 | 1.5312 | 8.8976 | 0.0000 | 0.0000 | 6.4036 |
| NAA60 | 1.3420 | 2.2744 | 1.8916 | 2.6572 | 0.9091 | 8.6304 | 0.0000 | 0.0000 | 5.9885 |
| WASL | 1.0811 | 3.0124 | 1.7752 | 4.2496 | 1.9088 | 8.6789 | 0.0000 | 0.0000 | 5.9800 |
| DHRS11 | 1.2850 | 3.4634 | 1.2053 | 5.7215 | 2.3606 | 8.5751 | 0.0000 | 0.0000 | 5.7885 |
| NAAA | 1.1887 | 2.6248 | 1.8186 | 3.4311 | 1.7538 | 8.2361 | 0.0000 | 0.0000 | 5.1256 |
| IDH3G | 1.0923 | 3.0976 | 2.6169 | 3.5783 | 1.7145 | 8.0539 | 0.0000 | 0.0000 | 4.8727 |
| TMEM170A | 1.2127 | 2.5070 | 1.4686 | 3.5453 | 1.7231 | 8.1034 | 0.0000 | 0.0000 | 4.8678 |
| ETFDH | 1.0619 | 2.4540 | 1.3968 | 3.4818 | 1.3846 | 8.0281 | 0.0000 | 0.0000 | 4.7585 |
| SDHAF3 | 1.2346 | 2.6456 | 2.2872 | 3.0041 | 1.1319 | 7.9110 | 0.0000 | 0.0000 | 4.6060 |
| SURF1 | 1.0409 | 3.1509 | 2.6493 | 3.6525 | 2.8415 | 7.3862 | 0.0000 | 0.0000 | 3.4214 |
| AAMDC | 1.1706 | 3.0919 | 2.8419 | 3.3419 | 1.5161 | 7.3153 | 0.0000 | 0.0000 | 3.3438 |
| VPS28 | 1.0197 | 3.3980 | 3.1347 | 3.6613 | 2.9162 | 7.3140 | 0.0000 | 0.0000 | 3.3013 |
| TOR1AIP1 | 1.1553 | 2.3812 | 0.9649 | 3.7975 | 1.4060 | 7.2974 | 0.0000 | 0.0000 | 3.2673 |
| OSBPL1A | 1.2142 | 2.2439 | 0.0747 | 4.4132 | 1.1594 | 7.2723 | 0.0000 | 0.0000 | 3.1991 |
| HSD17B2 | 1.0391 | 3.5545 | 2.3474 | 4.7617 | 2.0504 | 7.2038 | 0.0000 | 0.0000 | 3.0880 |
| SLC38A1 | 1.0512 | 2.0269 | 1.5812 | 2.4727 | 1.6565 | 7.1897 | 0.0000 | 0.0000 | 3.0144 |
| CUTA | 1.1430 | 3.2145 | 2.7636 | 3.6655 | 3.3172 | 7.0416 | 0.0000 | 0.0000 | 2.7274 |
| PCGF5 | 1.1740 | 2.3039 | 0.1561 | 4.4517 | 1.4253 | 6.8617 | 0.0000 | 0.0000 | 2.3388 |
| SNX2 | 1.0539 | 2.0389 | 1.5566 | 2.5213 | 1.4644 | 6.6796 | 0.0000 | 0.0001 | 1.9545 |
| CREG1 | 1.0627 | 2.3530 | -0.3660 | 5.0720 | 1.3357 | 6.6326 | 0.0000 | 0.0001 | 1.8776 |
| RGS10 | 1.1943 | 3.0957 | 3.0744 | 3.1170 | 1.5377 | 6.5683 | 0.0000 | 0.0001 | 1.7545 |
| CNOT1 | 1.2493 | 2.0469 | 1.2221 | 2.8717 | 1.4190 | 6.5284 | 0.0000 | 0.0001 | 1.6030 |
| AIP | 1.0640 | 2.7929 | 1.8062 | 3.7796 | 1.1427 | 6.4782 | 0.0000 | 0.0001 | 1.5996 |
| LCN2 | 1.1999 | -4.2918 | -4.8056 | -3.7780 | 4.7525 | -6.4988 | 0.0000 | 0.0001 | 1.5885 |
| TLK1 | 1.1708 | 2.2638 | 1.3867 | 3.1410 | 1.6290 | 6.4256 | 0.0000 | 0.0001 | 1.4067 |
| SMIM31 | 1.0672 | 2.9542 | 1.1073 | 4.8012 | 2.0235 | 6.4142 | 0.0000 | 0.0001 | 1.3950 |
| MT1G | 1.3051 | 4.5469 | 4.2089 | 4.8850 | 4.6498 | 6.4193 | 0.0000 | 0.0001 | 1.3782 |
| MT1F | 1.3225 | 4.2595 | 3.7539 | 4.7651 | 2.2089 | 6.4065 | 0.0000 | 0.0001 | 1.3698 |
| C1orf115 | 1.3646 | 2.3083 | 1.7434 | 2.8732 | 1.4543 | 6.2509 | 0.0000 | 0.0001 | 1.0220 |
| PABPN1 | 1.3307 | 2.4463 | 1.9672 | 2.9255 | 1.8991 | 6.2391 | 0.0000 | 0.0001 | 0.9665 |
| FRA10AC1 | 1.0910 | 2.4987 | 1.7821 | 3.2152 | 1.8903 | 5.9706 | 0.0000 | 0.0002 | 0.3792 |
| RAB11B | 1.3986 | 3.1120 | 1.3116 | 4.9125 | 2.1656 | 5.9233 | 0.0000 | 0.0002 | 0.2675 |
| PLIN3 | 1.3445 | 2.9826 | 2.2671 | 3.6981 | 2.4740 | 5.9201 | 0.0000 | 0.0002 | 0.2596 |
| SFT2D1 | 1.1878 | 3.6605 | 3.5308 | 3.7903 | 3.2930 | 5.9009 | 0.0000 | 0.0002 | 0.2409 |
| GRPEL1 | 1.1027 | 2.9930 | 2.6054 | 3.3806 | 2.3347 | 5.7248 | 0.0000 | 0.0003 | -0.1583 |
| NFKBIZ | 1.2384 | -2.7780 | -3.4394 | -2.1167 | 1.5072 | -5.5964 | 0.0000 | 0.0004 | -0.4498 |
| RAB10 | 1.1116 | 2.0675 | -0.1711 | 4.3061 | 3.1027 | 5.5671 | 0.0000 | 0.0005 | -0.4948 |
| HSD11B2 | 1.0725 | 2.4535 | 0.9718 | 3.9352 | 1.5715 | 5.4830 | 0.0000 | 0.0005 | -0.6921 |
| PPP1R2 | 1.0489 | 2.4129 | 0.4242 | 4.4016 | 1.6878 | 5.3745 | 0.0000 | 0.0007 | -0.9414 |
| DUOXA2 | 1.2098 | -3.0830 | -4.1102 | -2.0557 | 2.2453 | -5.2429 | 0.0000 | 0.0009 | -1.2422 |
| THYN1 | 1.0156 | 3.0352 | 3.0297 | 3.0407 | 2.2036 | 5.1892 | 0.0001 | 0.0010 | -1.3650 |
| GSN | 1.1628 | -2.4056 | -3.2804 | -1.5308 | 2.4534 | -5.1948 | 0.0001 | 0.0010 | -1.3715 |
| FUCA2 | 1.0090 | 2.5719 | 1.9303 | 3.2135 | 1.7523 | 5.1808 | 0.0001 | 0.0010 | -1.3802 |
| ALDH3A2 | 1.0687 | 2.4176 | 1.7136 | 3.1216 | 1.9924 | 5.0776 | 0.0001 | 0.0012 | -1.6213 |
| CFTR | 1.2096 | 2.2837 | 1.8260 | 2.7414 | 2.0353 | 5.0299 | 0.0001 | 0.0013 | -1.7587 |
| ARCN1 | 1.0583 | 2.4454 | 1.7879 | 3.1028 | 1.7451 | 4.9369 | 0.0001 | 0.0016 | -1.9551 |
| MISP | 1.2641 | 2.8242 | 2.4721 | 3.1763 | 2.3163 | 4.9398 | 0.0001 | 0.0016 | -1.9688 |
| JUN | 1.1337 | -2.3037 | -2.6510 | -1.9564 | 2.7454 | -4.8655 | 0.0001 | 0.0018 | -2.1264 |
| PDCD6 | 1.1284 | 2.5592 | 1.5396 | 3.5788 | 2.8315 | 4.8509 | 0.0001 | 0.0019 | -2.1637 |
| SERINC3 | 1.3812 | 2.7648 | 2.3241 | 3.2056 | 2.0722 | 4.8413 | 0.0001 | 0.0019 | -2.1984 |
| MTIF3 | 1.2038 | 2.5977 | 1.9645 | 3.2310 | 2.0853 | 4.5842 | 0.0002 | 0.0033 | -2.7857 |
| CAT | 1.2661 | 3.0692 | 2.6045 | 3.5339 | 2.6787 | 4.5266 | 0.0002 | 0.0037 | -2.9249 |
| MKRN1 | 1.1319 | 2.4524 | 1.5158 | 3.3890 | 1.9920 | 4.4154 | 0.0003 | 0.0047 | -3.1772 |
| RIOK3 | 1.2594 | 2.5707 | 1.9929 | 3.1485 | 2.5190 | 4.0103 | 0.0007 | 0.0107 | -4.1333 |
| GINM1 | 1.2567 | 2.7076 | 1.2419 | 4.1733 | 2.2207 | 3.9711 | 0.0008 | 0.0116 | -4.2207 |
| CS | 1.0507 | 2.2078 | 2.0467 | 2.3689 | 1.7359 | 3.8992 | 0.0009 | 0.0135 | -4.3839 |
| PRKACB | 1.0531 | 2.1111 | 1.6229 | 2.5993 | 2.1290 | 3.7879 | 0.0012 | 0.0171 | -4.6352 |
| UBL3 | 1.1677 | 2.2203 | 0.2425 | 4.1981 | 1.7130 | 3.7465 | 0.0013 | 0.0184 | -4.7379 |
| HADHB | 1.3168 | 2.6766 | 2.1982 | 3.1549 | 2.4432 | 3.7438 | 0.0013 | 0.0185 | -4.7513 |
| MPST | 1.2551 | 2.7293 | 2.3006 | 3.1581 | 2.8325 | 3.7011 | 0.0015 | 0.0202 | -4.8448 |
| PIM2 | 1.1566 | -2.3799 | -3.0119 | -1.7479 | 3.1572 | -3.6767 | 0.0015 | 0.0213 | -4.8998 |
| MT2A | 1.1490 | 3.0011 | 0.8139 | 5.1884 | 4.3139 | 3.6596 | 0.0016 | 0.0220 | -4.9271 |
| NOP53 | 1.0608 | 2.4990 | 0.6576 | 4.3404 | 3.3111 | 3.6213 | 0.0018 | 0.0238 | -5.0129 |
| GUCA2A | 1.1132 | 3.0834 | 2.2929 | 3.8739 | 3.8847 | 3.4515 | 0.0026 | 0.0336 | -5.4113 |
| ARL8B | 1.1934 | 2.4567 | 1.7385 | 3.1750 | 2.1461 | 3.4236 | 0.0028 | 0.0355 | -5.4753 |
| MT1X | 1.1662 | 2.0991 | 0.0392 | 4.1591 | 5.6005 | 3.3712 | 0.0031 | 0.0396 | -5.5817 |
| CXCL1 | 1.0137 | -2.7494 | -3.2856 | -2.2133 | 2.8412 | -3.2589 | 0.0040 | 0.0496 | -5.8358 |

**C) RHI**

| Sample | VIP scores | logFC | CI.L | CI.R | AveExpr | t | P.Value | adj.P.Val | B |
| --- | --- | --- | --- | --- | --- | --- | --- | --- | --- |
| TRPT1 | 1.5907 | 3.9412 | 3.6011 | 4.2813 | 1.7188 | 11.0813 | 0.0000 | 0.0000 | 8.8145 |
| USP2 | 1.5163 | 3.0640 | 2.2829 | 3.8450 | 1.3222 | 68.2823 | 0.0000 | 0.0000 | 43.7965 |
| CCZ1 | 1.4227 | 2.6629 | 2.2479 | 3.0779 | 1.0210 | 89.4685 | 0.0000 | 0.0000 | 48.9330 |
| STAT1 | 1.3926 | -2.8620 | -4.8032 | -0.9208 | 2.1033 | -7.5876 | 0.0000 | 0.0000 | 2.7139 |
| AXL | 1.3758 | 2.8094 | 1.9010 | 3.7179 | 1.0431 | 94.1468 | 0.0000 | 0.0000 | 50.0536 |
| DYNC1LI2 | 1.3519 | 2.7302 | 0.6836 | 4.7768 | 2.1059 | 4.4036 | 0.0003 | 0.0171 | -4.1302 |
| LUZP1 | 1.3187 | 2.3053 | 1.4627 | 3.1479 | 1.2686 | 8.2587 | 0.0000 | 0.0000 | 4.0372 |
| FNTA | 1.2371 | 3.7707 | 3.1198 | 4.4215 | 1.5439 | 37.6805 | 0.0000 | 0.0000 | 31.6508 |
| PDZK1IP1 | 1.1948 | -4.2771 | -4.7010 | -3.8533 | 4.0069 | -6.1693 | 0.0000 | 0.0006 | -0.1477 |
| SBDS | 1.1903 | 2.1934 | 1.3633 | 3.0235 | 3.6336 | 4.3920 | 0.0003 | 0.0174 | -4.1210 |
| ENPP4 | 1.1712 | 2.2027 | 1.1905 | 3.2150 | 1.0357 | 12.1122 | 0.0000 | 0.0000 | 10.4117 |
| MRPL1 | 1.1541 | 2.4960 | 1.9205 | 3.0715 | 1.3047 | 7.0993 | 0.0000 | 0.0001 | 1.8465 |
| COLGALT1 | 1.1511 | 2.4484 | 1.2877 | 3.6092 | 0.8548 | 133.8343 | 0.0000 | 0.0000 | 56.8397 |
| SNORD47 | 1.1177 | -3.7743 | -5.0464 | -2.5023 | 2.6893 | -7.1462 | 0.0000 | 0.0001 | 1.8517 |
| HEPACAM2 | 1.1101 | 2.7581 | 2.0725 | 3.4437 | 2.0458 | 5.8488 | 0.0000 | 0.0010 | -0.8705 |
| MRPS9 | 1.1017 | 3.2515 | 2.1059 | 4.3971 | 1.7237 | 9.9302 | 0.0000 | 0.0000 | 7.0174 |
| POF1B | 1.0954 | -2.3661 | -3.3663 | -1.3660 | 2.1573 | -3.9897 | 0.0008 | 0.0364 | -5.0620 |
| SLC52A2 | 1.0893 | 3.1790 | 0.7758 | 5.5822 | 1.3065 | 15.2243 | 0.0000 | 0.0000 | 14.5927 |
| RNASET2 | 1.0773 | 3.7067 | 2.4058 | 5.0075 | 2.6784 | 9.2429 | 0.0000 | 0.0000 | 5.8394 |
| CYP2B6 | 1.0750 | 2.4770 | 1.4332 | 3.5208 | 1.1614 | 67.5004 | 0.0000 | 0.0000 | 43.5798 |
| PLEKHJ1 | 1.0671 | 3.0327 | 2.6685 | 3.3969 | 2.5893 | 4.1738 | 0.0005 | 0.0256 | -4.6269 |
| BZW2 | 1.0670 | 2.0479 | -0.0811 | 4.1769 | 0.9947 | 4.7768 | 0.0001 | 0.0082 | -3.1352 |
| TGFBR2 | 1.0630 | -2.2035 | -2.6346 | -1.7724 | 1.7201 | -5.2029 | 0.0001 | 0.0035 | -2.3021 |
| FERMT1 | 1.0532 | 2.3751 | 1.9792 | 2.7710 | 2.1458 | 4.2555 | 0.0004 | 0.0225 | -4.4332 |
| ARRDC1 | 1.0499 | 2.6005 | 1.7567 | 3.4444 | 1.3426 | 12.0907 | 0.0000 | 0.0000 | 10.4709 |
| ECHDC1 | 1.0441 | 2.8696 | 2.4387 | 3.3005 | 1.4219 | 20.3706 | 0.0000 | 0.0000 | 19.9600 |
| ANP32A | 1.0372 | 2.3211 | 1.9144 | 2.7278 | 2.9935 | 4.4302 | 0.0003 | 0.0163 | -4.0404 |
| TRNY | 1.0179 | 3.7472 | 2.3381 | 5.1563 | 2.2501 | 17.3794 | 0.0000 | 0.0000 | 16.8581 |
| CPEB4 | 1.0163 | 2.0411 | 1.8091 | 2.2731 | 1.5343 | 5.3280 | 0.0000 | 0.0027 | -2.0079 |
| RNPS1 | 1.0133 | -2.6085 | -2.8705 | -2.3465 | 1.9714 | -7.4011 | 0.0000 | 0.0001 | 2.3921 |
| GSTM4 | 1.0080 | 2.0349 | 1.5851 | 2.4846 | 1.1422 | 7.5343 | 0.0000 | 0.0000 | 2.7489 |
| MED31 | 1.0055 | 2.7576 | 2.1019 | 3.4133 | 1.2133 | 13.3427 | 0.0000 | 0.0000 | 12.1944 |

**D) Nancy**

| Sample | VIP Scores | logFC | CI.L | CI.R | AveExpr | t | P.Value | adj.P.Val | B |
| --- | --- | --- | --- | --- | --- | --- | --- | --- | --- |
| PDZK1IP1 | 1.7273 | -5.6081 | -6.0322 | -5.1840 | 4.0069 | -20.0512 | 0.0000 | 0.0000 | 16.8621 |
| DCAF6 | 1.6463 | 2.0701 | 1.4830 | 2.6571 | 0.5992 | 5.0758 | 0.0001 | 0.0012 | 0.9359 |
| RNASET2 | 1.5755 | 4.2496 | 3.9138 | 4.5855 | 2.6784 | 20.5675 | 0.0000 | 0.0000 | 17.3105 |
| USP2 | 1.5208 | 3.0637 | 0.4853 | 5.6421 | 1.3222 | 170.4111 | 0.0000 | 0.0000 | 58.9512 |
| DNMBP | 1.4582 | 2.3262 | -1.0507 | 5.7032 | 0.5761 | 65.6334 | 0.0000 | 0.0000 | 39.6447 |
| LCN2 | 1.4196 | -4.8817 | -6.8830 | -2.8804 | 4.9245 | -9.2096 | 0.0000 | 0.0000 | 3.3467 |
| VSIG2 | 1.4127 | 3.5998 | 2.0043 | 5.1953 | 2.0074 | 5.6394 | 0.0000 | 0.0004 | -3.7252 |
| BTN2A1 | 1.4125 | 2.5898 | 2.2874 | 2.8922 | 0.9022 | 433.8996 | 0.0000 | 0.0000 | 77.1324 |
| DHRSX_1 | 1.4121 | 3.2583 | 2.7756 | 3.7409 | 1.6589 | 10.5788 | 0.0000 | 0.0000 | 5.5019 |
| TRPT1 | 1.3986 | 3.6472 | 1.7014 | 5.5929 | 1.7188 | 7.6402 | 0.0000 | 0.0000 | 0.4020 |
| PDE8A | 1.3969 | 2.2718 | 0.9767 | 3.5669 | 0.8967 | 11.1635 | 0.0000 | 0.0000 | 6.5355 |
| MAFG | 1.3923 | 2.2282 | 1.8197 | 2.6367 | 0.5986 | 42.2439 | 0.0000 | 0.0000 | 34.2991 |
| YOD1 | 1.3890 | 2.2517 | 1.4000 | 3.1034 | 0.6303 | 22.0838 | 0.0000 | 0.0000 | 23.7258 |
| DUOX2 | 1.3704 | -3.3303 | -3.6837 | -2.9768 | 2.1215 | -11.6090 | 0.0000 | 0.0000 | 7.0808 |
| MED31 | 1.3430 | 3.3156 | 1.3360 | 5.2952 | 1.2133 | 242.3434 | 0.0000 | 0.0000 | 65.6884 |
| C7orf55-LUC7L2 | 1.3348 | 2.9734 | 2.6027 | 3.3440 | 0.8535 | 973.1416 | 0.0000 | 0.0000 | 92.4368 |
| FERMT1 | 1.3336 | 2.5667 | 1.9339 | 3.1995 | 2.1458 | 3.4229 | 0.0030 | 0.0356 | -8.7017 |
| CCZ1 | 1.3321 | 2.6837 | 0.4160 | 4.9513 | 1.0210 | 245.9757 | 0.0000 | 0.0000 | 66.2126 |
| VSIG10 | 1.3314 | 2.3641 | 1.9811 | 2.7471 | 1.2704 | 10.5877 | 0.0000 | 0.0000 | 5.5838 |
| OXSR1 | 1.3221 | 2.5389 | 0.2924 | 4.7853 | 1.1617 | 8.8911 | 0.0000 | 0.0000 | 2.7419 |
| EDN3 | 1.3203 | 2.8884 | 1.7019 | 4.0749 | 0.7464 | 205.1310 | 0.0000 | 0.0000 | 64.4679 |
| FNTA | 1.2957 | 3.8008 | 3.1889 | 4.4127 | 1.5439 | 27.9498 | 0.0000 | 0.0000 | 23.2208 |
| CPTP | 1.2920 | 2.9198 | 1.7178 | 4.1219 | 0.9375 | 123.0577 | 0.0000 | 0.0000 | 53.8137 |
| PIM2 | 1.2917 | -2.7106 | -3.3766 | -2.0445 | 3.2792 | -4.0906 | 0.0007 | 0.0092 | -7.2086 |
| RCOR1 | 1.2846 | 2.2074 | 0.7661 | 3.6488 | 0.6954 | 22.0685 | 0.0000 | 0.0000 | 18.8777 |
| TCEANC2 | 1.2825 | 2.1141 | 1.5361 | 2.6921 | 0.6407 | 30.3227 | 0.0000 | 0.0000 | 24.8894 |
| GLRX5 | 1.2808 | 4.2810 | 3.7582 | 4.8038 | 1.9266 | 10.8255 | 0.0000 | 0.0000 | 5.9636 |
| ASL | 1.2786 | 3.5881 | 3.2051 | 3.9711 | 1.6101 | 9.2534 | 0.0000 | 0.0000 | 3.3591 |
| FRA10AC1 | 1.2648 | 2.5976 | 1.9326 | 3.2625 | 1.7842 | 4.4900 | 0.0003 | 0.0040 | -6.2912 |
| TUFM | 1.2525 | 3.0342 | 1.7396 | 4.3288 | 2.1252 | 8.2238 | 0.0000 | 0.0000 | 1.4875 |
| MRPL1 | 1.2511 | 2.7599 | 1.4640 | 4.0558 | 1.3047 | 5.7850 | 0.0000 | 0.0003 | -3.3383 |
| RMDN2 | 1.2339 | 2.4089 | 1.4345 | 3.3834 | 0.8619 | 58.3242 | 0.0000 | 0.0000 | 37.2470 |
| CPEB4 | 1.2293 | 2.1705 | 1.5902 | 2.7507 | 1.5343 | 4.4071 | 0.0003 | 0.0048 | -6.4857 |
| AKAP1 | 1.2229 | 2.9960 | 2.4916 | 3.5004 | 1.4370 | 9.7392 | 0.0000 | 0.0000 | 4.2017 |
| MCU | 1.2143 | 2.7294 | 1.4821 | 3.9768 | 1.1960 | 48.0224 | 0.0000 | 0.0000 | 33.7099 |
| MT1H | 1.2122 | 3.6958 | 2.1956 | 5.1960 | 1.7380 | 5.0774 | 0.0001 | 0.0012 | -4.8910 |
| SFXN4 | 1.2023 | 2.1668 | 1.6619 | 2.6718 | 1.2492 | 6.0160 | 0.0000 | 0.0002 | -2.8250 |
| PCK1 | 1.1902 | 2.6055 | -0.8055 | 6.0164 | 1.4977 | 8.1662 | 0.0000 | 0.0000 | 1.3720 |
| PET117 | 1.1881 | 2.9599 | 1.9481 | 3.9718 | 1.8581 | 6.0789 | 0.0000 | 0.0002 | -2.7876 |
| PSMF1 | 1.1853 | 2.1150 | 1.6125 | 2.6176 | 1.1005 | 13.8186 | 0.0000 | 0.0000 | 10.1487 |
| RNF11 | 1.1828 | 2.3324 | 1.6673 | 2.9975 | 1.0627 | 7.5281 | 0.0000 | 0.0000 | 0.4924 |
| DHRS11 | 1.1751 | 2.8325 | -0.0615 | 5.7264 | 2.2325 | 3.7041 | 0.0016 | 0.0201 | -8.0580 |
| SRSF2 | 1.1684 | 2.0035 | 2.0032 | 2.0038 | 0.9675 | 5.6974 | 0.0000 | 0.0003 | -3.3664 |
| ELAVL1 | 1.1626 | 2.1872 | 1.5955 | 2.7789 | 0.7991 | 16.4870 | 0.0000 | 0.0000 | 13.4132 |
| S100P | 1.1576 | -2.4065 | -4.4701 | -0.3430 | 5.3617 | -4.0870 | 0.0007 | 0.0093 | -7.1751 |
| ENPP4 | 1.1559 | 2.1792 | -0.1053 | 4.4638 | 1.0357 | 16.1586 | 0.0000 | 0.0000 | 13.0023 |
| SLC35B1 | 1.1437 | 2.7321 | 1.0386 | 4.4255 | 0.9902 | 22.5417 | 0.0000 | 0.0000 | 22.5916 |
| FZD5 | 1.1430 | 2.0749 | 1.6609 | 2.4890 | 1.3060 | 4.8795 | 0.0001 | 0.0018 | -5.3951 |
| MGAT4B | 1.1420 | 2.0966 | 1.6437 | 2.5495 | 0.8185 | 13.6821 | 0.0000 | 0.0000 | 10.0646 |
| CAPN7 | 1.1338 | 2.3411 | 2.0617 | 2.6205 | 1.0918 | 13.3302 | 0.0000 | 0.0000 | 9.7921 |
| PLA2G2A | 1.1283 | -2.4030 | -2.6788 | -2.1272 | 5.4456 | -4.4796 | 0.0003 | 0.0041 | -6.2619 |
| RNF4 | 1.1275 | 2.7052 | 1.9709 | 3.4394 | 0.9076 | 120.0257 | 0.0000 | 0.0000 | 53.4486 |
| EPS8L3 | 1.1234 | 2.8500 | 2.2538 | 3.4462 | 1.2794 | 11.7250 | 0.0000 | 0.0000 | 7.3538 |
| RCN2 | 1.1145 | 3.0969 | 1.7046 | 4.4892 | 1.4137 | 10.2254 | 0.0000 | 0.0000 | 5.1056 |
| GTF3A | 1.1042 | 3.1816 | 3.1790 | 3.1843 | 2.8585 | 3.6357 | 0.0018 | 0.0231 | -8.2211 |
| ANGEL2 | 1.1009 | 2.3204 | 1.7472 | 2.8936 | 0.8542 | 16.9447 | 0.0000 | 0.0000 | 18.0816 |
| PSMD7 | 1.1007 | -2.7661 | -3.0955 | -2.4367 | 3.9742 | -3.8827 | 0.0011 | 0.0141 | -7.6131 |
| FUCA2 | 1.0923 | 2.7280 | 2.2636 | 3.1925 | 1.6312 | 5.6010 | 0.0000 | 0.0004 | -3.7895 |
| SURF2 | 1.0907 | 2.7833 | 2.2766 | 3.2900 | 0.8689 | 14.2580 | 0.0000 | 0.0000 | 15.2276 |
| CCDC124 | 1.0863 | 3.4047 | 2.9852 | 3.8243 | 2.0379 | 5.1584 | 0.0001 | 0.0010 | -4.7620 |
| HSPA14 | 1.0843 | 2.5854 | 1.5400 | 3.6308 | 0.8538 | 5.8036 | 0.0000 | 0.0003 | 2.0626 |
| MMP24OS | 1.0780 | 3.5300 | 2.7514 | 4.3085 | 1.9620 | 6.3654 | 0.0000 | 0.0001 | -2.1263 |
| FIS1 | 1.0705 | 3.1597 | 2.6769 | 3.6426 | 3.3136 | 4.1786 | 0.0005 | 0.0077 | -6.9897 |
| ZFYVE21 | 1.0694 | 2.9724 | 2.9719 | 2.9728 | 1.3176 | 8.6943 | 0.0000 | 0.0000 | 2.4335 |
| MRPS9 | 1.0665 | 3.0449 | 2.4187 | 3.6710 | 1.7237 | 7.1945 | 0.0000 | 0.0000 | -0.4149 |
| CRACR2B | 1.0648 | 2.2826 | 0.1856 | 4.3795 | 0.6627 | 57.3027 | 0.0000 | 0.0000 | 41.2849 |
| PKP3 | 1.0641 | 2.2427 | 1.9489 | 2.5366 | 1.6579 | 3.9663 | 0.0009 | 0.0118 | -7.4773 |
| ESCO1 | 1.0636 | 2.4109 | 1.5899 | 3.2319 | 0.9892 | 21.2240 | 0.0000 | 0.0000 | 18.1209 |
| MYO1B | 1.0571 | 2.0618 | 1.4702 | 2.6534 | 1.2126 | 5.0523 | 0.0001 | 0.0013 | -4.9247 |
| SULT1A1 | 1.0555 | 2.0167 | 0.3630 | 3.6704 | 0.8165 | 4.8906 | 0.0001 | 0.0017 | 0.1893 |
| KNOP1 | 1.0508 | 3.5551 | 0.2299 | 6.8802 | 1.0753 | 29.3189 | 0.0000 | 0.0000 | 27.2852 |
| QTRT1 | 1.0506 | 3.4430 | 1.8883 | 4.9977 | 1.9778 | 6.8593 | 0.0000 | 0.0000 | -1.1261 |
| VAMP3 | 1.0426 | 2.2302 | 1.6028 | 2.8576 | 1.2424 | 5.7398 | 0.0000 | 0.0003 | -3.4606 |
| SCYL2 | 1.0392 | 2.1886 | 2.1880 | 2.1893 | 1.2277 | 5.7712 | 0.0000 | 0.0003 | -3.3441 |
| EHHADH | 1.0338 | 2.2234 | 1.5675 | 2.8793 | 0.7636 | 53.9918 | 0.0000 | 0.0000 | 35.8960 |
| CRYL1 | 1.0257 | 2.6429 | 2.0176 | 3.2683 | 1.4027 | 8.4477 | 0.0000 | 0.0000 | 2.0003 |
| RSBN1L | 1.0215 | 2.4241 | 2.1042 | 2.7439 | 1.3204 | 7.8273 | 0.0000 | 0.0000 | 0.8242 |
| TMEM97 | 1.0201 | 3.3062 | 1.5581 | 5.0543 | 1.0319 | 95.0503 | 0.0000 | 0.0000 | 48.8580 |
| SDHAF3 | 1.0184 | 2.4746 | 0.1704 | 4.7788 | 0.9237 | 7.0288 | 0.0000 | 0.0000 | -0.6088 |
| CARNMT1 | 1.0178 | 2.3958 | 1.6011 | 3.1906 | 0.8824 | 24.6258 | 0.0000 | 0.0000 | 20.8106 |
| TTC19 | 1.0143 | 2.0101 | 1.5575 | 2.4626 | 1.3947 | 3.3870 | 0.0032 | 0.0383 | -8.7494 |
| HOXB9 | 1.0109 | 2.6825 | 2.0006 | 3.3644 | 1.3291 | 56.5326 | 0.0000 | 0.0000 | 36.9611 |
| SLAIN2 | 1.0045 | 2.5033 | 0.5176 | 4.4890 | 1.6550 | 4.6920 | 0.0002 | 0.0026 | -5.7723 |
