# Supplementary material for: Ultra-high Magnification Endocytoscopy and Molecular Markers for Defining Endoscopic and Histologic Remission in Ulcerative Colitis—An Exploratory Study to Define Deep Remission
Source: Inflamm Bowel Dis. 2021 May 21;27(11):1719–30. doi: 10.1093/ibd/izab059 (PMC8528147; doi:10.1093/ibd/izab059)
Supplement: izab059_suppl_Supplementary_Table_5 [file izab059_suppl_supplementary_table_5.docx]

**Supplementary Table 5:** KEGG pathway enrichment analysis of the differentially expressed genes defined by A) ECSS, B) Mayo, C) RHI and D) Nancy scores. Genes in pathways for which p<0.05 are listed.

1. **ECSS**

| **Pathway** | **P-value** | **Adjusted P-value** | **Genes** |
| --- | --- | --- | --- |
| Glyoxylate and dicarboxylate metabolism | 9.58E-04 | 0.295123447 | PCCB;ACO2;ACAT1 |
| Spliceosome | 0.001837 | 0.282872521 | DHX8;ZMAT2;PLRG1;TXNL4A;PQBP1 |
| Terpenoid backbone biosynthesis | 0.008895 | 0.913199567 | FNTA;ACAT1 |
| Citrate cycle (TCA cycle) | 0.016193 | 1 | ACO2;PCK1 |
| Mucin type O-glycan biosynthesis | 0.017237 | 1 | GCNT3;B4GALT5 |
| Propanoate metabolism | 0.018309 | 0.939864758 | PCCB;ACAT1 |
| Pyruvate metabolism | 0.02656 | 1 | PCK1;ACAT1 |
| Th17 cell differentiation | 0.032663 | 1 | STAT6;AHR;TGFBR2 |
| Valine, leucine and isoleucine degradation | 0.038936 | 1 | PCCB;ACAT1 |
| N-Glycan biosynthesis | 0.041932 | 1 | DPM2;ALG3 |

**B) Mayo**

| Pathway | P-value | Adjusted P-value | Genes |
| --- | --- | --- | --- |
| Mineral absorption | 1.84E-05 | 0.005673 | MT2A;MT1F;MT1G;MT1X;MT1H |
| Steroid hormone biosynthesis | 5.92E-04 | 0.091101 | HSD11B2;DHRS11;HSD17B2;COMT |
| Citrate cycle (TCA cycle) | 9.16E-04 | 0.094059 | CS;IDH3G;PCK1 |
| Endocytosis | 9.99E-04 | 0.076891 | RAB10;SNX2;BIN1;VPS4A;WASL;VPS28;RAB11B |
| AMPK signaling pathway | 0.001051 | 0.06477 | RAB10;PCK1;ELAVL1;CFTR;RAB11B |
| Tryptophan metabolism | 0.00245 | 0.125754 | ALDH3A2;EHHADH;CAT |
| Fatty acid degradation | 0.0028 | 0.123202 | HADHB;ALDH3A2;EHHADH |
| IL-17 signaling pathway | 0.003012 | 0.11597 | JUN;LCN2;CXCL1;ELAVL1 |
| Valine, leucine and isoleucine degradation | 0.003589 | 0.122838 | HADHB;ALDH3A2;EHHADH |
| Bile secretion | 0.011077 | 0.341178 | PRKACB;CFTR;ABCG2 |
| Thyroid hormone synthesis | 0.01193 | 0.334031 | DUOXA2;PRKACB;DUOX2 |
| Glyoxylate and dicarboxylate metabolism | 0.015726 | 0.403633 | CS;CAT |
| beta-Alanine metabolism | 0.016741 | 0.39663 | ALDH3A2;EHHADH |
| Salmonella infection | 0.017823 | 0.392115 | JUN;CXCL1;WASL |
| Fc gamma R-mediated phagocytosis | 0.020678 | 0.424597 | GSN;BIN1;WASL |
| Tight junction | 0.023838 | 0.458886 | JUN;WASL;PRKACB;CFTR |
| Pyruvate metabolism | 0.025808 | 0.467583 | ALDH3A2;PCK1 |
| Vasopressin-regulated water reabsorption | 0.032275 | 0.552264 | PRKACB;RAB11B |
| ABC transporters | 0.033637 | 0.545281 | CFTR;ABCG2 |
| Cocaine addiction | 0.039305 | 0.605296 | JUN;PRKACB |
| Ovarian steroidogenesis | 0.039305 | 0.576472 | HSD17B2;PRKACB |
| Viral carcinogenesis | 0.040367 | 0.565131 | JUN;GSN;GTF2B;PRKACB |
| Vibrio cholerae infection | 0.040775 | 0.546026 | PRKACB;CFTR |

**C) RHI**

| Pathways | P-value | Adjusted P-value | Genes |
| --- | --- | --- | --- |
| Vasopressin-regulated water reabsorption | 0.014379 | 1 | DCTN6;DYNC1LI2 |
| Purine metabolism | 0.016561 | 1 | AK2;ENPP4;NUDT16 |
| Metabolism of xenobiotics by cytochrome P450 | 0.037909 | 1 | GSTM4;CYP2B6 |
| NOD-like receptor signaling pathway | 0.037942 | 1 | CASP5;STAT1;MCU |
| Pancreatic cancer | 0.038845 | 1 | STAT1;TGFBR2 |

**D) Nancy**

| **Pathway** | **P-value** | **Adjusted P-value** | **Genes** |
| --- | --- | --- | --- |
| N-Glycan biosynthesis | 0.001421 | 0.437816 | DPM2;MGAT4B;ALG3;MGAT1 |
| RNA polymerase | 0.003355 | 0.516732 | POLR3GL;POLR1C;POLR2G |
| Propanoate metabolism | 0.003676 | 0.377422 | EHHADH;ECHDC1;ACAT1 |
| Arginine biosynthesis | 0.017584 | 1 | GOT1;ASL |
| Terpenoid backbone biosynthesis | 0.01922 | 1 | FNTA;ACAT1 |
| Other types of O-glycan biosynthesis | 0.01922 | 0.986635 | POGLUT1;COLGALT1 |
| Lysine degradation | 0.019899 | 0.875552 | EHHADH;COLGALT1;ACAT1 |
| Butanoate metabolism | 0.030277 | 1 | EHHADH;ACAT1 |
| Citrate cycle (TCA cycle) | 0.034405 | 1 | ACO2;PCK1 |
| Glyoxylate and dicarboxylate metabolism | 0.034405 | 1 | ACO2;ACAT1 |
| Metabolism of xenobiotics by cytochrome P450 | 0.035718 | 1 | GSTM4;CYP2B6;UGT2A3 |
| PPAR signaling pathway | 0.035718 | 0.916754 | EHHADH;PCK1;SLC27A2 |
| Spliceosome | 0.042342 | 1 | DHX8;SRSF2;TXNL4A;PQBP1 |
| Pentose and glucuronate interconversions | 0.043266 | 0.951852 | UGT2A3;CRYL1 |
| Alanine, aspartate and glutamate metabolism | 0.0456 | 0.936329 | GOT1;ASL |
| Chemical carcinogenesis | 0.04615 | 0.888382 | SULT1A1;GSTM4;UGT2A3 |
| Phenylalanine, tyrosine and tryptophan biosynthesis | 0.047813 | 0.86626 | GOT1 |
