# Supplementary material for: Ultra-high Magnification Endocytoscopy and Molecular Markers for Defining Endoscopic and Histologic Remission in Ulcerative Colitis—An Exploratory Study to Define Deep Remission
Source: Inflamm Bowel Dis. 2021 May 21;27(11):1719–30. doi: 10.1093/ibd/izab059 (PMC8528147; doi:10.1093/ibd/izab059)
Supplement: izab059_suppl_Supplementary_Table_6 [file izab059_suppl_supplementary_table_6.docx]

**Supplementary Table 6:** Go Biological process enrichment analysis of the differentially expressed genes defined by A) ECSS, B) Mayo, C) RHI and D) Nancy scores. Genes in pathways for which p<0.05 are listed.

**A) ECSS**

| **Biological Process** | **P-value** | **Adjusted P-value** | **Genes** |
| --- | --- | --- | --- |
| positive regulation of type I interferon production (GO:0032481) | 7.10E-04 | 1 | POLR3GL;FLOT1;STAT6;PQBP1 |
| short-chain fatty acid catabolic process (GO:0019626) | 0.001144232 | 1 | PCCB;PCK1 |
| response to sterol (GO:0036314) | 0.002219058 | 1 | INSIG1;TGFBR2 |
| negative regulation of substrate adhesion-dependent cell spreading (GO:1900025) | 0.002651561 | 1 | AP1AR;FBLN1 |
| cellular response to exogenous dsRNA (GO:0071360) | 0.003120361 | 1 | FLOT1;PQBP1 |
| endosome to lysosome transport (GO:0008333) | 0.00374914 | 1 | SNAPIN;BIN1;SCYL2 |
| RNA splicing, via transesterification reactions with bulged adenosine as nucleophile (GO:0000377) | 0.004484009 | 1 | DHX8;ZMAT2;GEMIN6;PLRG1;TXNL4A;PQBP1 |
| cellular response to dsRNA (GO:0071359) | 0.00473974 | 1 | FLOT1;PQBP1 |
| protein O-linked glycosylation (GO:0006493) | 0.006761228 | 1 | DPM2;POGLUT1;GCNT3;B4GALT5 |
| mRNA splicing, via spliceosome (GO:0000398) | 0.00724515 | 1 | DHX8;ZMAT2;GEMIN6;PLRG1;TXNL4A;PQBP1 |
| melanosome organization (GO:0032438) | 0.009700777 | 1 | RAB32;SNAPIN |
| protein glycosylation (GO:0006486) | 0.010315151 | 1 | POGLUT1;ALG3;GCNT3;SIRT6 |
| mRNA processing (GO:0006397) | 0.010547371 | 1 | DHX8;ZMAT2;GEMIN6;PLRG1;TXNL4A;PQBP1 |
| lysosomal transport (GO:0007041) | 0.011549909 | 1 | SNAPIN;BIN1;SCYL2 |
| canonical Wnt signaling pathway (GO:0060070) | 0.011988693 | 1 | HOXB9;FZD5;SCYL2 |
| transcription from RNA polymerase III promoter (GO:0006383) | 0.013232733 | 1 | POLR3GL;GTF3A |
| cellular response to ketone (GO:1901655) | 0.014190598 | 1 | AHR;PCK1 |
| RNA phosphodiester bond hydrolysis (GO:0090501) | 0.014190598 | 1 | LACTB2;RNASE6 |
| regulation of receptor internalization (GO:0002090) | 0.014190598 | 1 | FLOT1;SCYL2 |
| positive regulation of cytokine production (GO:0001819) | 0.014542657 | 1 | FZD5;POLR3GL;FLOT1;STAT6;PQBP1 |
| regulation of embryonic development (GO:0045995) | 0.015177573 | 1 | POGLUT1;RBM19 |
| negative regulation of cell morphogenesis involved in differentiation (GO:0010771) | 0.016193246 | 1 | FBLN1;AP1AR |
| protein stabilization (GO:0050821) | 0.017417574 | 1 | TNIP2;USP2;FLOT1;CCT8 |
| regulation of type I interferon production (GO:0032479) | 0.01800139 | 1 | POLR3GL;STAT6;PQBP1 |
| response to exogenous dsRNA (GO:0043330) | 0.019408383 | 1 | FLOT1;PQBP1 |
| Wnt signaling pathway, planar cell polarity pathway (GO:0060071) | 0.019722525 | 1 | DAAM1;FZD5;PSMD3 |
| regulation of establishment of planar polarity (GO:0090175) | 0.020315749 | 1 | DAAM1;FZD5;PSMD3 |
| regulation of interleukin-1 beta secretion (GO:0050706) | 0.020534799 | 1 | FZD5;CPTP |
| negative regulation of cell-substrate adhesion (GO:0010812) | 0.021687909 | 1 | FBLN1;AP1AR |
| positive regulation of cellular metabolic process (GO:0031325) | 0.022154034 | 1 | APOC1;SCYL2;TGFBR2 |
| regulation of mitotic cell cycle (GO:0007346) | 0.022763 | 1 | ANGEL2;USP2;HECA;OXSR1 |
| regulation of receptor-mediated endocytosis (GO:0048259) | 0.022867323 | 1 | APOC1;FLOT1 |
| cellular response to organonitrogen compound (GO:0071417) | 0.025413301 | 1 | DHX8;AHR;CPEB4 |
| response to cAMP (GO:0051591) | 0.027840386 | 1 | AHR;DUOX2 |
| tRNA aminoacylation (GO:0043039) | 0.027840386 | 1 | AIMP2;EEF1E1 |
| regulation of substrate adhesion-dependent cell spreading (GO:1900024) | 0.029145624 | 1 | FBLN1;AP1AR |
| RNA catabolic process (GO:0006401) | 0.031827893 | 1 | RNASET2;RNASE6 |
| nucleobase-containing compound catabolic process (GO:0034655) | 0.031827893 | 1 | RNASET2;RNASE6 |
| negative regulation of protein modification by small protein conjugation or removal (GO:1903321) | 0.036025388 | 1 | SVBP;RNF4 |
| tRNA aminoacylation for protein translation (GO:0006418) | 0.036025388 | 1 | AIMP2;EEF1E1 |
| positive regulation of tumor necrosis factor production (GO:0032760) | 0.037469567 | 1 | FZD5;LY96 |
| negative regulation of mRNA catabolic process (GO:1902373) | 0.038376226 | 1 | PKP3 |
| arginine biosynthetic process (GO:0006526) | 0.038376226 | 1 | ASL |
| protein geranylgeranylation (GO:0018344) | 0.038376226 | 1 | FNTA |
| positive regulation of deacetylase activity (GO:0090045) | 0.038376226 | 1 | FNTA |
| positive regulation of toll-like receptor 3 signaling pathway (GO:0034141) | 0.038376226 | 1 | FLOT1 |
| ribonucleoside biosynthetic process (GO:0042455) | 0.038376226 | 1 | QTRT2 |
| negative regulation of cytoplasmic translation (GO:2000766) | 0.038376226 | 1 | CPEB4 |
| viral release from host cell (GO:0019076) | 0.038376226 | 1 | PPID |
| regulation of T cell tolerance induction (GO:0002664) | 0.038376226 | 1 | TGFBR2 |
| adenylate cyclase-inhibiting G-protein coupled acetylcholine receptor signaling pathway (GO:0007197) | 0.038376226 | 1 | DHX8 |
| SREBP signaling pathway (GO:0032933) | 0.038376226 | 1 | INSIG1 |
| ceramide transport (GO:0035627) | 0.038376226 | 1 | CPTP |
| muscle tissue development (GO:0060537) | 0.038376226 | 1 | POGLUT1 |
| positive regulation of clathrin-dependent endocytosis (GO:2000370) | 0.038376226 | 1 | SCYL2 |
| negative regulation of sterol transport (GO:0032372) | 0.038376226 | 1 | APOC1 |
| negative regulation of phospholipid metabolic process (GO:1903726) | 0.038376226 | 1 | APOC1 |
| queuosine biosynthetic process (GO:0008616) | 0.038376226 | 1 | QTRT2 |
| exit from host cell (GO:0035891) | 0.038376226 | 1 | PPID |
| regulation of epithelial to mesenchymal transition involved in endocardial cushion formation (GO:1905005) | 0.038376226 | 1 | TGFBR2 |
| queuosine metabolic process (GO:0046116) | 0.038376226 | 1 | QTRT2 |
| membrane raft assembly (GO:0001765) | 0.038376226 | 1 | FLOT1 |
| citrate metabolic process (GO:0006101) | 0.038376226 | 1 | ACO2 |
| response to UV-A (GO:0070141) | 0.038376226 | 1 | PPID |
| acetyl-CoA biosynthetic process (GO:0006085) | 0.038376226 | 1 | ACAT1 |
| negative regulation of mitotic cell cycle (GO:0045930) | 0.038935675 | 1 | ANGEL2;HECA |
| cellular macromolecule catabolic process (GO:0044265) | 0.038935675 | 1 | RNASET2;RNASE6 |
| cellular lipid biosynthetic process (GO:0097384) | 0.044628639 | 1 | ACAT1 |
| positive regulation of inositol phosphate biosynthetic process (GO:0060732) | 0.044628639 | 1 | DHX8 |
| coenzyme A biosynthetic process (GO:0015937) | 0.044628639 | 1 | ACAT1 |
| regulation of alpha-beta T cell differentiation (GO:0046637) | 0.044628639 | 1 | PRDM1 |
| negative regulation of MyD88-independent toll-like receptor signaling pathway (GO:0034128) | 0.044628639 | 1 | LY96 |
| ribonucleoside bisphosphate biosynthetic process (GO:0034030) | 0.044628639 | 1 | ACAT1 |
| positive regulation of cellular senescence (GO:2000774) | 0.044628639 | 1 | EEF1E1 |
| regulation of toll-like receptor 3 signaling pathway (GO:0034139) | 0.044628639 | 1 | FLOT1 |
| cellular response to histamine (GO:0071420) | 0.044628639 | 1 | DHX8 |
| cellular response to sterol depletion (GO:0071501) | 0.044628639 | 1 | INSIG1 |
| regulation of sterol transport (GO:0032371) | 0.044628639 | 1 | APOC1 |
| purine nucleoside bisphosphate biosynthetic process (GO:0034033) | 0.044628639 | 1 | ACAT1 |
| positive regulation of B cell differentiation (GO:0045579) | 0.044628639 | 1 | PPP2R3C |
| chylomicron remnant clearance (GO:0034382) | 0.044628639 | 1 | APOC1 |
| detection of molecule of bacterial origin (GO:0032490) | 0.044628639 | 1 | LY96 |
| positive regulation of CD4-positive, alpha-beta T cell activation (GO:2000516) | 0.044628639 | 1 | TGFBR2 |
| negative regulation of nitrogen compound metabolic process (GO:0051172) | 0.044628639 | 1 | APOC1 |

**B) Mayo**

| **Biological Process** | **P-value** | **Adjusted**  **P-value** | **Genes** |
| --- | --- | --- | --- |
| cellular response to cadmium ion (GO:0071276) | 2.57E-08 | 1.31E-04 | MT2A;JUN;MT1F;MT1G;MT1X;MT1H |
| response to cadmium ion (GO:0046686) | 3.94E-08 | 1.01E-04 | MT2A;JUN;MT1F;MT1G;MT1X;MT1H |
| cellular response to zinc ion (GO:0071294) | 1.07E-07 | 1.83E-04 | MT2A;MT1F;MT1G;MT1X;MT1H |
| cellular response to copper ion (GO:0071280) | 1.86E-07 | 2.37E-04 | MT2A;MT1F;MT1G;MT1X;MT1H |
| response to copper ion (GO:0046688) | 4.76E-07 | 4.86E-04 | MT2A;MT1F;MT1G;MT1X;MT1H |
| cellular zinc ion homeostasis (GO:0006882) | 1.48E-06 | 0.001255118 | MT2A;MT1F;MT1G;MT1X;MT1H |
| zinc ion homeostasis (GO:0055069) | 1.48E-06 | 0.001075816 | MT2A;MT1F;MT1G;MT1X;MT1H |
| response to zinc ion (GO:0010043) | 1.74E-06 | 0.00110996 | MT2A;MT1F;MT1G;MT1X;MT1H |
| cellular transition metal ion homeostasis (GO:0046916) | 2.24E-06 | 0.001268639 | MT2A;MT1F;LCN2;MT1G;MT1X;MT1H;ABCG2 |
| vesicle-mediated transport (GO:0016192) | 1.31E-05 | 0.006675874 | RAB10;TMEM87B;SNX2;BIN1;VPS4A;PLIN3;WASL;OSBPL1A;SFT2D1;PICALM;STX10;RAB11B |
| endosomal transport (GO:0016197) | 1.69E-05 | 0.007851896 | RAB10;TMEM87B;SNX2;BIN1;VPS4A;VPS28;PICALM;STX10;RAB11B |
| cellular response to metal ion (GO:0071248) | 4.45E-05 | 0.01893684 | MT2A;JUN;MT1F;MT1G;MT1X;MT1H |
| protein acetylation (GO:0006473) | 5.67E-05 | 0.022264506 | NAA60;GTF2B;EHHADH;PCK1 |
| positive regulation of viral transcription (GO:0050434) | 1.35E-04 | 0.049180572 | JUN;DHX9;POLR2B;GTF2B |
| positive regulation of viral process (GO:0048524) | 1.78E-04 | 0.060625607 | DHX9;POLR2B;GTF2B;VPS4A |
| neutrophil degranulation (GO:0043312) | 2.60E-04 | 0.082997743 | RAB10;DPP7;GSN;JUP;RNASET2;CREG1;FUCA2;CAT;LCN2;HBB;CXCL1 |
| neutrophil activation involved in immune response (GO:0002283) | 2.79E-04 | 0.083857365 | RAB10;DPP7;GSN;JUP;RNASET2;CREG1;FUCA2;CAT;LCN2;HBB;CXCL1 |
| neutrophil mediated immunity (GO:0002446) | 3.00E-04 | 0.084954686 | RAB10;DPP7;GSN;JUP;RNASET2;CREG1;FUCA2;CAT;LCN2;HBB;CXCL1 |
| cellular divalent inorganic cation homeostasis (GO:0072503) | 4.81E-04 | 0.129259793 | MT2A;MT1F;MT1G;MT1X;MT1H |
| positive regulation of clathrin-dependent endocytosis (GO:2000370) | 5.99E-04 | 0.152947999 | WASL;PICALM |
| regulation of mRNA processing (GO:0050684) | 7.46E-04 | 0.181320552 | PABPN1;DHX9;SAFB2 |
| negative regulation of growth (GO:0045926) | 0.001051464 | 0.243891878 | MT2A;MT1F;MT1G;MT1X;MT1H |
| ribosome disassembly (GO:0032790) | 0.001109606 | 0.246187762 | DENR;MTIF3 |
| regulation of aspartic-type endopeptidase activity involved in amyloid precursor protein catabolic process (GO:1902959) | 0.001109606 | 0.235929938 | BIN1;PICALM |
| vesicle organization (GO:0016050) | 0.001353759 | 0.276329201 | SNX2;VPS4A;WASL;PICALM;STX10 |
| steroid biosynthetic process (GO:0006694) | 0.001660179 | 0.325842034 | HSD11B2;DHRS11;HSD17B2;OSBPL1A |
| regulation of viral transcription (GO:0046782) | 0.002128021 | 0.402196027 | DHX9;POLR2B;GTF2B |
| response to cAMP (GO:0051591) | 0.002128021 | 0.387831883 | JUN;DUOX2;CFTR |
| regulation of stem cell population maintenance (GO:2000036) | 0.002152335 | 0.378736702 | CNOT1;ELAVL1 |
| internal protein amino acid acetylation (GO:0006475) | 0.002152335 | 0.366112145 | EHHADH;PCK1 |
| negative regulation of amyloid precursor protein catabolic process (GO:1902992) | 0.002152335 | 0.354302076 | BIN1;PICALM |
| transcytosis (GO:0045056) | 0.002152335 | 0.343230136 | PICALM;RAB11B |
| regulation of protein localization (GO:0032880) | 0.002285372 | 0.353401558 | VPS4A;STX10;PICALM |
| vesicle budding from membrane (GO:0006900) | 0.002572003 | 0.386027459 | VPS4A;WASL |
| peptidyl-methionine modification (GO:0018206) | 0.002572003 | 0.374998103 | NAA60;METAP1 |
| cholesterol transport (GO:0030301) | 0.002800039 | 0.396905549 | VPS4A;APOC1;ABCG2 |
| nucleus organization (GO:0006997) | 0.002986141 | 0.411845297 | BIN1;VPS4A;TMEM170A |
| renal absorption (GO:0070293) | 0.003026938 | 0.406485935 | GSN;HBB |
| endoplasmic reticulum tubular network organization (GO:0071786) | 0.003026938 | 0.396063218 | RAB10;TMEM170A |
| positive regulation of actin nucleation (GO:0051127) | 0.003026938 | 0.386161638 | GSN;WASL |
| ribonucleoprotein complex disassembly (GO:0032988) | 0.003026938 | 0.376743061 | DENR;MTIF3 |
| negative regulation of receptor-mediated endocytosis (GO:0048261) | 0.003516678 | 0.427276317 | APOC1;PICALM |
| hydrogen peroxide catabolic process (GO:0042744) | 0.003516678 | 0.417339659 | CAT;HBB |
| fatty acid oxidation (GO:0019395) | 0.004030023 | 0.467391037 | HADHB;ALDH3A2;EHHADH |
| fatty acid beta-oxidation (GO:0006635) | 0.004030023 | 0.45700457 | HADHB;EHHADH;ETFDH |
| regulation of mRNA metabolic process (GO:1903311) | 0.004040764 | 0.448261319 | DHX9;SAFB2 |
| regulation of clathrin-dependent endocytosis (GO:2000369) | 0.004040764 | 0.438723844 | WASL;PICALM |
| regulation of actin nucleation (GO:0051125) | 0.004598746 | 0.488904196 | GSN;WASL |
| cellular response to dsRNA (GO:0071359) | 0.004598746 | 0.47892656 | DHX9;RIOK3 |
| positive regulation of DNA-templated transcription, initiation (GO:2000144) | 0.004598746 | 0.469348028 | JUN;GTF2B |
| regulation of protein localization to membrane (GO:1905475) | 0.005190174 | 0.519322731 | GSN;VPS4A |
| response to purine-containing compound (GO:0014074) | 0.005190174 | 0.509335755 | JUN;DUOX2 |
| N-terminal protein amino acid modification (GO:0031365) | 0.005190174 | 0.499725647 | NAA60;METAP1 |
| positive regulation by host of viral transcription (GO:0043923) | 0.005190174 | 0.490471468 | JUN;GTF2B |
| regulation of amyloid-beta formation (GO:1902003) | 0.005814605 | 0.539489598 | BIN1;PICALM |
| ubiquitin-dependent protein catabolic process via the multivesicular body sorting pathway (GO:0043162) | 0.005814605 | 0.529855855 | VPS4A;VPS28 |
| response to organophosphorus (GO:0046683) | 0.005814605 | 0.520560138 | JUN;DUOX2 |
| response to reactive oxygen species (GO:0000302) | 0.006717739 | 0.591045216 | JUN;CAT;HBB |
| intracellular protein transport (GO:0006886) | 0.007039025 | 0.608816038 | RAB10;PDCD6;TLK1;VPS28;STX10;RAB11B;ARCN1 |
| hydrogen peroxide metabolic process (GO:0042743) | 0.007160717 | 0.609018991 | CAT;HBB |
| viral budding via host ESCRT complex (GO:0039702) | 0.007160717 | 0.599035073 | VPS4A;VPS28 |
| organelle disassembly (GO:1903008) | 0.007160717 | 0.589373217 | DENR;MTIF3 |
| iron ion homeostasis (GO:0055072) | 0.007688351 | 0.622756393 | LCN2;ABCG2;PICALM |
| fatty acid catabolic process (GO:0009062) | 0.008379038 | 0.668097356 | HADHB;ALDH3A2;EHHADH |
| regulation of gene silencing by miRNA (GO:0060964) | 0.009105084 | 0.71481917 | DHX9;POLR2B;ELAVL1 |
| cholesterol efflux (GO:0033344) | 0.009416541 | 0.728069855 | APOC1;ABCG2 |
| viral budding (GO:0046755) | 0.009416541 | 0.71720314 | VPS4A;VPS28 |
| response to metal ion (GO:0010038) | 0.011077212 | 0.831279583 | MT2A;PDCD6;MT1X |
| positive regulation of defense response (GO:0031349) | 0.011929681 | 0.882277675 | DHX9;DDT;RIOK3 |
| positive regulation of interferon-beta production (GO:0032728) | 0.011946217 | 0.870879207 | DHX9;RIOK3 |
| regulation of translation (GO:0006417) | 0.012032518 | 0.864816016 | DHX9;CNOT1;POLR2B;ELAVL1;METAP1 |
| response to lipid (GO:0033993) | 0.012578438 | 0.891496795 | JUN;HSD17B2;CXCL1;PCK1 |
| modulation by host of viral transcription (GO:0043921) | 0.012848369 | 0.898153825 | JUN;GTF2B |
| regulation of protein localization to cell surface (GO:2000008) | 0.012848369 | 0.886016611 | RAB11B;PICALM |
| cellular response to glucose stimulus (GO:0071333) | 0.012848369 | 0.874203056 | PCK1;RAB11B |
| negative regulation of endocytosis (GO:0045806) | 0.01377931 | 0.925208162 | APOC1;PICALM |
| cellular response to ketone (GO:1901655) | 0.01377931 | 0.913192472 | PCK1;CFTR |
| retrograde transport, endosome to Golgi (GO:0042147) | 0.015709267 | 1 | TMEM87B;SNX2;STX10 |
| regulation of leukocyte chemotaxis (GO:0002688) | 0.015725963 | 1 | CXCL1;IL6R |
| nuclear envelope organization (GO:0006998) | 0.015725963 | 1 | VPS4A;TMEM170A |
| multivesicular body assembly (GO:0036258) | 0.015725963 | 0.990735657 | VPS4A;VPS28 |
| multivesicular body organization (GO:0036257) | 0.016740887 | 1 | VPS4A;VPS28 |
| vesicle-mediated transport to the plasma membrane (GO:0098876) | 0.016740887 | 1 | RAB10;RAB11B |
| protein transport (GO:0015031) | 0.018547406 | 1 | RAB10;PDCD6;TLK1;STX10;RAB11B;ARCN1 |
| cellular protein localization (GO:0034613) | 0.01879808 | 1 | RAB10;PDCD6;TLK1;STX10;RAB11B;ARCN1 |
| positive regulation of nucleocytoplasmic transport (GO:0046824) | 0.018851993 | 1 | JUP;DHX9 |
| platelet aggregation (GO:0070527) | 0.018851993 | 1 | HBB;METAP1 |
| mRNA stabilization (GO:0048255) | 0.018851993 | 1 | DHX9;ELAVL1 |
| positive regulation of leukocyte migration (GO:0002687) | 0.019947409 | 1 | CXCL1;IL6R |
| positive regulation of exocytosis (GO:0045921) | 0.019947409 | 1 | VPS4A;CFTR |
| endoplasmic reticulum organization (GO:0007029) | 0.019947409 | 1 | RAB10;TMEM170A |
| cellular macromolecular complex assembly (GO:0034622) | 0.019947409 | 1 | BIN1;PICALM |
| negative regulation of protein kinase B signaling (GO:0051898) | 0.021068897 | 1 | PDCD6;NOP53 |
| negative regulation of protein complex assembly (GO:0031333) | 0.021068897 | 1 | CPTP;NOP53 |
| positive regulation of ATPase activity (GO:0032781) | 0.021068897 | 1 | DHX9;TOR1AIP1 |
| actin polymerization or depolymerization (GO:0008154) | 0.022216085 | 1 | GSN;WASL |
| regulation of receptor-mediated endocytosis (GO:0048259) | 0.022216085 | 1 | APOC1;PICALM |
| positive regulation of smooth muscle cell proliferation (GO:0048661) | 0.023388603 | 1 | JUN;IL6R |
| regulation of cell death (GO:0010941) | 0.023770051 | 1 | JUN;HBB;PDE8A |
| mitochondrial respiratory chain complex assembly (GO:0033108) | 0.0244168 | 1 | SURF1;SDHAF3;NDUFS2 |
| homotypic cell-cell adhesion (GO:0034109) | 0.024586084 | 1 | HBB;METAP1 |
| positive regulation of innate immune response (GO:0045089) | 0.024586084 | 1 | DHX9;RIOK3 |
| endocytosis (GO:0006897) | 0.027156365 | 1 | SNX2;BIN1;HBB;WASL;PICALM |
| cellular response to cytokine stimulus (GO:0071345) | 0.027484921 | 1 | MT2A;DHX9;LCN2;MT1X;CXCL1;IL6R;DUOX2 |
| bicarbonate transport (GO:0015701) | 0.028324708 | 1 | HBB;CFTR |
| positive regulation of receptor-mediated endocytosis (GO:0048260) | 0.028324708 | 1 | WASL;PICALM |
| response to hydrogen peroxide (GO:0042542) | 0.030935397 | 1 | CAT;HBB |
| positive regulation of interleukin-6 production (GO:0032755) | 0.030935397 | 1 | DHX9;IL6R |
| viral life cycle (GO:0019058) | 0.031403905 | 1 | VPS4A;UBP1;VPS28 |
| hexose metabolic process (GO:0019318) | 0.033637462 | 1 | FUCA2;PCK1 |
| cellular response to peptide hormone stimulus (GO:0071375) | 0.033683321 | 1 | RAB10;PCK1;PRKACB |
| RNA metabolic process (GO:0016070) | 0.034473437 | 1 | PABPN1;DHX9;POLR2B;RNASET2 |
| positive regulation of tumor necrosis factor production (GO:0032760) | 0.036428186 | 1 | DHX9;DDT |
| positive regulation of nucleobase-containing compound transport (GO:0032241) | 0.037795259 | 1 | DHX9 |
| arginine biosynthetic process (GO:0006526) | 0.037795259 | 1 | ASL |
| regulation of keratinocyte apoptotic process (GO:1902172) | 0.037795259 | 1 | GSN |
| intracellular pH elevation (GO:0051454) | 0.037795259 | 1 | CFTR |
| dosage compensation by inactivation of X chromosome (GO:0009048) | 0.037795259 | 1 | PCGF5 |
| positive regulation of aspartic-type peptidase activity (GO:1905247) | 0.037795259 | 1 | PICALM |
| viral release from host cell (GO:0019076) | 0.037795259 | 1 | VPS4A |
| N-terminal peptidyl-methionine acetylation (GO:0017196) | 0.037795259 | 1 | NAA60 |
| positive regulation of amyloid-beta clearance (GO:1900223) | 0.037795259 | 1 | PICALM |
| glycosyl compound catabolic process (GO:1901658) | 0.037795259 | 1 | FUCA2 |
| negative regulation of lymphocyte migration (GO:2000402) | 0.037795259 | 1 | WASL |
| negative regulation of cytokinesis (GO:0032466) | 0.037795259 | 1 | VPS4A |
| bundle of His cell-Purkinje myocyte adhesion involved in cell communication (GO:0086073) | 0.037795259 | 1 | JUP |
| ceramide transport (GO:0035627) | 0.037795259 | 1 | CPTP |
| cardiolipin acyl-chain remodeling (GO:0035965) | 0.037795259 | 1 | HADHB |
| positive regulation of interferon-alpha secretion (GO:1902741) | 0.037795259 | 1 | DHX9 |
| cellular nitrogen compound catabolic process (GO:0044270) | 0.037795259 | 1 | MPST |
| iron ion import (GO:0097286) | 0.037795259 | 1 | PICALM |
| negative regulation of sterol transport (GO:0032372) | 0.037795259 | 1 | APOC1 |
| negative regulation of phospholipid metabolic process (GO:1903726) | 0.037795259 | 1 | APOC1 |
| iron ion import across plasma membrane (GO:0098711) | 0.037795259 | 1 | PICALM |
| exit from host cell (GO:0035891) | 0.037795259 | 1 | VPS4A |
| insulin secretion involved in cellular response to glucose stimulus (GO:0035773) | 0.037795259 | 1 | RAB11B |
| regulation of interferon-alpha secretion (GO:1902739) | 0.037795259 | 1 | DHX9 |
| positive regulation of aspartic-type endopeptidase activity involved in amyloid precursor protein catabolic process (GO:1902961) | 0.037795259 | 1 | PICALM |
| positive regulation of RNA export from nucleus (GO:0046833) | 0.037795259 | 1 | DHX9 |
| positive regulation of chemotaxis (GO:0050921) | 0.03785596 | 1 | CXCL1;IL6R |
| establishment of protein localization to mitochondrion (GO:0072655) | 0.039304905 | 1 | GRPEL1;AIP |
| cytosolic transport (GO:0016482) | 0.040170351 | 1 | TMEM87B;SNX2;STX10 |
| regulation of exocytosis (GO:0017157) | 0.042265007 | 1 | RAB10;CFTR |
| positive regulation of actin filament polymerization (GO:0030838) | 0.042265007 | 1 | GSN;WASL |
| protein localization to basolateral plasma membrane (GO:1903361) | 0.0439552 | 1 | RAB10 |
| cardiac muscle cell-cardiac muscle cell adhesion (GO:0086042) | 0.0439552 | 1 | JUP |
| protein localization to cytoplasmic stress granule (GO:1903608) | 0.0439552 | 1 | DHX9 |
| intracellular sterol transport (GO:0032366) | 0.0439552 | 1 | VPS4A |
| regulation of entry of bacterium into host cell (GO:2000535) | 0.0439552 | 1 | FUCA2 |
| negative regulation of leukocyte migration (GO:0002686) | 0.0439552 | 1 | WASL |
| regulation of chromatin assembly or disassembly (GO:0001672) | 0.0439552 | 1 | TLK1 |
| regulation of endocytic recycling (GO:2001135) | 0.0439552 | 1 | RAB11B |
| regulation of MDA-5 signaling pathway (GO:0039533) | 0.0439552 | 1 | RIOK3 |
| oxygen transport (GO:0015671) | 0.0439552 | 1 | HBB |
| regulation of lymphocyte migration (GO:2000401) | 0.0439552 | 1 | WASL |
| glycoside catabolic process (GO:0016139) | 0.0439552 | 1 | FUCA2 |
| vesicle transport along actin filament (GO:0030050) | 0.0439552 | 1 | WASL |
| regulation of anion transport (GO:0044070) | 0.0439552 | 1 | RAB11B |
| G-quadruplex DNA unwinding (GO:0044806) | 0.0439552 | 1 | DHX9 |
| regulation of sterol transport (GO:0032371) | 0.0439552 | 1 | APOC1 |
| positive regulation of isomerase activity (GO:0010912) | 0.0439552 | 1 | DHX9 |
| formation of translation preinitiation complex (GO:0001731) | 0.0439552 | 1 | DENR |
| regulation of interleukin-18 production (GO:0032661) | 0.0439552 | 1 | DHX9 |
| negative regulation of retinoic acid receptor signaling pathway (GO:0048387) | 0.0439552 | 1 | CNOT1 |
| positive regulation of protein deubiquitination (GO:1903003) | 0.0439552 | 1 | NOP53 |
| chylomicron remnant clearance (GO:0034382) | 0.0439552 | 1 | APOC1 |
| morphogenesis of a polarized epithelium (GO:0001738) | 0.0439552 | 1 | RAB10 |
| negative regulation of nitrogen compound metabolic process (GO:0051172) | 0.0439552 | 1 | APOC1 |
| protein targeting to mitochondrion (GO:0006626) | 0.046855918 | 1 | GRPEL1;AIP |
| negative regulation of cellular component organization (GO:0051129) | 0.046855918 | 1 | WASL;NOP53 |
| positive regulation of immune response (GO:0050778) | 0.046855918 | 1 | DHX9;RIOK3 |
| positive regulation of response to stimulus (GO:0048584) | 0.046855918 | 1 | DHX9;CFTR |

**C) RHI**

| **Biological Process** | **P-value** | **Adjusted P-value** | **Genes** |
| --- | --- | --- | --- |
| positive regulation of mitochondrial calcium ion concentration (GO:0051561) | 4.69E-04 | 1 | FIS1;MCU |
| mitochondrial translational elongation (GO:0070125) | 4.72E-04 | 1 | MRPS9;MRPL1;MRPL54;TUFM |
| positive regulation of mesenchymal cell proliferation (GO:0002053) | 9.13E-04 | 1 | STAT1;TGFBR2 |
| translational elongation (GO:0006414) | 9.59E-04 | 1 | MRPS9;MRPL1;MRPL54;TUFM |
| mitochondrial translation (GO:0032543) | 0.001028594 | 1 | MRPS9;MRPL1;MRPL54;TUFM |
| regulation of mesenchymal cell proliferation (GO:0010464) | 0.001093102 | 0.929683261 | STAT1;TGFBR2 |
| response to purine-containing compound (GO:0014074) | 0.002222324 | 1 | STAT1;DUOX2 |
| viral entry into host cell (GO:0046718) | 0.002222324 | 1 | AXL;WWP1 |
| response to organophosphorus (GO:0046683) | 0.002493396 | 1 | STAT1;DUOX2 |
| release of cytochrome c from mitochondria (GO:0001836) | 0.002493396 | 1 | FIS1;IFI6 |
| endoplasmic reticulum calcium ion homeostasis (GO:0032469) | 0.002493396 | 1 | FIS1;HERPUD1 |
| mitochondrial calcium ion homeostasis (GO:0051560) | 0.002779249 | 1 | FIS1;MCU |
| dendritic cell differentiation (GO:0097028) | 0.003394807 | 1 | AXL;TGFBR2 |
| negative regulation of response to endoplasmic reticulum stress (GO:1903573) | 0.003724263 | 1 | UBXN1;HERPUD1 |
| STAT cascade (GO:0097696) | 0.003724263 | 1 | STAT1;NMI |
| outflow tract septum morphogenesis (GO:0003148) | 0.003724263 | 1 | GATA6;TGFBR2 |
| mitochondrial translational termination (GO:0070126) | 0.006055414 | 1 | MRPS9;MRPL1;MRPL54 |
| translational termination (GO:0006415) | 0.00746237 | 1 | MRPS9;MRPL1;MRPL54 |
| antigen processing and presentation of exogenous peptide antigen (GO:0002478) | 0.007677789 | 1 | DCTN6;DYNC1LI2;DCTN3 |
| antigen processing and presentation of exogenous peptide antigen via MHC class II (GO:0019886) | 0.007677789 | 1 | DCTN6;DYNC1LI2;DCTN3 |
| antigen processing and presentation of peptide antigen via MHC class II (GO:0002495) | 0.007896859 | 1 | DCTN6;DYNC1LI2;DCTN3 |
| entry into host cell (GO:0030260) | 0.00826504 | 1 | AXL;WWP1 |
| apoptotic mitochondrial changes (GO:0008637) | 0.008758118 | 1 | FIS1;IFI6 |
| viral life cycle (GO:0019058) | 0.010034976 | 1 | AXL;UBP1;WWP1 |
| response to cAMP (GO:0051591) | 0.011983166 | 1 | STAT1;DUOX2 |
| JAK-STAT cascade (GO:0007259) | 0.011983166 | 1 | STAT1;NMI |
| nucleobase-containing compound catabolic process (GO:0034655) | 0.01376208 | 1 | RNASET2;NUDT16 |
| RNA catabolic process (GO:0006401) | 0.01376208 | 1 | RNASET2;NUDT16 |
| apoptotic process (GO:0006915) | 0.015660188 | 1 | FIS1;CASP5;CDK11B;TGFBR2 |
| regulation of intrinsic apoptotic signaling pathway (GO:2001242) | 0.01629995 | 1 | FIS1;HERPUD1 |
| macrophage derived foam cell differentiation (GO:0010742) | 0.024645968 | 1 | STAT1 |
| mesenchymal cell differentiation involved in kidney development (GO:0072161) | 0.024645968 | 1 | STAT1 |
| regulation of epithelial to mesenchymal transition involved in endocardial cushion formation (GO:1905005) | 0.024645968 | 1 | TGFBR2 |
| arginine biosynthetic process (GO:0006526) | 0.024645968 | 1 | ASL |
| negative regulation of transforming growth factor beta production (GO:0071635) | 0.024645968 | 1 | GATA6 |
| protein geranylgeranylation (GO:0018344) | 0.024645968 | 1 | FNTA |
| positive regulation of deacetylase activity (GO:0090045) | 0.024645968 | 1 | FNTA |
| regulation of transforming growth factor beta2 production (GO:0032909) | 0.024645968 | 1 | GATA6 |
| negative regulation of cytoplasmic translation (GO:2000766) | 0.024645968 | 1 | CPEB4 |
| citrate metabolic process (GO:0006101) | 0.024645968 | 1 | ACO2 |
| vascular smooth muscle cell differentiation (GO:0035886) | 0.024645968 | 1 | GATA6 |
| regulation of T cell tolerance induction (GO:0002664) | 0.024645968 | 1 | TGFBR2 |
| snRNA modification (GO:0040031) | 0.024645968 | 1 | MRPL1 |
| negative regulation of endoplasmic reticulum calcium ion concentration (GO:0032471) | 0.024645968 | 1 | FIS1 |
| coronary vasculature development (GO:0060976) | 0.024645968 | 1 | GATA6 |
| positive regulation of clathrin-dependent endocytosis (GO:2000370) | 0.024645968 | 1 | SCYL2 |
| foam cell differentiation (GO:0090077) | 0.024645968 | 1 | STAT1 |
| Golgi vesicle transport (GO:0048193) | 0.026323541 | 1 | DCTN6;DYNC1LI2;DCTN3;NUDT19 |
| xenobiotic catabolic process (GO:0042178) | 0.028694887 | 1 | GSTM4 |
| positive regulation of mitotic sister chromatid separation (GO:1901970) | 0.028694887 | 1 | ANAPC5 |
| regulation by virus of viral protein levels in host cell (GO:0046719) | 0.028694887 | 1 | STAT1 |
| cellular response to interferon-alpha (GO:0035457) | 0.028694887 | 1 | AXL |
| flavin-containing compound metabolic process (GO:0042726) | 0.028694887 | 1 | SLC52A2 |
| positive regulation of metaphase/anaphase transition of cell cycle (GO:1902101) | 0.028694887 | 1 | ANAPC5 |
| regulation of pinocytosis (GO:0048548) | 0.028694887 | 1 | AXL |
| positive regulation of ER-associated ubiquitin-dependent protein catabolic process (GO:1903071) | 0.028694887 | 1 | HERPUD1 |
| riboflavin metabolic process (GO:0006771) | 0.028694887 | 1 | SLC52A2 |
| positive regulation of CD4-positive, alpha-beta T cell activation (GO:2000516) | 0.028694887 | 1 | TGFBR2 |
| negative regulation of dendritic cell apoptotic process (GO:2000669) | 0.028694887 | 1 | AXL |
| cellular response to decreased oxygen levels (GO:0036294) | 0.029054566 | 1 | GATA6;CPEB4 |
| cellular response to type I interferon (GO:0071357) | 0.029897537 | 1 | STAT1;IFI6 |
| type I interferon signaling pathway (GO:0060337) | 0.029897537 | 1 | STAT1;IFI6 |
| fatty acid catabolic process (GO:0009062) | 0.029897537 | 1 | NUDT19;ECHDC1 |
| protein lipoylation (GO:0009249) | 0.032727202 | 1 | GLRX5 |
| cellular response to interleukin-21 (GO:0098757) | 0.032727202 | 1 | STAT1 |
| galactose catabolic process (GO:0019388) | 0.032727202 | 1 | GALE |
| rRNA pseudouridine synthesis (GO:0031118) | 0.032727202 | 1 | MRPL1 |
| positive regulation of alpha-beta T cell differentiation (GO:0046638) | 0.032727202 | 1 | TGFBR2 |
| positive regulation of mitotic metaphase/anaphase transition (GO:0045842) | 0.032727202 | 1 | ANAPC5 |
| interleukin-21-mediated signaling pathway (GO:0038114) | 0.032727202 | 1 | STAT1 |
| regulation of ERAD pathway (GO:1904292) | 0.032727202 | 1 | UBXN1 |
| calcium-mediated signaling (GO:0019722) | 0.034255935 | 1 | FIS1;MCU |
| positive regulation of proteasomal ubiquitin-dependent protein catabolic process (GO:0032436) | 0.034255935 | 1 | RNF114;HERPUD1 |
| hexose catabolic process (GO:0019320) | 0.036742978 | 1 | GALE |
| production of siRNA involved in RNA interference (GO:0030422) | 0.036742978 | 1 | TSNAX |
| ornithine metabolic process (GO:0006591) | 0.036742978 | 1 | ASL |
| atrioventricular valve development (GO:0003171) | 0.036742978 | 1 | TGFBR2 |
| positive regulation of hemostasis (GO:1900048) | 0.036742978 | 1 | ENPP4 |
| positive regulation of coagulation (GO:0050820) | 0.036742978 | 1 | ENPP4 |
| peroxisome fission (GO:0016559) | 0.036742978 | 1 | FIS1 |
| mitochondrial fragmentation involved in apoptotic process (GO:0043653) | 0.036742978 | 1 | FIS1 |
| columnar/cuboidal epithelial cell differentiation (GO:0002065) | 0.036742978 | 1 | GATA6 |
| mitochondrial calcium uptake (GO:0036444) | 0.036742978 | 1 | MCU |
| regulation of ER-associated ubiquitin-dependent protein catabolic process (GO:1903069) | 0.036742978 | 1 | HERPUD1 |
| cellular response to oxygen levels (GO:0071453) | 0.036742978 | 1 | CPEB4 |
| ionotropic glutamate receptor signaling pathway (GO:0035235) | 0.036742978 | 1 | CPEB4 |
| mitochondrion morphogenesis (GO:0070584) | 0.036742978 | 1 | FIS1 |
| receptor recycling (GO:0001881) | 0.036742978 | 1 | PLEKHJ1 |
| ER to Golgi vesicle-mediated transport (GO:0006888) | 0.039014654 | 1 | DCTN6;DYNC1LI2;DCTN3 |
| transforming growth factor beta receptor signaling pathway (GO:0007179) | 0.039789801 | 1 | FNTA;TGFBR2 |
| hemopoiesis (GO:0030097) | 0.039789801 | 1 | GLRX5;TGFBR2 |
| protein-cofactor linkage (GO:0018065) | 0.040742283 | 1 | GLRX5 |
| cell differentiation involved in metanephros development (GO:0072202) | 0.040742283 | 1 | STAT1 |
| positive regulation of natural killer cell activation (GO:0032816) | 0.040742283 | 1 | AXL |
| regulation of dendritic cell apoptotic process (GO:2000668) | 0.040742283 | 1 | AXL |
| galactose metabolic process (GO:0006012) | 0.040742283 | 1 | GALE |
| regulation of cytoplasmic translation (GO:2000765) | 0.040742283 | 1 | CPEB4 |
| smooth muscle cell differentiation (GO:0051145) | 0.040742283 | 1 | GATA6 |
| regulation of mitochondrial depolarization (GO:0051900) | 0.040742283 | 1 | IFI6 |
| protein prenylation (GO:0018342) | 0.040742283 | 1 | FNTA |
| myeloid dendritic cell differentiation (GO:0043011) | 0.040742283 | 1 | TGFBR2 |
| thyroid hormone generation (GO:0006590) | 0.040742283 | 1 | DUOX2 |
| epithelial cell morphogenesis (GO:0003382) | 0.040742283 | 1 | POF1B |
| RNA transport (GO:0050658) | 0.04365429 | 1 | RNPS1;TGFBR2 |
| cellular response to interferon-beta (GO:0035458) | 0.044725183 | 1 | STAT1 |
| negative regulation of morphogenesis of an epithelium (GO:1905331) | 0.044725183 | 1 | STAT1 |
| interleukin-27-mediated signaling pathway (GO:0070106) | 0.044725183 | 1 | STAT1 |
| cell part morphogenesis (GO:0032990) | 0.044725183 | 1 | FIS1 |
| embryonic hemopoiesis (GO:0035162) | 0.044725183 | 1 | TGFBR2 |
| positive regulation of extracellular matrix organization (GO:1903055) | 0.044725183 | 1 | COLGALT1 |
| metanephric mesenchyme development (GO:0072075) | 0.044725183 | 1 | STAT1 |
| interleukin-35-mediated signaling pathway (GO:0070757) | 0.044725183 | 1 | STAT1 |
| positive regulation of protein deacetylation (GO:0090312) | 0.044725183 | 1 | FNTA |
| urea cycle (GO:0000050) | 0.044725183 | 1 | ASL |
| response to sterol (GO:0036314) | 0.044725183 | 1 | TGFBR2 |
| positive regulation of cell cycle (GO:0045787) | 0.045637037 | 1 | USP2;NUDT16 |
| PERK-mediated unfolded protein response (GO:0036499) | 0.048691746 | 1 | HERPUD1 |
| negative regulation of ERAD pathway (GO:1904293) | 0.048691746 | 1 | UBXN1 |
| regulation of sister chromatid cohesion (GO:0007063) | 0.048691746 | 1 | CTCF |
| negative regulation of leukocyte apoptotic process (GO:2000107) | 0.048691746 | 1 | AXL |
| regulation of rRNA processing (GO:2000232) | 0.048691746 | 1 | NUDT16 |

**D) Nancy**

| **Biological Process** | **P-value** | **Adjusted P-value** | **Genes** |
| --- | --- | --- | --- |
| regulation of mitotic cell cycle (GO:0007346) | 2.20E-04 | 1 | DUSP3;ANGEL2;USP2;CDK1;PIM2;OXSR1;CDK11B;THAP1 |
| protein acetylation (GO:0006473) | 2.87E-04 | 0.733157139 | ESCO1;GTF2B;EHHADH;PCK1 |
| negative regulation of ubiquitin-protein ligase activity involved in mitotic cell cycle (GO:0051436) | 6.47E-04 | 1 | PSMD7;CDK1;UBE2D1;ANAPC5;PSMF1 |
| regulation of ubiquitin-protein ligase activity involved in mitotic cell cycle (GO:0051439) | 6.90E-04 | 0.880181796 | PSMD7;CDK1;UBE2D1;ANAPC5;PSMF1 |
| positive regulation of ubiquitin-protein ligase activity involved in regulation of mitotic cell cycle transition (GO:0051437) | 8.83E-04 | 0.901066525 | PSMD7;CDK1;UBE2D1;ANAPC5;PSMF1 |
| negative regulation of ubiquitin protein ligase activity (GO:1904667) | 9.37E-04 | 0.796765559 | PSMD7;CDK1;UBE2D1;ANAPC5;PSMF1 |
| anaphase-promoting complex-dependent catabolic process (GO:0031145) | 0.001052008 | 0.766913782 | PSMD7;CDK1;UBE2D1;ANAPC5;PSMF1 |
| positive regulation of ubiquitin protein ligase activity (GO:1904668) | 0.00124406 | 0.793554918 | PSMD7;CDK1;UBE2D1;ANAPC5;PSMF1 |
| regulation of cell cycle (GO:0051726) | 0.001262447 | 0.71580734 | RNF167;WEE1;ZNF703;CDK1;PIM2;OXSR1;CDK11B;THAP1 |
| blood vessel endothelial cell proliferation involved in sprouting angiogenesis (GO:0002043) | 0.001382548 | 0.705514009 | ITGB1BP1;NRARP |
| fatty acid oxidation (GO:0019395) | 0.001421481 | 0.659437897 | EHHADH;ECHDC1;SLC27A2;ACAT1 |
| fatty acid beta-oxidation (GO:0006635) | 0.001421481 | 0.604484739 | EHHADH;ECHDC1;SLC27A2;ACAT1 |
| positive regulation of protein ubiquitination involved in ubiquitin-dependent protein catabolic process (GO:2000060) | 0.001620088 | 0.635946661 | PSMD7;CDK1;UBE2D1;ANAPC5;PSMF1 |
| adherens junction assembly (GO:0034333) | 0.001923155 | 0.700990161 | RAMP2;ZNF703 |
| positive regulation of mitochondrial calcium ion concentration (GO:0051561) | 0.002547777 | 0.866753771 | FIS1;MCU |
| regulation of embryonic development (GO:0045995) | 0.002766843 | 0.88244988 | POGLUT1;RBM19;CDK1 |
| positive regulation of type I interferon production (GO:0032481) | 0.00314481 | 0.94399795 | POLR3GL;POLR1C;FLOT1;PQBP1 |
| fatty acid catabolic process (GO:0009062) | 0.003731445 | 1 | EHHADH;ECHDC1;SLC27A2;ACAT1 |
| DNA-templated transcription, elongation (GO:0006354) | 0.004149045 | 1 | GTF2B;POLR1C;POLR2G;NELFCD;SSRP1 |
| internal protein amino acid acetylation (GO:0006475) | 0.004909119 | 1 | EHHADH;PCK1 |
| regulation of ubiquitin protein ligase activity (GO:1904666) | 0.005143232 | 1 | CDK1;UBE2D1;ANAPC5 |
| negative regulation of substrate adhesion-dependent cell spreading (GO:1900025) | 0.00585331 | 1 | AP1AR;ITGB1BP1 |
| cellular response to exogenous dsRNA (GO:0071360) | 0.006873392 | 1 | FLOT1;PQBP1 |
| regulation of viral transcription (GO:0046782) | 0.006917351 | 1 | GTF2B;POLR2G;NELFCD |
| positive regulation of viral transcription (GO:0050434) | 0.007410629 | 1 | GTF2B;POLR2G;NELFCD |
| neutrophil degranulation (GO:0043312) | 0.007529424 | 1 | CTSA;PSMD7;ACTR1B;RNASET2;FUCA2;LCN2;ENPP4;S100P;RHOF;CCT8;SLC27A2 |
| proteasome-mediated ubiquitin-dependent protein catabolic process (GO:0043161) | 0.007905834 | 1 | RNF11;PSMD7;CDK1;UBE2D1;YOD1;ANAPC5;PSMF1;RNF4 |
| neutrophil activation involved in immune response (GO:0002283) | 0.007988109 | 1 | CTSA;PSMD7;ACTR1B;RNASET2;FUCA2;LCN2;ENPP4;S100P;RHOF;CCT8;SLC27A2 |
| neutrophil mediated immunity (GO:0002446) | 0.008468287 | 1 | CTSA;PSMD7;ACTR1B;RNASET2;FUCA2;LCN2;ENPP4;S100P;RHOF;CCT8;SLC27A2 |
| proteasomal protein catabolic process (GO:0010498) | 0.008767529 | 1 | RNF11;PSMD7;CDK1;UBE2D1;ANAPC5;PSMF1;RNF4 |
| sprouting angiogenesis (GO:0002040) | 0.009013032 | 1 | RAMP2;NRARP;ITGB1BP1 |
| positive regulation of viral process (GO:0048524) | 0.009013032 | 1 | GTF2B;POLR2G;NELFCD |
| regulation of mitotic cell cycle phase transition (GO:1901990) | 0.009454292 | 1 | PSMD7;CEP70;CDK1;UBE2D1;ANAPC5;PSMF1 |
| DNA-templated transcription, initiation (GO:0006352) | 0.009938443 | 1 | GTF2B;MED31;POLR1C;CDK1;POLR2G;NR3C2 |
| cellular response to dsRNA (GO:0071359) | 0.010373554 | 1 | FLOT1;PQBP1 |
| mitochondrial translational elongation (GO:0070125) | 0.010400666 | 1 | MRPS9;MRPL1;MRPL54;TUFM |
| 3'-UTR-mediated mRNA stabilization (GO:0070935) | 0.011681883 | 1 | ANGEL2;ELAVL1 |
| regulation of receptor recycling (GO:0001919) | 0.011681883 | 1 | AP1AR;VAMP3 |
| protein deubiquitination (GO:0016579) | 0.013257767 | 1 | PSMD7;TNIP2;USP2;CDK1;UBE2D1;YOD1;PSMF1 |
| transcription elongation from RNA polymerase II promoter (GO:0006368) | 0.014012754 | 1 | GTF2B;POLR2G;NELFCD;SSRP1 |
| positive regulation of mitotic cell cycle (GO:0045931) | 0.014211962 | 1 | DUSP3;EDN3;USP2 |
| protein modification by small protein removal (GO:0070646) | 0.014323582 | 1 | PSMD7;TNIP2;USP2;CDK1;UBE2D1;YOD1;PSMF1 |
| mitochondrial calcium ion homeostasis (GO:0051560) | 0.014502135 | 1 | FIS1;MCU |
| mitochondrial respiratory chain complex assembly (GO:0033108) | 0.01502522 | 1 | SDHAF3;ECSIT;TTC19;PET117 |
| inositol phosphate-mediated signaling (GO:0048016) | 0.016011193 | 1 | EDN3;DHX8 |
| steroid metabolic process (GO:0008202) | 0.01608275 | 1 | SULT1A1;CYP2B6;DHRS11;MSMO1 |
| protein stabilization (GO:0050821) | 0.016752939 | 1 | TNIP2;USP2;FLOT1;PIM2;CCT8 |
| ubiquitin-dependent protein catabolic process (GO:0006511) | 0.019000844 | 1 | RNF11;PSMD7;CDK1;UBE2D1;ANAPC5;PSMF1;NTAN1;RNF4 |
| positive regulation of protein targeting to membrane (GO:0090314) | 0.019220169 | 1 | FIS1;ITGB1BP1 |
| translational elongation (GO:0006414) | 0.019531351 | 1 | MRPS9;MRPL1;MRPL54;TUFM |
| protein localization to membrane (GO:0072657) | 0.020427695 | 1 | RAB32;RAMP2;FLOT1;ITGB1BP1;PKP3 |
| transcription initiation from RNA polymerase II promoter (GO:0006367) | 0.020427695 | 1 | GTF2B;MED31;CDK1;POLR2G;NR3C2 |
| mitochondrial translation (GO:0032543) | 0.020774585 | 1 | MRPS9;MRPL1;MRPL54;TUFM |
| regulation of cytokine production (GO:0001817) | 0.02141403 | 1 | TWSG1;BTN3A1;BTN2A1;FLOT1 |
| T cell receptor signaling pathway (GO:0050852) | 0.021931319 | 1 | PSMD7;BTN3A1;BTN2A1;UBE2D1;PSMF1 |
| second-messenger-mediated signaling (GO:0019932) | 0.022666445 | 1 | EDN3;DHX8;MCU |
| negative regulation of endothelial cell apoptotic process (GO:2000352) | 0.022674533 | 1 | RAMP2;TNIP2 |
| transcription, DNA-templated (GO:0006351) | 0.023834234 | 1 | POLR3GL;GTF2B;POLR1C;GTF3A;POLR2G;NELFCD;SSRP1;THAP1 |
| cellular response to vascular endothelial growth factor stimulus (GO:0035924) | 0.024490401 | 1 | RAMP2;ITGB1BP1 |
| positive regulation of developmental process (GO:0051094) | 0.024791527 | 1 | FIS1;EDN3;ZNF703;RBM19 |
| glycolipid metabolic process (GO:0006664) | 0.025632723 | 1 | CTSA;POGLUT1;CPTP |
| regulation of protein targeting to membrane (GO:0090313) | 0.028293013 | 1 | FIS1;ITGB1BP1 |
| transcription from RNA polymerase III promoter (GO:0006383) | 0.028293013 | 1 | POLR3GL;GTF3A |
| RNA splicing, via transesterification reactions with bulged adenosine as nucleophile (GO:0000377) | 0.028471594 | 1 | DHX8;SRSF2;POLR2G;TXNL4A;ELAVL1;PQBP1 |
| RNA phosphodiester bond hydrolysis (GO:0090501) | 0.030277218 | 1 | MRPL1;NOB1 |
| regulation of receptor internalization (GO:0002090) | 0.030277218 | 1 | FLOT1;SCYL2 |
| protein K11-linked ubiquitination (GO:0070979) | 0.030277218 | 1 | ANAPC5;RNF4 |
| regulation of Notch signaling pathway (GO:0008593) | 0.031016916 | 1 | POGLUT1;NRARP;ITGB1BP1 |
| negative regulation of epithelial cell apoptotic process (GO:1904036) | 0.032315028 | 1 | RAMP2;TNIP2 |
| negative regulation of cell morphogenesis involved in differentiation (GO:0010771) | 0.03440522 | 1 | AP1AR;ITGB1BP1 |
| canonical Wnt signaling pathway (GO:0060070) | 0.034509941 | 1 | HOXB9;FZD5;SCYL2 |
| antigen processing and presentation of exogenous peptide antigen via MHC class I, TAP-dependent (GO:0002479) | 0.036947033 | 1 | PSMD7;PSMF1;VAMP3 |
| regulation of macromolecule metabolic process (GO:0060255) | 0.037664023 | 1 | EDN3;CDK1;FLOT1;CDK11B |
| antigen processing and presentation of exogenous peptide antigen via MHC class I (GO:0042590) | 0.040763777 | 1 | PSMD7;PSMF1;VAMP3 |
| regulation of transcription from RNA polymerase II promoter in response to hypoxia (GO:0061418) | 0.040763777 | 1 | PSMD7;UBE2D1;PSMF1 |
| positive regulation of Notch signaling pathway (GO:0045747) | 0.040978145 | 1 | POGLUT1;ITGB1BP1 |
| response to exogenous dsRNA (GO:0043330) | 0.040978145 | 1 | FLOT1;PQBP1 |
| mRNA stabilization (GO:0048255) | 0.040978145 | 1 | ANGEL2;ELAVL1 |
| steroid biosynthetic process (GO:0006694) | 0.042078641 | 1 | DHRS11;MSMO1;SLC27A2 |
| mRNA splicing, via spliceosome (GO:0000398) | 0.043123662 | 1 | DHX8;SRSF2;POLR2G;TXNL4A;ELAVL1;PQBP1 |
| regulation of interleukin-1 beta secretion (GO:0050706) | 0.04326601 | 1 | FZD5;CPTP |
| negative regulation of cell-substrate adhesion (GO:0010812) | 0.04560042 | 1 | AP1AR;ITGB1BP1 |
| regulation of endothelial cell apoptotic process (GO:2000351) | 0.04560042 | 1 | RAMP2;TNIP2 |
| actin filament bundle organization (GO:0061572) | 0.04560042 | 1 | MYO1B;RHOF |
| regulation of receptor-mediated endocytosis (GO:0048259) | 0.047980258 | 1 | APOC1;FLOT1 |
| epithelial cell migration (GO:0010631) | 0.047980258 | 1 | FERMT1;S100P |
| positive regulation of signaling (GO:0023056) | 0.047980258 | 1 | EDN3;VAMP3 |
| secondary alcohol biosynthetic process (GO:1902653) | 0.047980258 | 1 | MSMO1;ACAT1 |
| cellular response to molecule of bacterial origin (GO:0071219) | 0.048968135 | 1 | FZD5;TNIP2;AXL |
| modification-dependent protein catabolic process (GO:0019941) | 0.049418585 | 1 | RNF11;UBE2D1;PSMF1;NTAN1 |
