# Supplementary material for: Ultra-high Magnification Endocytoscopy and Molecular Markers for Defining Endoscopic and Histologic Remission in Ulcerative Colitis—An Exploratory Study to Define Deep Remission
Source: Inflamm Bowel Dis. 2021 May 21;27(11):1719–30. doi: 10.1093/ibd/izab059 (PMC8528147; doi:10.1093/ibd/izab059)
Supplement: izab059_suppl_Supplementary_Table_7 [file izab059_suppl_supplementary_table_7.docx]

**Supplementary Table 7:** KEGG Pathway and GO Biological Process enrichment analysis of genes commonly upregulated in healed mucosa defined by ECSS and RHI (A and B) and ECSS and Nancy (C and D) scores. The genes involved in each pathway are listed.

**A:** KEGG Pathway analysis of the 25 common upregulated genes in healed vs non-healed mucosa defined by ECSS and RHI scores.

| **Pathway** | **P-value** | **Adjusted P-value** | **Genes** |
| --- | --- | --- | --- |
| Arginine biosynthesis | 0.025937 | 1 | ASL |
| Terpenoid backbone biosynthesis | 0.027156 | 1 | FNTA |
| Glyoxylate and dicarboxylate metabolism | 0.036854 | 1 | ACO2 |
| Citrate cycle (TCA cycle) | 0.036854 | 1 | ACO2 |
| Alanine, aspartate and glutamate metabolism | 0.042868 | 1 | ASL |
| Arachidonic acid metabolism | 0.075887 | 1 | CYP2B6 |
| Retinol metabolism | 0.080514 | 1 | CYP2B6 |
| Metabolism of xenobiotics by cytochrome P450 | 0.088558 | 1 | CYP2B6 |
| Progesterone-mediated oocyte maturation | 0.116736 | 1 | CPEB4 |
| Drug metabolism | 0.126674 | 1 | CYP2B6 |
| Oocyte meiosis | 0.145154 | 1 | CPEB4 |
| Purine metabolism | 0.149448 | 1 | ENPP4 |
| Cellular senescence | 0.182026 | 1 | MCU |
| NOD-like receptor signaling pathway | 0.200389 | 1 | MCU |
| Calcium signaling pathway | 0.210419 | 1 | MCU |

**B:** Go Biological Process Pathway analysis of the 25 common upregulated genes in healed vs non-healed mucosa defined by ECSS and RHI scores.

| **Biological process** | **P-value** | **Adjusted P-value** | **Genes** |
| --- | --- | --- | --- |
| protein geranylgeranylation (GO:0018344) | 0.007477 | 1 | FNTA |
| positive regulation of deacetylase activity (GO:0090045) | 0.007477 | 1 | FNTA |
| negative regulation of cytoplasmic translation (GO:2000766) | 0.007477 | 1 | CPEB4 |
| positive regulation of clathrin-dependent endocytosis (GO:2000370) | 0.007477 | 1 | SCYL2 |
| citrate metabolic process (GO:0006101) | 0.007477 | 1 | ACO2 |
| arginine biosynthetic process (GO:0006526) | 0.007477 | 1 | ASL |
| protein lipoylation (GO:0009249) | 0.009958 | 1 | GLRX5 |
| positive regulation of mitochondrial calcium ion concentration (GO:0051561) | 0.009958 | 1 | MCU |
| production of siRNA involved in RNA interference (GO:0030422) | 0.011196 | 1 | TSNAX |
| ionotropic glutamate receptor signaling pathway (GO:0035235) | 0.011196 | 1 | CPEB4 |
| positive regulation of hemostasis (GO:1900048) | 0.011196 | 1 | ENPP4 |
| mitochondrial calcium uptake (GO:0036444) | 0.011196 | 1 | MCU |
| cellular response to oxygen levels (GO:0071453) | 0.011196 | 1 | CPEB4 |
| positive regulation of coagulation (GO:0050820) | 0.011196 | 1 | ENPP4 |
| ornithine metabolic process (GO:0006591) | 0.011196 | 1 | ASL |
| regulation of cytoplasmic translation (GO:2000765) | 0.012433 | 1 | CPEB4 |
| protein-cofactor linkage (GO:0018065) | 0.012433 | 1 | GLRX5 |
| protein prenylation (GO:0018342) | 0.012433 | 1 | FNTA |
| positive regulation of protein deacetylation (GO:0090312) | 0.013668 | 1 | FNTA |
| urea cycle (GO:0000050) | 0.013668 | 1 | ASL |
| tricarboxylic acid metabolic process (GO:0072350) | 0.016133 | 1 | ACO2 |
| RNA interference (GO:0016246) | 0.016133 | 1 | TSNAX |
| regulation of clathrin-dependent endocytosis (GO:2000369) | 0.018593 | 1 | SCYL2 |
| mitochondrial calcium ion homeostasis (GO:0051560) | 0.023495 | 1 | MCU |
| production of small RNA involved in gene silencing by RNA (GO:0070918) | 0.023495 | 1 | TSNAX |
| positive regulation of blood coagulation (GO:0030194) | 0.023495 | 1 | ENPP4 |
| entrainment of circadian clock by photoperiod (GO:0043153) | 0.024717 | 1 | USP2 |
| photoperiodism (GO:0009648) | 0.025937 | 1 | USP2 |
| mitochondrial calcium ion transmembrane transport (GO:0006851) | 0.025937 | 1 | MCU |
| epoxygenase P450 pathway (GO:0019373) | 0.025937 | 1 | CYP2B6 |
| positive regulation of receptor internalization (GO:0002092) | 0.025937 | 1 | SCYL2 |
| cytosolic calcium ion transport (GO:0060401) | 0.027156 | 1 | MCU |
| exogenous drug catabolic process (GO:0042738) | 0.028373 | 1 | CYP2B6 |
| drug catabolic process (GO:0042737) | 0.029589 | 1 | CYP2B6 |
| regulation of rhodopsin mediated signaling pathway (GO:0022400) | 0.032017 | 1 | FNTA |
| positive regulation of wound healing (GO:0090303) | 0.032017 | 1 | ENPP4 |
| regulation of receptor internalization (GO:0002090) | 0.034438 | 1 | SCYL2 |
| calcium ion import (GO:0070509) | 0.034438 | 1 | MCU |
| regulation of blood coagulation (GO:0030193) | 0.036854 | 1 | ENPP4 |
| positive regulation of insulin secretion (GO:0032024) | 0.03806 | 1 | MCU |
| mitochondrial transmembrane transport (GO:1990542) | 0.039264 | 1 | MCU |
| cellular response to amino acid stimulus (GO:0071230) | 0.039264 | 1 | CPEB4 |
| cellular response to glucose starvation (GO:0042149) | 0.040467 | 1 | CPEB4 |
| response to amino acid (GO:0043200) | 0.041668 | 1 | CPEB4 |
| glutamate receptor signaling pathway (GO:0007215) | 0.044067 | 1 | CPEB4 |
| positive regulation of peptide hormone secretion (GO:0090277) | 0.047654 | 1 | MCU |

**C:** KEGG Pathway analysis of the 79 common upregulated genes in healed vs non-healed mucosa defined by ECSS and Nancy scores.

| **Pathways** | **P-value** | **Adjusted P-value** | **Genes** |
| --- | --- | --- | --- |
| Terpenoid backbone biosynthesis | 0.003381 | 1 | FNTA;ACAT1 |
| Citrate cycle (TCA cycle) | 0.006238 | 0.960625185 | ACO2;PCK1 |
| Glyoxylate and dicarboxylate metabolism | 0.006238 | 0.64041679 | ACO2;ACAT1 |
| Pyruvate metabolism | 0.010385 | 0.799650795 | PCK1;ACAT1 |
| Spliceosome | 0.016063 | 0.989479356 | DHX8;TXNL4A;PQBP1 |
| N-Glycan biosynthesis | 0.016695 | 0.857034519 | DPM2;ALG3 |
| Synthesis and degradation of ketone bodies | 0.038814 | 1 | ACAT1 |

**D)** Go Biological Process Pathway analysis of the 79 common upregulated genes in healed vs non-healed mucosa defined by ECSS and Nancy scores.

| **Biological Process** | **P-value** | **Adjusted P-value** | **Genes** |
| --- | --- | --- | --- |
| cellular response to exogenous dsRNA (GO:0071360) | 0.001168 | 1 | FLOT1;PQBP1 |
| cellular response to dsRNA (GO:0071359) | 0.001783 | 1 | FLOT1;PQBP1 |
| positive regulation of type I interferon production (GO:0032481) | 0.001897 | 1 | POLR3GL;FLOT1;PQBP1 |
| canonical Wnt signaling pathway (GO:0060070) | 0.003024 | 1 | HOXB9;FZD5;SCYL2 |
| protein stabilization (GO:0050821) | 0.003098 | 1 | TNIP2;USP2;FLOT1;CCT8 |
| transcription from RNA polymerase III promoter (GO:0006383) | 0.005072 | 1 | POLR3GL;GTF3A |
| regulation of receptor internalization (GO:0002090) | 0.005448 | 1 | FLOT1;SCYL2 |
| regulation of embryonic development (GO:0045995) | 0.005837 | 1 | POGLUT1;RBM19 |
| response to exogenous dsRNA (GO:0043330) | 0.007514 | 1 | FLOT1;PQBP1 |
| regulation of interleukin-1 beta secretion (GO:0050706) | 0.007963 | 1 | FZD5;CPTP |
| regulation of receptor-mediated endocytosis (GO:0048259) | 0.008897 | 1 | APOC1;FLOT1 |
| positive regulation of cytokine production (GO:0001819) | 0.011258 | 1 | FZD5;POLR3GL;FLOT1;PQBP1 |
| endosome to lysosome transport (GO:0008333) | 0.015452 | 1 | BIN1;SCYL2 |
| negative regulation of cellular catabolic process (GO:0031330) | 0.020672 | 1 | CPTP;APOC1 |
| phospholipid transport (GO:0015914) | 0.022077 | 1 | CPTP;APOC1 |
| negative regulation of phospholipid metabolic process (GO:1903726) | 0.02347 | 1 | APOC1 |
| exit from host cell (GO:0035891) | 0.02347 | 1 | PPID |
| negative regulation of mRNA catabolic process (GO:1902373) | 0.02347 | 1 | PKP3 |
| arginine biosynthetic process (GO:0006526) | 0.02347 | 1 | ASL |
| protein geranylgeranylation (GO:0018344) | 0.02347 | 1 | FNTA |
| positive regulation of deacetylase activity (GO:0090045) | 0.02347 | 1 | FNTA |
| membrane raft assembly (GO:0001765) | 0.02347 | 1 | FLOT1 |
| positive regulation of toll-like receptor 3 signaling pathway (GO:0034141) | 0.02347 | 1 | FLOT1 |
| negative regulation of cytoplasmic translation (GO:2000766) | 0.02347 | 1 | CPEB4 |
| citrate metabolic process (GO:0006101) | 0.02347 | 1 | ACO2 |
| viral release from host cell (GO:0019076) | 0.02347 | 1 | PPID |
| adenylate cyclase-inhibiting G-protein coupled acetylcholine receptor signaling pathway (GO:0007197) | 0.02347 | 1 | DHX8 |
| ceramide transport (GO:0035627) | 0.02347 | 1 | CPTP |
| muscle tissue development (GO:0060537) | 0.02347 | 1 | POGLUT1 |
| positive regulation of clathrin-dependent endocytosis (GO:2000370) | 0.02347 | 1 | SCYL2 |
| response to UV-A (GO:0070141) | 0.02347 | 1 | PPID |
| acetyl-CoA biosynthetic process (GO:0006085) | 0.02347 | 1 | ACAT1 |
| negative regulation of sterol transport (GO:0032372) | 0.02347 | 1 | APOC1 |
| second-messenger-mediated signaling (GO:0019932) | 0.025002 | 1 | DHX8;MCU |
| protein localization to membrane (GO:0072657) | 0.025526 | 1 | RAB32;FLOT1;PKP3 |
| carbohydrate homeostasis (GO:0033500) | 0.025756 | 1 | PCK1;MCU |
| glycolipid metabolic process (GO:0006664) | 0.027293 | 1 | POGLUT1;CPTP |
| regulation of sterol transport (GO:0032371) | 0.027328 | 1 | APOC1 |
| cellular lipid biosynthetic process (GO:0097384) | 0.027328 | 1 | ACAT1 |
| purine nucleoside bisphosphate biosynthetic process (GO:0034033) | 0.027328 | 1 | ACAT1 |
| positive regulation of inositol phosphate biosynthetic process (GO:0060732) | 0.027328 | 1 | DHX8 |
| coenzyme A biosynthetic process (GO:0015937) | 0.027328 | 1 | ACAT1 |
| ribonucleoside bisphosphate biosynthetic process (GO:0034030) | 0.027328 | 1 | ACAT1 |
| chylomicron remnant clearance (GO:0034382) | 0.027328 | 1 | APOC1 |
| regulation of toll-like receptor 3 signaling pathway (GO:0034139) | 0.027328 | 1 | FLOT1 |
| cellular response to histamine (GO:0071420) | 0.027328 | 1 | DHX8 |
| negative regulation of nitrogen compound metabolic process (GO:0051172) | 0.027328 | 1 | APOC1 |
| regulation of mitotic cell cycle (GO:0007346) | 0.027624 | 1 | ANGEL2;USP2;OXSR1 |
| regulation of synaptic transmission, dopaminergic (GO:0032225) | 0.031172 | 1 | FLOT1 |
| positive regulation of mitochondrial calcium ion concentration (GO:0051561) | 0.031172 | 1 | MCU |
| ketone body biosynthetic process (GO:0046951) | 0.031172 | 1 | ACAT1 |
| protein lipoylation (GO:0009249) | 0.031172 | 1 | GLRX5 |
| positive regulation of protein localization to Cajal body (GO:1904871) | 0.031172 | 1 | CCT8 |
| negative regulation of lipase activity (GO:0060192) | 0.031172 | 1 | APOC1 |
| short-chain fatty acid catabolic process (GO:0019626) | 0.031172 | 1 | PCK1 |
| desmosome organization (GO:0002934) | 0.031172 | 1 | PKP3 |
| protein K6-linked ubiquitination (GO:0085020) | 0.031172 | 1 | RNF4 |
| succinate metabolic process (GO:0006105) | 0.031172 | 1 | SDHAF3 |
| regulation of aspartic-type endopeptidase activity involved in amyloid precursor protein catabolic process (GO:1902959) | 0.031172 | 1 | BIN1 |
| regulation of protein localization to Cajal body (GO:1904869) | 0.031172 | 1 | CCT8 |
| stress-activated protein kinase signaling cascade (GO:0031098) | 0.031292 | 1 | TNIP2;OXSR1 |
| lysosomal transport (GO:0007041) | 0.032952 | 1 | BIN1;SCYL2 |
| glucose homeostasis (GO:0042593) | 0.034647 | 1 | PCK1;MCU |
| positive regulation of establishment of protein localization to telomere (GO:1904851) | 0.035 | 1 | CCT8 |
| production of siRNA involved in RNA interference (GO:0030422) | 0.035 | 1 | TSNAX |
| negative regulation of protein sumoylation (GO:0033234) | 0.035 | 1 | RNF4 |
| ornithine metabolic process (GO:0006591) | 0.035 | 1 | ASL |
| endosome to melanosome transport (GO:0035646) | 0.035 | 1 | RAB32 |
| positive regulation of hemostasis (GO:1900048) | 0.035 | 1 | ENPP4 |
| endosome to pigment granule transport (GO:0043485) | 0.035 | 1 | RAB32 |
| negative regulation of amyloid-beta formation (GO:1902430) | 0.035 | 1 | BIN1 |
| positive regulation of coagulation (GO:0050820) | 0.035 | 1 | ENPP4 |
| toll-like receptor 3 signaling pathway (GO:0034138) | 0.035 | 1 | TNIP2 |
| regulation of cell-cell adhesion mediated by cadherin (GO:2000047) | 0.035 | 1 | FLOT1 |
| mitochondrial calcium uptake (GO:0036444) | 0.035 | 1 | MCU |
| cellular response to oxygen levels (GO:0071453) | 0.035 | 1 | CPEB4 |
| positive regulation of astrocyte differentiation (GO:0048711) | 0.035 | 1 | BIN1 |
| ionotropic glutamate receptor signaling pathway (GO:0035235) | 0.035 | 1 | CPEB4 |
| positive regulation of cholesterol esterification (GO:0010873) | 0.035 | 1 | APOC1 |
| regulation of astrocyte differentiation (GO:0048710) | 0.038814 | 1 | BIN1 |
| protein-cofactor linkage (GO:0018065) | 0.038814 | 1 | GLRX5 |
| membrane raft organization (GO:0031579) | 0.038814 | 1 | FLOT1 |
| negative regulation of cholesterol transport (GO:0032375) | 0.038814 | 1 | APOC1 |
| dorsal/ventral axis specification (GO:0009950) | 0.038814 | 1 | FZD5 |
| very-low-density lipoprotein particle assembly (GO:0034379) | 0.038814 | 1 | APOC1 |
| positive regulation of steroid metabolic process (GO:0045940) | 0.038814 | 1 | APOC1 |
| response to histamine (GO:0034776) | 0.038814 | 1 | DHX8 |
| mitotic nuclear envelope reassembly (GO:0007084) | 0.038814 | 1 | ANKLE2 |
| negative regulation of interleukin-1 beta secretion (GO:0050713) | 0.038814 | 1 | CPTP |
| coenzyme A metabolic process (GO:0015936) | 0.038814 | 1 | ACAT1 |
| regulation of establishment of protein localization to telomere (GO:0070203) | 0.038814 | 1 | CCT8 |
| ketone body metabolic process (GO:1902224) | 0.038814 | 1 | ACAT1 |
| regulation of cytoplasmic translation (GO:2000765) | 0.038814 | 1 | CPEB4 |
| regulation of NLRP3 inflammasome complex assembly (GO:1900225) | 0.038814 | 1 | CPTP |
| positive regulation of glial cell differentiation (GO:0045687) | 0.038814 | 1 | BIN1 |
| protein prenylation (GO:0018342) | 0.038814 | 1 | FNTA |
| positive regulation of protein secretion (GO:0050714) | 0.041752 | 1 | PPID;MCU |
| protein kinase C signaling (GO:0070528) | 0.042612 | 1 | FLOT1 |
| regulation of cholesterol esterification (GO:0010872) | 0.042612 | 1 | APOC1 |
| protein O-linked mannosylation (GO:0035269) | 0.042612 | 1 | DPM2 |
| positive regulation of heterotypic cell-cell adhesion (GO:0034116) | 0.042612 | 1 | FLOT1 |
| negative regulation of amyloid precursor protein catabolic process (GO:1902992) | 0.042612 | 1 | BIN1 |
| positive regulation of protein localization to chromosome, telomeric region (GO:1904816) | 0.042612 | 1 | CCT8 |
| T cell proliferation (GO:0042098) | 0.042612 | 1 | BTN3A1 |
| negative regulation of fatty acid biosynthetic process (GO:0045717) | 0.042612 | 1 | APOC1 |
| positive regulation of tumor necrosis factor secretion (GO:1904469) | 0.042612 | 1 | FZD5 |
| positive regulation of protein deacetylation (GO:0090312) | 0.042612 | 1 | FNTA |
| urea cycle (GO:0000050) | 0.042612 | 1 | ASL |
| internal protein amino acid acetylation (GO:0006475) | 0.042612 | 1 | PCK1 |
| positive regulation of myoblast fusion (GO:1901741) | 0.042612 | 1 | FLOT1 |
| cellular response to molecule of bacterial origin (GO:0071219) | 0.043607 | 1 | FZD5;TNIP2 |
| regulation of type I interferon production (GO:0032479) | 0.044546 | 1 | POLR3GL;PQBP1 |
| regulation of myoblast fusion (GO:1901739) | 0.046396 | 1 | FLOT1 |
| regulation of skeletal muscle tissue development (GO:0048641) | 0.046396 | 1 | FLOT1 |
| alternative mRNA splicing, via spliceosome (GO:0000380) | 0.046396 | 1 | PQBP1 |
| negative regulation of substrate adhesion-dependent cell spreading (GO:1900025) | 0.046396 | 1 | AP1AR |
| positive regulation of establishment of protein localization (GO:1904951) | 0.046396 | 1 | CCT8 |
| negative regulation of metabolic process (GO:0009892) | 0.046396 | 1 | APOC1 |
| positive regulation of skeletal muscle tissue development (GO:0048643) | 0.046396 | 1 | FLOT1 |
| negative regulation of interleukin-1 secretion (GO:0050711) | 0.046396 | 1 | CPTP |
| phospholipid efflux (GO:0033700) | 0.046396 | 1 | APOC1 |
| positive regulation of T cell cytokine production (GO:0002726) | 0.046396 | 1 | FZD5 |
| negative regulation of fatty acid metabolic process (GO:0045922) | 0.046396 | 1 | APOC1 |
