# Supplementary material for: Ultra-high Magnification Endocytoscopy and Molecular Markers for Defining Endoscopic and Histologic Remission in Ulcerative Colitis—An Exploratory Study to Define Deep Remission
Source: Inflamm Bowel Dis. 2021 May 21;27(11):1719–30. doi: 10.1093/ibd/izab059 (PMC8528147; doi:10.1093/ibd/izab059)
Supplement: izab059_suppl_Supplementary_Table_Legends [file izab059_suppl_supplementary_table_legends.docx]

Supplementary Table 1: Differentially (Up and down) expressed genes in healed vs nonhealed mucosa as defined by A) ECSS, B) Mayo, C)RHI and D) Nancy scores

Supplementary Table 2: Differentially expressed genes common in healed mucosa defined by A) ECSS and RHI and B)ECSS and Nancy scores

Supplementary Table 3: Partial Least Square Discriminant Analysis (PLS/DA) of healed vs nonhealed mucosa defined by A)ECSS, B)Mayo, C) RHI and D) Nancy scores. Differentially expressed genes for which VIP > 1 are listed

Supplementary Table 4: AUC values of the 60 genes and corresponding p values for healing vs nonhealing defined by ECCS

Supplementary Table 5: KEGG pathway enrichment analysis of the differentially expressed genes defined by A) ECSS, B) Mayo, C) RHI and D) Nancy scores. Genes in pathways for which p 0.05 are listed.

Supplementary Table 6: Go Biological process enrichment analysis of the differentially expressed genes defined by A) ECSS, B) Mayo, C) RHI and D) Nancy scores. Genes in pathways for which p 0.05 are listed.

Supplementary Table 7: KEGG Pathway and GO Biological process enrichment analysis of genes commonly upregulated in healed mucosa defined by ECSS and RHI (A and B) and ECSS and Nancy (C and D) scores. The genes involved in each pathway are listed.
